# A systematic review of the effectiveness of dust control measures adopted to reduce workplace exposure

**DOI:** 10.1007/s11356-023-26321-w

**Published:** 2023-03-25

**Authors:** Frederick Anlimah, Vinod Gopaldasani, Catherine MacPhail, Brian Davies

**Affiliations:** 1 Faculty of the Arts, Social Sciences and Humanities, School of Health and Society, Centre for Occupational Public and Environmental Research in Safety and Health (COPERSH), University of Wollongong, Building 29, Wollongong, NSW 2522 Australia; 2 Faculty of the Arts, Social Sciences and Humanities, School of Health and Society, Centre for Occupational Public and Environmental Research in Safety and Health (COPERSH), University of Wollongong, Building 29, Room 124, Wollongong, NSW 2522 Australia; 3 Faculty of the Arts, Social Sciences and Humanities, School of Health and Society, University of Wollongong, Building 29, Room 242, Wollongong, NSW 2522 Australia; 4 Faculty of the Arts, Social Sciences and Humanities, School of Health and Society, Centre for Occupational Public and Environmental Research in Safety and Health (COPERSH), University of Wollongong, Building 29, Room 116, Wollongong, NSW 2522 Australia

**Keywords:** RCS, Silicosis, Exposure prevention, Personal protective equipment, PPE

## Abstract

The recent increase in silicosis cases in several countries casts doubt on dust control practices and their effectiveness in preventing respirable crystalline silica (RCS) exposure. Apart from silicosis, RCS may lead to other illnesses, health-related quality of life losses for workers and their families, and economic losses for companies. Thus, this systematic literature review examined the effectiveness of interventions employed to prevent exposure to RCS and increase the use of dust control measures. The review used keywords related to dust control interventions to search seven databases. Search results were screened and extracted for synthesis. The narrative synthesis showed the extent of research investment in China. In several designs and combinations, the interventions utilized water, surfactant, foam, and air currents to reduce dust exposure. These interventions offer varying degrees of dust control effectiveness against RCS and respirable dust. Although evidence indicates that interventions significantly decrease dust concentration levels, the control measures in place may not effectively prevent workplace overexposure to RCS. The review found that education and training interventions are employed to improve dust controls and respiratory protective equipment (RPE) use. Also, marketing strategies promote the use of RPE. These interventions can increase the frequency of use of RPE and the adoption of best practice dust control measures. Interventions increase knowledge, awareness, and attitudes about RPE usage and generate positive perceptions while reducing misconceptions. However, the benefits obtained from an intervention may diminish after its implementation, indicating that the interventions may not continually motivate workers to adopt control measures or use RPE.

**Supplementary Information:**

The online version contains supplementary material available at 10.1007/s11356-023-26321-w.

## Introduction

Work activities that move the earth, use sand-containing products, or disturb materials made from sand may expose workers to respirable crystalline silica (RCS). RCS refers to the mass fraction of dust, mainly formed from quartz, that can be inhaled into the respiratory tract. RCS inhalation is responsible for silicosis, an irreversible, progressive fibrotic lung disease with no cure (Greenberg et al. 2007; Simkhada 2017). RCS exposure can also lead to lung cancer (IARC 1997), pneumoconiosis (Hua et al. 2018), tuberculosis, chronic bronchitis, emphysema, and chronic obstructive pulmonary disease (Mason and Thompson 2010). RCS is a significant risk factor for developing immunologic and autoimmune diseases such as chronic renal disease (Steenland et al. 2001), monoclonal gammopathy and amyloidosis (Gołębiowski et al. 2022), Sjogren’s syndrome (Ziebelman et al. 2022), scleroderma (Haustein et al. 1990), rheumatoid arthritis, systemic lupus erythematosus, and sarcoidosis (Mason and Thompson 2010).

Ill-health is not the only outcome of dust or RCS exposure, as it threatens workers’ safety. The risk of an explosion increases with increasing dust levels (Su et al. 2020). Dust may reduce the vision of operators (Colinet et al. 2021) or settle on moving machine parts to increase wear and tear (Khorshid and Nawwar 1991), thereby accelerating equipment aging. Also, dust on work surfaces may lead to increased housekeeping labor costs due to frequent cleaning to avoid dust accumulation. These adverse effects of dust at the workplace may lead to economic losses for companies in legal fines, compensation for ill-health, lost output, and reduced productivity. Workers afflicted with silica dust–related illness and their families may suffer health-related quality of life losses.

Exposure prevention is the only way to prevent dust-related issues with Cecala et al. (2021), Colinet et al. (2010), and Colinet et al. (2021) highlighting some of the best practice controls available. These dust control measures range from simple work practices and respirators to sophisticated technologies. Top-level leadership, stakeholder involvement, targeted health risk management, standards, sustainable control measures, and training programs are examples of world-class practices (Cole 2017). These measures seemed to have initially reduced silicosis cases, but more recent mechanization has boosted productivity with a corresponding increase in dust generation (Le 2013; Ren et al. 2020). New forms of work like hydraulic fracturing (Hooker et al. 2013) and modern-day materials like artificial stone or countertops (Hoy et al. 2018) have introduced different sources of RCS. These conditions have resulted in a resurgence of silicosis (Kramer et al. 2012) and other related illnesses (Almberg et al. 2018) otherwise deemed controlled.

Recent cases of silicosis have been recorded in Spain (Pérez-Alonso et al. 2014), Israel (Shtraichman and Kramer 2017), Belgium (Ronsmans et al. 2019), Italy (Guarnieri et al. 2020), and China (Wu et al. 2020). In Australia, 57 silicosis cases and seven deaths were recorded in NSW for the 2020–2021 financial year (SafeWork NSW 2021). This resurgence of silicosis casts doubts on the effectiveness of dust control practices and the use of respirators in RCS exposure prevention. It is therefore important that the effectiveness of current practices is assessed. Attempts to evaluate dust control methods include a Delphi method in Korean construction sites by Noh et al. (2018) and a systematic review of controls for reducing coal workers’ pneumonoconiosis conducted by Ayaaba et al. (2017). These studies have made significant contributions but are limited to specific industries or are not systematic in design.

Also, while the effectiveness of respirators and other personal protective equipment (PPE) depends on how they are used and maintained, many workplaces report low PPE use compliance (Ebekozien 2021; Wright et al. 2019). To prevent exposure to RCS, it is therefore important to assess the interventions adopted to improve the use of respirators. Luong et al. (2016) studied the effects of behavioral interventions on respiratory protective equipment use in a systematic review. While their study was limited to education and training interventions, the present study explores all possible interventions to improve the use of respirators.

Thus, the study seeks to evaluate the effectiveness of dust control measures for controlling worker exposure across different industries and to identify interventions adopted to improve dust control and PPE use.

### Objectives

The aim of the research was achieved by answering the following questions:

- What dust control measures are adopted to control dust exposure at the workplace?
- How effective are RCS control measures in protecting workers against silica exposures?
- What strategies help workers improve the use of dust control measures and personal protective equipment?

## Methods

This study was performed according to the guidance provided in the Preferred Reporting Items for Systematic Reviews and Meta-Analyses (PRISMA) protocols (Moher et al. 2015). The review protocol was registered in the International Prospective Register of Systematic Reviews (PROSPERO) with the registration number (CRD42021254773).

### Sources of information

A systematic search was conducted on Web of Science, PubMed, Cochrane Library, EBSCOhost, ProQuest, Scopus, and Google Scholar to identify studies that addressed this study’s aims, published up to 14 October 2020. The reference list of all included studies and other relevant studies was also searched.

### Search strategy

An electronic search was conducted with keywords modified and adapted for each database. The search strategy was developed in consultation with a research librarian at the University of Wollongong and is presented in Supplementary Sheet 1.

### Eligibility criteria

Studies had to meet the following criteria to be eligible for the review:

- Be carried out at a workplace.
- The intervention is aimed at controlling dust or improving the use of dust control measures and or personal protective equipment (PPE).
- The impact of the intervention is evaluated, or the study includes a comparison group other than only experimental laboratory results or numerical simulation results.

Studies that had any of the following characteristics were excluded:

- Laboratory experiments or numerical simulation studies that were not tested and evaluated in the field
- Studies with field experiment results compared only with numerical simulation or laboratory experiments
- Studies that are published only as abstracts
- Interventions to control dust on roads other than a road network in a workplace
- Studies that did not report the outcome of interest
- Studies that only reported the challenges of dust control implementation
- Patents and theses
- Non-English publications

After the search was completed, time and language restrictions were placed as time, and other resources were limited to handle the number of citations returned. The authors agreed that studies published between 1 January 2010 and the search date would afford the identification of relevant and up-to-date dust control measures.

### Study selection

All records were imported into the EndNote reference manager, and duplicates were removed. One reviewer (FA) independently screened the returned search results based on the titles and abstracts. The peer-review status of each article was verified online. When this was impossible, study authors, journals, and publishers were asked about the peer-review status. Studies were excluded when it was not possible to determine their peer-review status. Full-text articles were obtained and reviewed independently by FA, with VG, CM, and BD contributing to the review when clarity was required. In cases of multiple publications from a single study, the study judged to be the original was extracted.

### Data collection process

FA independently extracted data from included studies using a developed Excel spreadsheet. VG, CM, and BD reviewed the extracted data independently, and any discrepancies were resolved through discussion. The extracted data included information about the workplace, study design, participants, interventions, outcomes, factors affecting the effectiveness of interventions, and each study’s main results.

### Risk of bias assessment

The quality of selected studies was appraised for potential bias using the Standard Quality Assessment Criteria for Evaluating Primary Research Papers (QualSyst) (Kmet et al. 2004). The QualSyst tool was selected as it is suitable for assessing the quality of studies with a broad range of designs. A QualSyst score > 0.800 was interpreted as strong quality, 0.500–0.800 as moderate quality, and < 0.500 as poor quality. This interpretation was adopted from Balaguer et al. (2020) and Speyer et al. (2018), where scores higher than 0.800 were rated as strong quality, 0.600–0.790 as good quality, 0.500–0.590 as adequate quality, and lower than 0.500 as poor quality. The modification was made to aid clarity and simplicity. The quality rating of studies was not used as an exclusion criterion.

### Risk of overexposure

The residual dust levels after the intervention implementation were used to rate the risk of RCS exposure for each study. Dust levels above 0.050 mg/m^3^ were rated as high risk, 0.025–0.050 mg/m^3^ were rated as moderate risk, and residual dust levels lower than 0.025 mg/m^3^ were rated as low risk. These ratings were adopted based on the threshold limit values (TLV) recommended by the American Conference of Governmental Industrial Hygienists (ACGIH) and the recommended exposure limit (REL) for RCS by the National Institute for Occupational Safety and Health (NIOSH). Similarly, the risk of respirable dust exposure was rated based on TLV, guidelines, and REL from ACGIH and NIOSH. Residual respirable coal dust below 0.400 mg/m^3^ were rated as low, levels between 0.400 and 1.00 mg/m^3^ as moderate risks, and levels above 1.00 mg/m^3^ as high risk. Residual respirable dust other than respirable coal and RCS (ORD) were rated as low risk when below 3.00 mg/m^3^ and high risk when above, as only ACGIH has a guideline value for this type of dust. These limits are based on research and epidemiological studies rather than economic or technological feasibility (Cecala et al. 2021), unlike regulatory limits, and are presented in Table 1.

**Table 1:** Recommended exposure guidelines used in the assessment of overexposure

Dust type	NIOSH (mg/m^3^)	Reference	ACGIH (mg/m^3^)	Reference
RCS	0.050	(NIOSH 2007)	0.025	(ACGIH 2020)
Respirable coal	1.00	(NIOSH 2019)	0.400	(ACGIH 2020)
Respirable dust (ORD)	-	(NIOSH 2018)	3.00	(NIOSH 2018)

It was not feasible to use a legal limit for the risk assessment as several countries have different legal requirements for different industries and dust types as illustrated in Table 2.

**Table 2:** Examples of occupational exposure limits in different countries

Country	Dust type	Scope	Legal limit (mg/m^3^)	Reference
USA	RCS	General industry and construction except agricultural operations	0.050	(OSHA 2016)
USA	Respirable quartz	Coal mines	0.100	(MSHA 2021)
USA	Respirable		1.50	
China	RCS $10.0\% \le \mathrm{free\;}{\mathrm{SiO}}_{2} \le 50.0\%$ $50.0\% <\mathrm{free\;}{\mathrm{SiO}}_{2} \le 80.0\%$ $\mathrm{free\;}{\mathrm{SiO}}_{2} >80$.0%	General industry	0.700	(NHC 2019)
			0.300	
			0.200	
China	Cement ($\mathrm{free\;}{\mathrm{SiO}}_{2}<10.0\%)$		1.50	
China	Respirable coal	Coal mines	2.50	
Australia	RCS	General industry	0.050	(MENA-Report 2020)
Australia	Respirable coal	Coal mines	1.50	

### Data synthesis and analysis

A narrative synthesis was presented for the included studies that were grouped by type of intervention and outcome. Conducting a meta-analysis was impossible as the studies were of various study designs.

### Outcome domains

The primary outcomes reported in this study relate to the reduction in RCS or particulate dust concentrations, dust diffusion or control distance, workplace visibility, and use or intention to use dust control measures or PPE. The level of awareness of dust controls or PPE and their use were also reported. Secondary outcomes relating to reduced risk of respiratory illness, improved lung function or decreased risk of workplace injury, and intention to fit check respirators were reported, but not used to conclude the effectiveness of interventions.

## Results

**Fig. 1:**
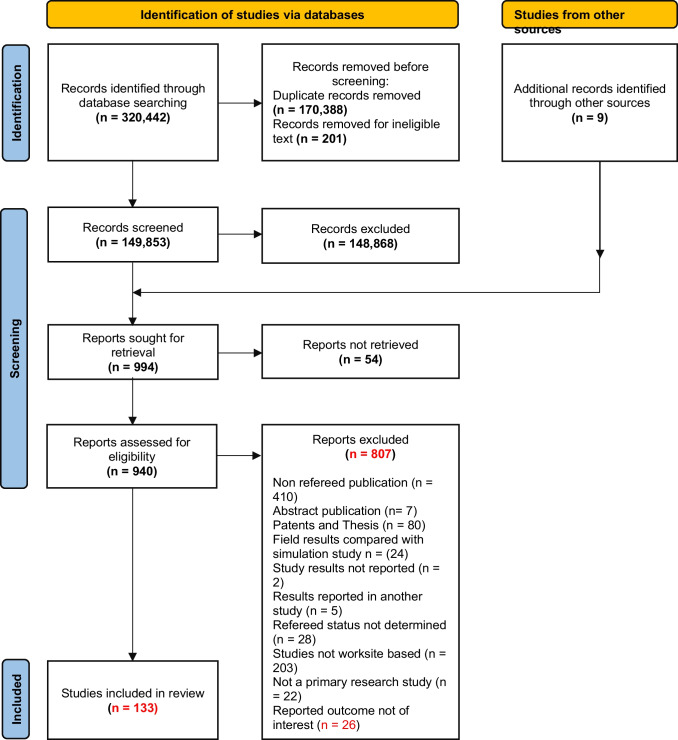
PRISMA flow diagram for the review

The electronic search returned 320,442 citations: 8748 from Web of Science, 14,694 from PubMed, 82,970 from Scopus, 6801 from Cochrane Library, 8568 from ProQuest, 150,388 from EBSCOhost, and 48,273 from Google Scholar. A total of 170,388 duplicate citations were identified, with 201 ineligible citations. Nine citations of potential relevance were identified by manually searching the bibliographies of included and other relevant articles. The full text of fifty-four potentially eligible studies could not be retrieved despite the assistance of the University of Wollongong’s library document delivery service team. At the same time, the peer-review status of twenty-eight citations could not be confirmed. After screening and applying the inclusion and exclusion criteria, 133 full-text studies were included in the review and extracted, as shown in Fig. 1.

### Study characteristics

**Fig. 2:**
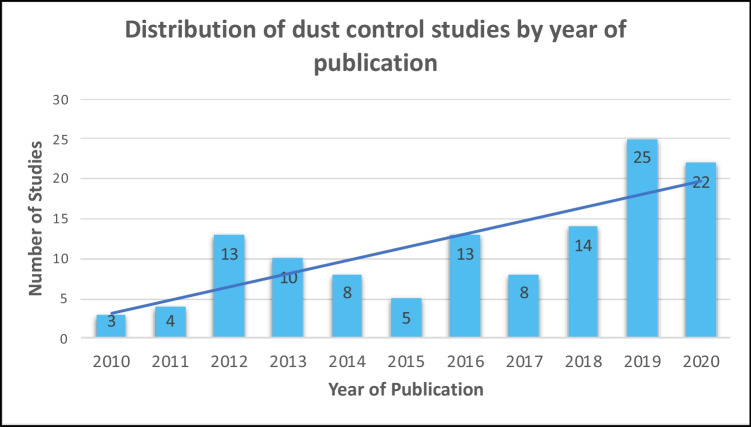
Yearly distribution of dust control studies

One study in the included studies assessed an intervention to improve the use of dust controls (Weidman et al. 2016), and seven studies researched ways to improve the use of respirators (Adewoye et al. 2014; Chen et al. 2019; Donham et al. 2011; Pounds et al. 2014; Robertsen et al. 2020; Shamsi et al. 2016; Woith et al. 2015). One hundred twenty-five studies evaluated dust control measures, as detailed in Supplementary Sheet 2. Of the studies that measured the effectiveness of dust control measures, 65.6% (82) were published after 2015, implying that the subject has recently received increased interest from scholars, with 25 papers published in 2019 (20.0%) and 22 in 2020 (17.6%) as displayed in Fig. 2.

Three studies (Adewoye et al. 2014; Donham et al. 2011; Pounds et al. 2014) published before 2015 evaluated interventions to improve the use of PPE, whereas four similar studies were published from 2015 onwards (Chen et al. 2019; Robertsen et al. 2020; Shamsi et al. 2016; Woith et al. 2015).

### Study settings

**Fig. 3:**
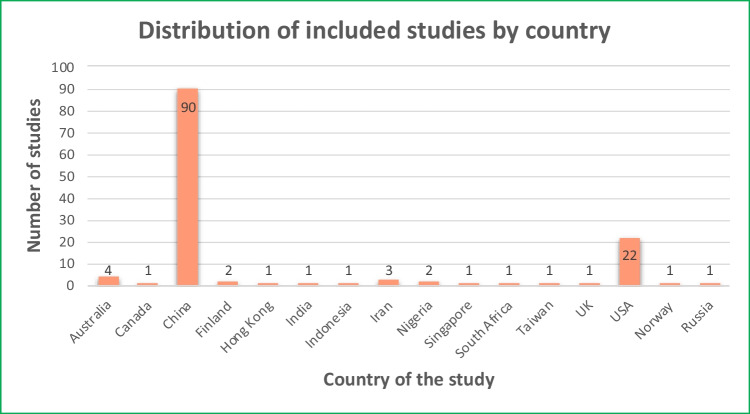
Distribution of included studies by country

Most studies were conducted in China (90) and the USA (22). Four studies were carried out in Australia, three in Iran, and two each from Finland and Nigeria, while Taiwan, South Korea, South Africa, Singapore, Netherlands, Indonesia, India, Hong Kong, the UK, and Canada each contributed a single study as highlighted in Fig. 3.

Studies measuring the effects of dust control interventions took place in 168 worksites, with one study not reporting the study site (Joy 2012). In four studies, evaluations were carried out in a field laboratory (Akbar-Khanzadeh et al. 2010; Fan et al. 2012; Shepherd and Woskie 2013; Summers and Parmigiani 2015). These studies were included because the field laboratories were enclosed workplace sections with expert tool operators cutting or drilling concrete materials as they would in their work operations. The enclosure was to minimize the effect of the wind (Fan et al. 2012) or to mimic concrete grinding in an enclosed space like basements of residential buildings (Akbar-Khanzadeh et al. 2010).

PPE improvement studies were completed across 932 registered small-scale electric arc welding workshops (Adewoye et al. 2014), two construction sites (Shamsi et al. 2016), fifty-nine small- and medium-sized Chinese enterprises exposed to solvents (Chen et al. 2019), six smelting plants (Robertsen et al. 2020), and a hospital (Woith et al. 2015). The rest of the studies were among farmers in the nine-county areas in Northwest Iowa (Donham et al. 2011), and the region of the Central States Center for Agricultural Safety and Health (CS-CASH) in Nebraska, USA (Pounds et al. 2014). At least 2514 participants participated in these studies.

### Study design

Most studies assessing the effectiveness of dust controls were quasi-experimental (122 studies), of which fifty-one studies were before and after in design. The remainder includes two cross-sectional studies and a longitudinal study. These studies cut across several industries and work activities. The construction and tunneling industry had twenty studies and ninety-two studies related to the mining and handling of mined ore. There were two studies each from stone processing, foundry, oil and gas, and manufacturing. A single study was conducted in a coal boiler plant, a laboratory, a thermal power plant, quarries, and a port shipping yard.

Before conducting field experiments, thirty-four studies utilized numerical simulation, theoretical analysis, or mathematical modeling to determine optimal operating factors. Similarly, twenty-two studies performed an initial numerical simulation plus laboratory experiments, while thirty-one conducted laboratory experiments before the field experiment. These methods were adopted to avoid trial-and-error practices in implementing dust controls.

Out of the seven PPE improvement studies, three were randomized controlled before and after studies (Adewoye et al. 2014; Chen et al. 2019; Robertsen et al. 2020), and two were quasi-experimental studies (Shamsi et al. 2016; Woith et al. 2015). The rest included a cohort study (Donham et al. 2011) and a cross-sectional study (Pounds et al. 2014).

### Study duration

Interventions were implemented for 4 weeks (Shamsi et al. 2016), 3 months (Lin et al. 2011), 6 months (Chen et al. 2019; Woith et al. 2015), a year and 2 months (Pounds et al. 2014), and 5 years (Donham et al. 2011). The remaining studies did not report the duration of the intervention implementation.

### Dust control interventions

**Fig. 4:**
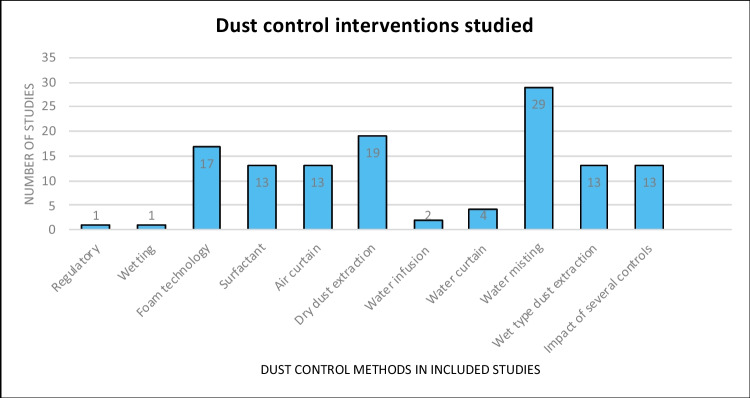
Range of measures adopted to control dust exposure at the workplace

The dust control interventions varied in design and implementation. Studies were classified under foaming systems (17 studies), surfactant (13), air curtain (13), dry dust extraction (19), water infusion (2), water curtain (4), water misting (29), wet dust extraction (13), and mixed control methods (13). Studies classified as mixed control methods evaluated more than one intervention in the study. The rest of the studies were classified as wetting (no atomisation) (Li et al. 2019a) and regulatory (Joy 2012). A summary of the dust control interventions is provided in Fig. 4, with more details in Supplementary Sheets 2 and 3.

While none of the studies evaluated the effect of regulatory instruments or enforcement in improving PPE use compliance, four studies adopted training or education and three implemented marketing interventions to improve PPE use.

### Training or education to improve the use of PPE

The studies that adopted training or education interventions include two studies with an intervention group and a control group (Adewoye et al. 2014; Donham et al. 2011), and two studies with two intervention groups and a control group (Chen et al. 2019; Robertsen et al. 2020). Apart from Adewoye et al. (2014), which implemented a health education program delivered through various educational methods and materials, the other three studies employed multifaceted interventions. The multi-component intervention adopted by Donham et al. (2011) consisted of education, clinical screening, on-farm safety audits, recognition, and cash incentives for attaining or retaining Certified Safe Farm status. The audit, among other things, assessed the use of PPE. In Chen et al. (2019), the first intervention group received safety education, a training package, and mHealth, while the second study group received peer education and the intervention of the first group. mHealth consisted of mobile messages to inform about safety risks and the use of respirators. The study by Robertsen et al. (2020) provided fit testing for the first intervention group and fit testing complemented with training on exposure risk and effects of smelting for the second intervention group.

### Marketing to motivate the use of RPE

Three studies adopted an intervention that used marketing to cue the use of RPE. The intervention group in (Shamsi et al. 2016) received free helmets, dust masks, and safety gloves. PPE use was promoted through personal communication and stickers with safety messages fixed on the helmets. Some workers received pamphlets that had messages related to the merits of using PPE and the risks they can reduce, while workers who could not read had face-to-face counseling. The control group did not receive any intervention.

The remaining studies did not have any control group. In Pounds et al. (2014), farmers were given PPE carrying bags inscribed with motivational messages and YouTube videos on how to use respirators. The farmers received emails and were engaged during farm shows to increase their awareness and to direct them to the YouTube videos. Woith et al. (2015) conducted a photovoice intervention in which selected workers photographed someone or something important to them with a short text about why they are motivated to wear and fit-check respirators. A poster was designed with the photograph and text and displayed in the work area of the employee.

### Training or education to improve use of dust control tools

Weidman et al. (2016) was the only study that involved an intervention promoting the use of dust control interventions other than PPE. This study was a controlled before-after study conducted on a renovation site among drywall-finishing workers. The intervention utilized training, cues to action, and a trial-ability strategy. The didactic and interactive sessions created awareness about drywall dust hazards and had hands-on practice with a ventilated sander. Hard hat stickers and printed T-shirts were used to draw attention to the need to protect against respirable crystalline silica. The control group did not receive any intervention.

### Reported outcome measures

The effect of dust control interventions was mainly reported as objective measurements. Ninety-one (72.8%) of dust control studies reported reduced respirable or silica dust concentrations, while sixteen (12.8%) reported only total or airborne dust. In eighteen (14.4%) studies, more information was needed for the type of dust controlled. The term “respirable dust” refers to the portion of dust particles that can penetrate the airways and reach the lungs, while “total dust” refers to all dust particles contained in a volume of air (ISO 1996).

Some studies reporting dust concentration levels also measured dust diffusion or control distance (Liu et al. 2019a, b, 2018a, b; Zhou et al. 2020a), workplace visibility (Fang et al. 2019; Guo et al. 2019; Li et al. 2017; Lu et al. 2015a, b; Wang et al. 2011; Yin et al. 2019), and lung function (Lin et al. 2011). Three studies were judged to evaluate the outcome of the intervention studied by observation (He et al. 2018; Roberts and Wypych 2017; Yin et al. 2013). Two studies reported the visible presence of dust, while Roberts and Wypych (2017) gave a numerical value, although evidence of dust measurement was not presented.

Dust concentration levels were recorded with several dust sampling tools and techniques, usually at the breathing zones of workers as captured in Supplementary Sheet 3 and at various positions in the workplace. The most common measuring position was near the equipment operator.

The outcome measures reported in PPE use improvement studies were self-reported (Adewoye et al. 2014; Chen et al. 2019; Donham et al. 2011; Pounds et al. 2014; Robertsen et al. 2020; Woith et al. 2015) or a combination of self-reporting and field observation (Shamsi et al. 2016). Weidman et al. (2016), objectively reported self-efficacy, trust in technology, and adoption readiness for a ventilated sander.

### Use or intention to use PPE

In Shamsi et al. (2016), the use of dust masks was reported 6 weeks after the start of the intervention. The use of PPE was also reported by Adewoye et al. (2014) after 4 months of the intervention phase. In contrast, the same outcome domain was reported by Donham et al. (2011) after 5 years of the Certified Safe Farm intervention. In Chen et al. (2019), the appropriate use of RPE by internal migrant workers was reported for 3 and 6 months of intervention. The intention to wear or fit-check respirators was reported by (Woith et al. 2015) after the 6-month intervention period, and the intention to use RPE was described by Pounds et al. (2014) and Robertsen et al. (2020).

### Knowledge, perceptions, awareness, and PPE use practices

In Robertsen et al. (2020), the outcomes of the study were reported 2 weeks after the intervention and related to the knowledge of workers regarding RPE use and their attitudes toward its use. Similarly, self-reported knowledge about RPE was measured by Pounds et al. (2014). Adewoye et al. (2014), Chen et al. (2019); Shamsi et al. (2016), and Woith et al. (2015) measured PPE awareness levels, attitudes toward PPE use, and attitudes and perceptions about wearing PPE, respectively.

### Secondary outcome measures

Secondary outcomes included in this review include the risk of organic dust toxic syndrome measured by Donham et al. (2011), participation in occupational health check-ups during the past 6 months (Chen et al. 2019), and self-efficacy to wear or fit-check respirators and self-perceived state of health and perceptions about wearing or fit-checking respirators were reported in Woith et al. (2015).

### Effectiveness of dust control interventions

**Fig. 5:**
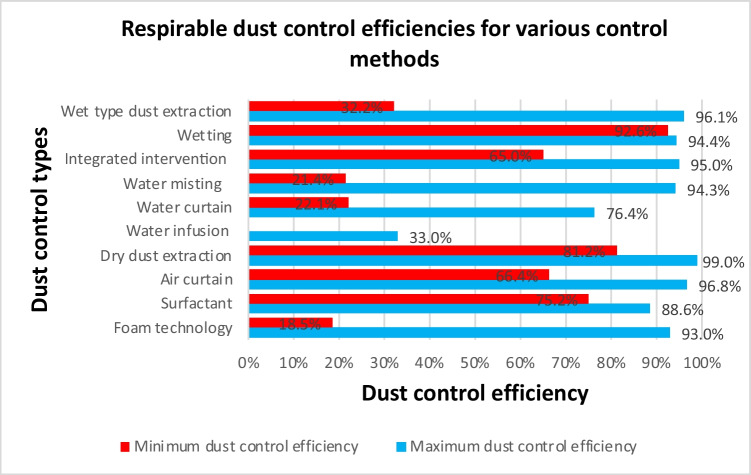
Range of respirable dust fall rates for the various dust control categories

**Fig. 6:**
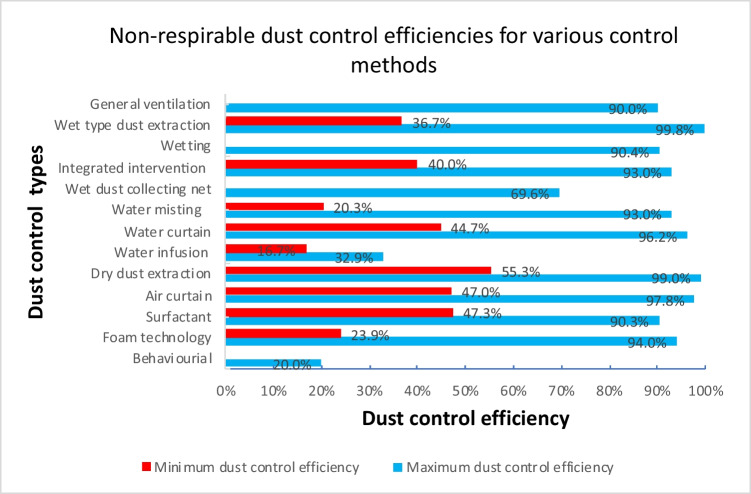
Range of dust fall rates for the various dust control categories

The implemented dust control interventions produced varied reductions with water misting, foaming, and wet dust extraction interventions offering the widest range of control. The total dust fall rate ranged from 30.3–93% for water misting, 23.9–94% for foaming, and 36.7–99.04% for wet dust extractions. Respirable dust control ranges were 18.5–93% for foaming, 21.4–94.3% for water misting, and 32.2–96.1% for wet dust extraction. Notable studies that reported high total dust control levels above 95% adopted wet dust extraction (Li et al. 2020a, b; Warden and Warden 2019; Xia et al. 2016; Zhou et al. 2013), dry dust collection or extraction (Alexander et al. 2018; Lin et al. 2014; Zhang et al. 2014), air curtain (Liu et al. 2018b; Yin et al. 2019), and water curtain interventions (Peng et al. 2020a). Regarding respirable dust, studies that reported efficiencies above 95% were dry dust extraction and collection systems (Akbar-Khanzadeh et al. 2010; Alexander et al. 2016, 2018; Li et al. 2019b; Zarei et al. 2018), air curtain (Zhou et al. 2012a), and wet dust extraction intervention (Zhou et al. 2013). Water infusion produced the lowest dust control efficiencies ranging from 16.7 to 33.8% (Cheng et al. 2012a; Hu et al. 2016). These studies were conducted in hard coal seams with low permeability, high gas content, or underdeveloped joints and fissures, which may have affected efficiency. Figures 5 and 6 showcase the dust removal rates for respirable and total dust, respectively.

### Improving the use of dust controls and PPE usage

Weidman et al. (2016) found a significant difference between the intervention and control groups for adoption readiness for ventilated sanders, but a follow-up showed no significant difference between both groups. The implementation of an intervention resulted in a significant increase in the use of respirators (Adewoye et al. 2014; Donham et al. 2011; Shamsi et al. 2016; Woith et al. 2015). On the other hand, the adopted interventions did not increase the use of respirators in Robertsen et al. (2020).

The intention to use respirators increased in Pounds et al. (2014) and Robertsen et al. (2020). The studies showed that interventions can promote the proper use of respirators (Chen et al. 2019) and increase their frequency of use (Pounds et al. 2014).

### Knowledge and attitudes toward PPE usage

PPE interventions also increased knowledge and attitudes toward RPE and decreased the perception of inconvenience associated with RPE use (Robertsen et al. 2020), positively increasing attitude toward RPE use (Chen et al. 2019) while improving the perception that PPE use keeps workers safe (Woith et al. 2015). Thus, training-based interventions can increase the awareness levels required to safely carry out specific jobs (Adewoye et al. 2014).

### Secondary outcomes

The studied interventions increased occupational health check-ups (Chen et al. 2019), respirator fit-checking, and knowledge about the health effects of workplace exposures and the need to use respirators (Chen et al. 2019; Pounds et al. 2014). In Weidman et al. (2016), there were no significant improvements in the intervention group for health knowledge, perceived risk to health, or trust in organization constructs compared to the control group. The intervention increased self-efficacy and trust in technology. Studies also reported a reduced prevalence of organic dust toxic syndrome (Donham et al. 2011).

### Risk of bias and dust overexposure rating

The overview of the risk of bias, based on the QualSyst tool, ranged from “poor” to “strong.” The quality of nine dust reduction studies (7.20%) was graded as “poor,” seventy-four studies (59.2%) as “moderate,” and forty-two (33.6%) as “strong.” PPE improvement studies were rated as “moderate” quality (82.0%) and as “poor” quality (18.0%). The single study on an intervention to promote the usage of dust control measures was graded as “moderate” with a QualSyst Score of 0.710.

The evaluation of the risk of overexposure to RCS, respirable coal dust, and other respirable dust revealed that most of the implemented interventions were not successful in reducing these levels to acceptable limits. Out of the twenty studies that reported RCS concentrations, one (5.00%) indicated a low risk, eight (40.0%) a moderate risk, and eleven (55.0%) a high risk of overexposure. Similarly, out of the fifty-eight studies that reported respirable coal concentrations, four (6.90%) indicated a low risk, two (3.45%) showed a moderate risk, and forty-six (79.3%) reported a high risk of overexposure. Moreover, out of the seventeen studies that reported concentrations of other respirable dust, nine (52.9%) indicated a low risk and five (29.4%) a high risk of overexposure. This demonstrates that most of the interventions failed to bring dust levels below 0.050 mg/m^3^ (RCS) and 1.00 mg/m^3^ (respirable coal dust).

In forty-three of the studies, insufficient information was available to assess the risk of overexposure, including six that looked at respirable coal and three that focused on other respirable dust. Tables 3 and 4 provide more information on the methodological quality of each study and exposure rating after an intervention was used at a workplace.

**Table 3:** Quality assessment of dust control studies and evaluation of interventions

Study	QualSyst score	Quality rating	Dust type	Dust Control efficiency	Residual dust (mg/m^3^)	Risk rating
(Bao et al. 2020)	0.730	Moderate	Total	Not rated	115	Not rated
(Cai et al. 2020)	0.770	Moderate	Total	90.5%	208	Not rated
(Cheng et al. 2020)	0.820	Strong	Total	78.0%	1.30	Not rated
(Guo et al. 2020)	0.820	Strong	R Coal^1^	91.3%	5.10	High
(Hu et al. 2020)	0.680	Moderate	ORD^2^	57.0%	0.049	Low
(Hua et al. 2020)	0.820	Strong	Not stated	82.9%	50.0	Not rated
(Li et al. 2020a)	0.730	Moderate	R Coal	97.0%	4.30	High
(Li et al. 2020b)	0.500	Moderate	R Coal	67.0%	2.40	High
(Liu et al. 2020)	0.770	Moderate	R Coal	80.0%	1.00	Moderate
(Louk et al. 2020)	0.680	Moderate	RCS	Not rated	0.060	High
(Ma et al. 2020)	0.820	Strong	Total	90.3%	87.2	Not rated
(Patts et al. 2020)	0.460	Poor	Not stated	20.0%	Not stated	Not rated
(Peng et al. 2020a)	0.820	Strong	R Coal	95.7%	0.075	Low
(Peng et al. 2020b)	0.730	Moderate	Total	77.9%	60.2	Not rated
(Reed et al. 2020a)	0.820	Strong	R Coal	60.0%	0.050	Low
(Reed et al. 2020b)	0.680	Moderate	R Coal	60.0%	0.258	Low
(Ren et al. 2020)	0.680	Moderate	R Coal	85.2%	Not stated	Not rated
(Wang et al. 2020)	0.770	Moderate	R Coal	95.0%	12.4	High
(Xu et al. 2020)	0.770	Moderate	R Coal	89.3%	30.0	High
(Zhou et al. 2020a)	0.730	Moderate	R Coal	79.1%	8.00	High
(Zhou et al. 2020b)	0.680	Moderate	Total	> 80.4%	< 2.00	Not rated
(Zhu et al. 2020)	0.910	Strong	R Coal	86.0%	3.90	High
(Chen and Liu 2019)	0.770	Moderate	Total	90.0%	17.8	Not rated
(Fang et al. 2019)	0.820	Strong	R Coal	80.8%	12.7	High
(Ge et al. 2019)	0.730	Moderate	Total	93.0%	32.0	Not rated
(Gottesfeld et al. 2019)	0.640	Moderate	RCS	80.0%	0.062	High
(Guo et al. 2019)	0.820	Strong	R Coal	76.0%	25.5	High
(Hu et al. 2019)	0.820	Strong	R Coal	76.4%	50.4	High
(Kokkonen et al. 2019)	0.860	Strong	ORD	90.0%	0.250	Low
(Li et al. 2019a)	0.770	Moderate	R Coal	90.0%	1.30	High
(Li et al. 2019b)	0.730	Moderate	R Coal	97.8%	2.14	High
(Liu et al. 2019a)	0.820	Strong	Total	72.1%	5.59	Not rated
(Liu et al. 2019b)	0.730	Moderate	Total	Not rated	18.2	Not rated
(Lu et al. 2019)	0.820	Strong	R Coal	87.7%	79.3	High
(Peng et al. 2019a)	0.730	Moderate	Total	88.0%	96.9	Not rated
(Peng et al. 2019b)	0.820	Strong	${\mathrm{PM}}_{10}$	91.5%	0.987	Not rated
(Reed et al. 2019)	0.770	Moderate	Total	91.0%	0.614	Not rated
(Sun et al. 2019)	0.770	Moderate	R Coal	80.7%	35.4	High
(Wang et al. 2019a)	0.770	Moderate	R Coal	80.9%	22.1	High
(Wang et al. 2019b)	0.820	Strong	R Coal	86.0%	64.5	High
(Wang et al. 2019c)	0.730	Moderate	R Coal	88.0%	5.24	High
(Wang et al. 2019d)	0.640	Moderate	ORD	65.0%	62.0	High
(Warden and Warden 2019)	0.590	Moderate	Total	95.3%	0.344	Not rated
(Xu et al. 2019)	0.730	Moderate	R Coal	83.0%	38.0	High
(Yang et al. 2019)	0.730	Moderate	Not stated	90.1%	80.5	Not rated
(Yin et al. 2019)	0.680	Moderate	Not stated	97.8%	5.00	Not rated
(Zhou et al. 2019)	0.730	Moderate	R Coal	88.6%	15.1	High
(Alexander et al. 2018)	0.860	Strong	RCS	99.0%	0.028	Moderate
(Chen et al. 2018)	0.730	Moderate	Not stated	69.6%	< 40.0	Not rated
(Firdaussyah and Suryo 2018)	0.640	Moderate	RCS	Not rated	108	High
(Gao et al. 2018)	0.680	Moderate	R Coal	23.0%	6.51	High
(Han and Liu 2018)	0.770	Moderate	R Coal	86.5%	48.2	High
(He et al. 2018)	0.320	Poor	ORD	Not rated	Not stated	Not rated
(Kanjiyangat and Hareendran 2018)	0.820	Strong	${\mathrm{PM}}_{5}$ Coal	81.0%	1.60	High
(Liao et al. 2018)	0.820	Strong	R Coal	87.7%	29.8	High
(Liu et al. 2018b)	0.680	Moderate	Not stated	98.8%	9.45	Not rated
(Sun et al. 2018)	0.820	Strong	R Coal	26.7%	50.0	High
(Wang et al. 2018)	0.770	Moderate	R Coal	79.4%	73.8	High
(Zarei et al. 2018)	0.820	Strong	RCS	96.0%	0.044	Moderate
(Zhou et al. 2018a)	0.770	Moderate	R Coal	61.8%	21.0	High
(Zhou et al. 2018b)	0.820	Strong	R Coal	85.0%	10.6	High
(Kokkonen et al. 2017)	0.820	Strong	${\mathrm{PM}}_{10}$	Not rated	19.0	Not rated
(Li et al. 2017)	0.820	Strong	ORD	87.3%	28.9	High
(Lu et al. 2017)	0.820	Strong	R Coal	88.3%	59.2	High
(Nie et al. 2017)	0.820	Strong	R Coal	70.4%	38.3	High
(Roberts and Wypych 2017)	0.410	Poor	Airborne	100%	Not stated	Not rated
(Zhou et al. 2017a)	0.680	Moderate	R Coal	90.0%	8.00	High
(Zhou et al. 2017b)	0.730	Moderate	R Coal	91.1%	4.57	High
(Zhou et al. 2017c)	0.680	Moderate	R Coal	83.5%	Not stated	Not rated
(Alexander et al. 2016)	0.820	Strong	RCS	99.0%	0.053	High
(Cheng et al. 2016)	0.770	Moderate	R Coal	70.0%	33.0	High
(Du Plessis et al. 2016)	0.770	Moderate	ORD	47.3%	0.219	Low
(Echt et al. 2016)	0.860	Strong	RCS	> 90.0%	3.90	High
(Han et al. 2016)	0.770	Moderate	R Coal	82.6%	62.3	High
(Hu et al. 2016)	0.680	Moderate	Not stated	25.7%	33.5	Not rated
(Nie et al. 2016a)	0.820	Strong	Not stated	94.4%	14.8	Not rated
(Nie et al. 2016b)	0.820	Strong	R Coal	80.9%	15.2	High
(Qi and Lo 2016)	0.820	Strong	RCS	Not rated	0.021	Low
(Wang et al. 2016a)	0.820	Strong	R Coal	88.7%	81.3	High
(Wang et al. 2016b)	0.680	Moderate	Not stated	93.6%	300	Not rated
(Wang et al. 2016c)	0.820	Strong	R Coal	92.3%	75.9	High
(Xia et al. 2016)	0.410	Poor	Total	96.4%	2.80	Not rated
(Chen et al. 2015)	0.730	Moderate	R Coal	72.6%	Not stated	Not rated
(Lu et al. 2015a)	0.820	Strong	R Coal	88.1%	113	High
(Lu et al. 2015b)	0.820	Strong	Not stated	86.5%	136	Not rated
(Summers and Parmigiani 2015)	0.680	Moderate	RCS	Not rated	< 0.050	Moderate
(Wang et al. 2015)	0.680	Moderate	R Coal	85.9%	60.1	High
(Garcia et al. 2014)	0.650	Moderate	RCS	> OSHA PEL*^3^	1.46	High
(Han et al. 2014)	0.770	Moderate	R Coal	66.9%	119	High
(Lin et al. 2014)	0.550	Moderate	Not stated	98.2%	0.750	Not rated
(Ren et al. 2014a)	0.730	Moderate	R Coal	68.0%	0.400	Moderate
(Ren et al. 2014b)	0.550	Moderate	ORD	69.0	Not stated	Not rated
(Shang 2014)	0.410	Poor	Not stated	> 40.0%	0.470	Not rated
(Wang et al. 2014)	0.910	Strong	R Coal	85.8%	53.5	High
(Zhang et al. 2014)	0.360	Poor	Not stated	98.8%	Not stated	Not rated
(Colinet et al. 2013)	0.730	Moderate	R Coal	91.0%	2.05	High
(Morteza et al. 2013)	0.820	Strong	RCS	81.8%	0.043	Moderate
(Ren et al. 2013)	0.770	Moderate	R Coal	30.0%	0.190	Low
(Shepherd and Woskie 2013)	0.860	Strong	RCS	92.4%	0.335	High
(Shi et al. 2013)	0.680	Moderate	ORD	80.3%	0.080	Low
(Wallace and Cheung 2013)	0.640	Moderate	ORD	Not rated	0.020	Low
(Wang et al. 2013)	0.820	Strong	R Coal	84.4%	84.3	High
(Yin et al. 2013)	0.320	Poor	Not stated	Not rated	Not stated	Not rated
(Zhou et al. 2013)	0.640	Moderate	R Coal	96.1%	6.40	High
(Zongyin 2013)	0.450	Poor	Not stated	90.0%	< 3.00	Not rated
(Cheng et al. 2012a)	0.640	Moderate	ORD	96.8%	9.80	High
(Cheng et al. 2012b)	0.730	Moderate	R Coal	33.8%	50.0	High
(Cooper et al. 2012)	0.860	Strong	RCS	94.3%	0.040	Moderate
(Fan et al. 2012)	0.820	Strong	RCS	42.9%	0.040	Moderate
			ORD	63.0%	0.100	Low
(Jian et al. 2012)	0.680	Moderate	ORD	21.4%	11.0	High
(Joy 2012)	0.640	Moderate	RCS	Not rated	> 0.100	High
(Middaugh et al. 2012)	0.820	Strong	RCS	78.1%	0.210	High
			ORD	78.0%	3.60	High
(Ren et al. 2012)	0.680	Moderate	R Coal	68.9%	Not stated	Not rated
(Wang et al. 2012)	0.680	Moderate	R Coal	84.4%	84.3	High
(Xie et al. 2012)	0.770	Moderate	Not stated	95.0%	12.6	Not rated
(Zhang et al. 2012)	0.270	Poor	Not stated	Not rated	21.7	Not rated
(Zhou et al. 2012a)	0.640	Moderate	R Coal	92.5%	Not stated	Not rated
(Zhou et al. 2012b)	0.680	Moderate	R Coal	96.1%	6.40	High
(Du et al. 2011)	0.730	Moderate	Not stated	Not rated	4.90	Not rated
(Lin et al. 2011)	0.540	Moderate	RCS	58.1%	0.180	High
			ORD	44.3%	1.60	Low
(Potts and Reed 2011)	0.770	Moderate	ORD	81.0%	0.190	Low
(Wang et al. 2011)	0.680	Moderate	R Coal	85.4%	Not stated	Not rated
(Akbar-Khanzadeh et al. 2010)	0.860	Strong	RCS	99.0%	0.110	High
			ORD	98.9%	0.670	Low
(Gurley et al. 2010)	0.770	Moderate	RCS	25.0%	0.030	Moderate
(Hedges et al. 2010)	0.730	Moderate	RCS	57.1%	0.030	Moderate

^1^Respirable coal

^2^Other respirable dust not RCS or respirable coal

^3^Occupational Safety and Health Administration permissible exposure limits (OSHA PEL) is the legal exposure limit for respirable crystalline silica in the USA. At the time of the study

**Table 4:** Quality assessment of dust control and PPE use improvement studies

Study	QualSyst score	Quality rating
(Robertsen et al. 2020)	0.750	Moderate
(Chen et al. 2019)	0.790	Moderate
(Shamsi et al. 2016)	0.710	Moderate
(Woith et al. 2015)	0.570	Moderate
(Adewoye et al. 2014)	0.710	Moderate
(Pounds et al. 2014)	0.430	Poor
(Donham et al. 2011)	0.710	Moderate
(Weidman et al. 2016)	0.710	Moderate

$$\mathrm{respirable\; PEL}= \frac{250\mathrm\;{mppcf}}{\%\;\mathrm{Silica}+5}$$

## Discussion

The review identified 133 articles consisting of 125 studies that evaluated dust control interventions, seven studies that investigated ways to improve the use of respirators, and one study that promoted the use of a dust control intervention. The reviewed papers showed an increasing research interest in dust reduction, mainly in China, and found various interventions adopted across industries to reduce worker exposure to dust. The studies varied in research methodologies and design, but the QualSyst quality assessment tool made it possible to assess the risk of bias across all designs.

### Dust control interventions

The identified dust control interventions either exerted a direct or an indirect effect in reducing worker exposure. The only article that studied an indirect approach to dust control assessed the impact of USA’s Mine Safety and Health Administration regulatory instrument on dust control and was published before regulatory changes in 2016. Regardless, the results of this study with moderate risk of bias suggest that the regulatory influence was not adequate in preventing miners’ exposure above respirable time-weighted average (TWA) quartz concentration of 0.100 mg/m^3^. This may justify Ayaaba et al. (2017), who found little evidence to support compliance and effectiveness of permissible exposure limits (PEL) in the coal mining industry. While the adherence to dust or RCS legal limits may not be satisfactory, it is interesting to note that the studies compared their results to different legal limits in the various countries of study. Due to disparities in legal limits for different countries, compliance in one country may be a legal breach in another. For example, 2.4 mg/m^3^ residual dust in Li et al. (2020b) meets the 2.5 mg/m^3^ legal limit for coal dust in China but might be legally unsafe in the USA due to the 1.5 mg/m^3^ legal limit (MSHA 2021). Ultimately, this implies that exposure protection is not the same across countries. This difference in regulations is also highlighted by Ji et al. (2016) after comparing longwall dust control measures in Australia and China.

Water is mainly used to control dust exposure directly and is usually achieved by using nozzles to atomise the water into particles close in size to the dust. The nozzle type, position, and arrangement relative to the dust sources are critical to capture the dust effectively. Water misting interventions may be combined with air current for dust control, as demonstrated in the reviewed studies such as Peng et al. (2019b). Water is also used in water infusion, pumped under pressure to increase the Earth’s crust’s water content before mining. To improve the dust-capturing capacity of water, a surfactant may be added to reduce the contact angle of water when in contact with dust particles. The environmentally friendly surfactants identified in the review significantly improve dust control.

An alternative to water is using foam to provide a large surface area for dust capturing. Studies that evaluated foam dust control showcased that novel foaming devices, which automatically draw in the foaming agent and do not require any electrical power to operate, produce more stable foams and are better suited for mitigating explosion hazards in underground settings.

Like water, the air is widely used to control dust and is mainly used to disperse, extract, or create an air curtain to prevent dust dispersion. Several designs combine air and water misting to produce dust control interventions. Integrated interventions can be achieved by combining the foam-water misting study (Wang et al. 2020) and surfactant magnetized water (Zhou et al. 2017c, 2018b).

Most of the interventions were complex and required careful consideration in design and testing. However, the studies by Gottesfeld et al. (2019) and Lin et al. (2011) demonstrated that simple engineering solutions such as using commercially available nozzles, general ventilation, and awareness training could significantly reduce RCS levels. Numerical simulations and laboratory experiments were carried out in the included studies to aid in designing and testing complex interventions. The numerical and experimental laboratory studies produced results like those obtained in field tests, proving cost-effective. This finding is consistent with other studies not included in the review, which have shown that numerical simulations can effectively simulate dust control effects in tunnels and underground mine headings (Cai et al. 2019; Hua et al. 2018; Liu et al. 2018a, 2019c).

Although most studies focused on the impact of engineering controls, Lin et al. (2011) showcased in the helmet-CAM study that by identifying and contributing factors such as worker behavior that influence the effective use of controls, exposure can be reduced by more than 20%.

### Effectiveness of dust control measures

Like Radnoff et al. (2015), the review found that dust control interventions offer a comprehensive range of dust fall rates. While some interventions were not successful in reducing dust levels (Firdaussyah and Suryo 2018; Garcia et al. 2014; Kokkonen et al. 2017; Qi and Lo 2016; Shepherd and Woskie 2013), others successfully achieved low residual dust profiles after implementation, as shown in Table 3 (Qi and Lo 2016; Wallace and Cheung 2013). This confirms the potential of dust control measures to reduce worker exposure levels to prevent silicosis.

In most dust control studies, the risk of overexposure after implementing an intervention was high, although the interventions produced high dust control reductions. For example, the efficiencies recorded by the interventions in Akbar-Khanzadeh et al. (2010) (99.0%) and Shepherd and Woskie (2013) (92.4%) resulted in residual RCS levels of 0.110 mg/m^3^ and 0.335 mg/m^3^, respectively. These levels still pose a high risk of overexposure to RCS. A similar observation is made in studies that controlled respirable coal dust, such as Li et al. (2019b) with 97.8% efficiency and 2.14 mg/m^3^ residual concentration. Another example is shown in Wang et al. (2020) where an efficiency of 95.0% still exposed workers to 12.4 mg/m^3^ of respirable coal dust. This result demonstrates that even when interventions significantly reduce dust levels, there is still a high risk of overexposure.

Although the moderate methodological quality of these studies necessitates caution in interpretation, the review finding demonstrates that without dust controls, the work environment will be hazardous to workers’ safety and health. However, some interventions can lower dust levels to a safe level, while the majority are not able to. These varying levels of effectiveness are dependent on several factors (Chen et al. 2018). For example, the spray pressure (Guo et al. 2020), the spray particle size (Ma et al. 2020), and the droplet concentration (Li et al. 2020a, b) influence the dust control effect of a water misting system. Supplementary Sheet 4 highlights the factors influencing the effectiveness of the studied interventions. Some of these factors are like factors identified by Prostański and Vargová (2018) and Xu et al. (2018).

### Strategies to increase the use of dust control measures and PPEs

The review did not find any study on how regulations promote the use of dust controls or PPE. Included studies exploited education, training, and marketing strategies to cue the use of dust control measures or RPE. These strategies were usually multifaceted and required formative research to design. Results from the review suggest that adopted interventions increase knowledge, awareness, and attitudes about PPE usage and generate positive perceptions about PPE usage and its health and safety benefits while reducing misconceptions. The studied interventions increased the intention to use PPE and the proper use of respirators, but the demographic characteristics of participants, such as gender, language, or income, did not influence the use of the PPE.

Some studies found PPE use to increase, while others found no significant rise after the study. Specifically, Adewoye et al. (2014), Donham et al. (2011), Shamsi et al. (2016), and Woith et al. (2015) reported an increase in the use of respirators, but Robertsen et al. (2020) found no significant increase. Robertsen et al. (2020) suggest that physical challenges with the use of respirators such as sweating and communication issues could be a factor as to why the intention to use may not translate into actual use.

While some interventions demonstrated gains after a short period of implementation, Chen et al. (2019) did not report any significant difference in the outcome measure at 3 months but recorded differences at 6 months. Although these interventions were successful in increasing knowledge, awareness levels, and adoption readiness of dust controls and RPE, the benefits were only short term as indicated by (Weidman et al. 2016). This suggests that completed interventions may not be effective in providing sustained motivation to use dust controls and respirators.

### Quality of the evidence

Generally, the risk of bias was moderate in included studies, and there is the need for better quality studies which is possible, as demonstrated in some studies. Notwithstanding, there is a need for better methodological quality assessment tools to evaluate the quality of reviews having mixed categories of research methods and methodology.

### Implications for practice

Overall, the review indicates that dust control measures provide wide-ranging benefits in dust reduction. By adequately designing and controlling the factors influencing efficiencies, interventions can be used to reduce worker exposure to dust. Although the evidence is generally of moderate risk of bias, it suggests that current interventions may not safely reduce worker exposure to silica. Therefore, implementing multiple layers of controls is highly recommended, and dust-generating tasks should not be performed without any engineering control.

While the evidence is of moderate quality, education, training, and marketing strategies can increase awareness and use of dust control interventions and respirators. Interventions should be infused into the work processes to have a lasting effect. Countries with no established limits for workplace dust exposure should establish appropriate standards. A harmonized protection standard must also be considered.

### Implications for research

While interventions drastically reduce dust levels, the level of residual dust may still endanger workers. Thus, more case–control research is required to improve the efficiency of dust control methods. The use of numerical simulation to enable the consideration of several workplace factors is recommended. Bias assessment tools adequate to evaluate qualitative, quantitative, and mixed method studies in a single review should be studied.

## Conclusion

Due to the resurgence of silicosis in numerous countries, the review assessed the effectiveness of dust control measures in protecting workers against exposure to respirable crystalline silica and identified strategies to increase the use of dust control measures and respirators. The review shows the extent of research investment in China in reducing exposure to RCS. Dust control measures differ in design and application and offer different degrees of effectiveness. The review suggests that regulatory influence is inadequate in preventing miners’ overexposure. The disparities in legal limits for different countries imply that protection from overexposure is not the same across countries. While the identified interventions drastically reduce dust levels, workers may still be overexposed to RCS even under high-efficiency levels.

The review found that education, training, and marketing strategies improve respirator use, while training and education motivate workers to use dust control measures. Results from the review suggest that adopted interventions increase knowledge, awareness, and attitudes about respirator usage and generate positive perceptions about respirator usage while reducing misconceptions. Interventions can increase the use, proper use, and frequency of use of respirators and the adoption readiness for dust controls but may not provide sustained motivation in workers for the continual use of dust controls or PPEs.

There are several limitations to consider when interpreting the results of the review. Firstly, the individual studies controlled for different types and sizes of dust and dust monitoring tools and methods varied. Sampling was also done at different positions in various workplaces, which could have affected the reported results. Secondly, some dust measurements were time-weighted averages, while others were not, meaning that two studies reporting the same dust concentration could have exposed workers differently. Also, the study excluded non-peer-reviewed articles, publications before 2010, and articles not in English, which could have impacted the review. Finally, most RPE improvement studies relied on self-reported data, which may not accurately reflect the situation.

## Supplementary Information

Below is the link to the electronic supplementary material.

Supplementary file1 (DOCX 42 KB)

Supplementary file2 (DOCX 269 KB)

Supplementary file3 (DOCX 193 KB)

Supplementary file4 (DOCX 188 KB)

## Data Availability

Data will be made available on request.
