# Supplementary material for: A systematic review of the effectiveness of dust control measures adopted to reduce workplace exposure
Source: Environ Sci Pollut Res Int. 2023 Mar 25;30(19):54407–28. doi: 10.1007/s11356-023-26321-w (PMC10121514; doi:10.1007/s11356-023-26321-w)
Supplement: Supplementary file 1 — Supplementary file1 (DOCX 42 KB) [file 11356_2023_26321_MOESM1_ESM.docx]

**Supplementary Sheet 1**

A systematic review of the effectiveness of dust control measures adopted to reduce respirable crystalline silica exposure in the workplace

**Search Strategy**

Table 1 shows the search syntax for the study. The search syntax was slightly modified for each database due to word limitations and search configurations.

**Table 1**

| **Item** | **Search Syntax** |
| --- | --- |
| 1 | “Respirable crystalline silica” OR RCS OR dust OR “Respirable silica” OR "coal dust" |
| 2 | "dust exposure" OR "silica exposure" OR "personal exposure" OR "respirable crystalline silica exposure" OR "dust concentration*" OR "worker exposure" OR “exposure rate" OR "occupational exposure" OR "RCS exposure" |
| 3 | Occupation* OR construct* OR min* OR tunnel* OR underground OR "artificial stone" OR farm* OR quar* OR demolition OR "stone cutting" OR fracking OR blast* OR foundr* OR industr* |
| 4 | “silica dust control measure*” OR "dust suppression" OR “dust prevention” OR “dust control” OR “dust reduction*” OR wet* OR “local exhaust ventilation” OR “air curtain” OR “dust dispersion” OR “Water mist” OR foam OR “dust removal” OR "water spray" OR surfactant OR “silica exposure control” OR Intervention OR initiative OR program* OR “personal protective equipment” |
| 5 | “personal protective equipment” OR “personal protective wear” OR “respiratory program” OR respirator OR RPP OR PPE |
| 6 | Persua* OR Enhance OR improve OR motivate OR encourage OR complian* |
| 7 | Barrier* OR complian* OR challenge* OR “non-complian*” |
| 8 | “cluster randomized trial” OR “randomised controlled trial” OR “pre and post stud*” OR “evaluation stud*” OR “experimental design” OR “case series” OR “case stud*” OR “interrupted time-series” OR “case control” OR “cohort stud*” |

1. **EBSCOhost**

EBSCOhost returned 150,388 citations from the search sets A, B, C and D and were exported to the endnote library.

| **Date** | **Set** | **Search string** | **Search Type** | **Search Settings/Filters** |
| --- | --- | --- | --- | --- |
| 29/08/20 | 1 | “Respirable crystalline silica” OR RCS OR dust OR “Respirable silica” OR "coal dust" | Advance | Databases; GreenFILE, MasterFILE Complete, APA PsycBooks, UOW Catalogue, Academic Search Complete, CINAHL Plus with Full Text, Environment Complete, MEDLINE with Full Text, APA PsycInfo, Humanities International Complete, SocINDEX with Full Text, APA PsycArticles, ERIC, OpenDissertations, MEDLINE, Inspec, MasterFILE Reference eBook Collection Apply related words: YES Also search within the full text of the articles: YES Apply equivalent subjects: YES Search Mode: Boolean/Phrase |
| 29/08/20 | 2 | "dust exposure" OR "silica exposure" OR "personal exposure" OR "respirable crystalline silica exposure" OR "dust concentration*" OR "worker exposure" OR "occupational exposure" OR "RCS exposure" | Advance | Databases; GreenFILE, MasterFILE Complete, APA PsycBooks, UOW Catalogue, Academic Search Complete, CINAHL Plus with Full Text, Environment Complete, MEDLINE with Full Text, APA PsycInfo, Humanities International Complete, SocINDEX with Full Text, APA PsycArticles, ERIC, OpenDissertations, MEDLINE, Inspec, MasterFILE Reference eBook Collection Apply related words: YES Also search within the full text of the articles: YES Apply equivalent subjects: YES Search Mode: Boolean/Phrase |
| 29/08/20 | 3 | Occupation* OR construct* OR min* OR tunnel* OR underground OR "artificial stone" OR farm* OR quar* OR demolition OR "stone cutting" OR fracking OR blast* OR foundr* OR industr* | Advance | Databases; GreenFILE, MasterFILE Complete, APA PsycBooks, eBook Collection (EBSCOhost), UOW Catalogue, Academic Search Complete, CINAHL Plus with Full Text, Environment Complete, MEDLINE with Full Text, APA PsycInfo, Humanities International Complete, SocINDEX with Full Text, APA PsycArticles, ERIC, OpenDissertations, MEDLINE, Inspec, MasterFILE Reference eBook Collection Apply related words: YES Also search within the full text of the articles: YES Apply equivalent subjects: YES Search Mode: Boolean/Phrase |
| 29/08/20 | 4 | “silica dust control measure*” OR "dust suppression" OR “dust prevention” OR “dust control” OR “dust reduction*” OR wet* OR “local exhaust ventilation” OR “air curtain” OR “dust dispersion” OR “Water mist” OR foam OR “dust removal” OR "water spray" OR surfactant OR “silica exposure control” OR Intervention OR initiative OR program* OR “personal protective equipment” | Advance | Databases; GreenFILE, MasterFILE Complete, APA PsycBooks, UOW Catalogue, Academic Search Complete, CINAHL Plus with Full Text, Environment Complete, MEDLINE with Full Text, APA PsycInfo, Humanities International Complete, SocINDEX with Full Text, APA PsycArticles, ERIC, OpenDissertations, MEDLINE, Inspec, MasterFILE Reference eBook Collection Apply related words: YES Also search within the full text of the articles: YES Apply equivalent subjects: YES Search Mode: Boolean/Phrase |
| 29/08/20 | 5 | “personal protective equipment” OR “personal protective wear” OR “respiratory program” OR respirator OR RPP OR PPE | Advance | Databases; GreenFILE, MasterFILE Complete, APA PsycBooks, UOW Catalogue, Academic Search Complete, CINAHL Plus with Full Text, Environment Complete, MEDLINE with Full Text, APA PsycInfo, Humanities International Complete, SocINDEX with Full Text, APA PsycArticles, ERIC, OpenDissertations, MEDLINE, Inspec, MasterFILE Reference eBook Collection Apply related words: YES Also search within the full text of the articles: YES Apply equivalent subjects: YES Search Mode: Boolean/Phrase |
| 30/08/20 | 6 | Persua* OR Enhance OR improve OR motivate OR encourage OR complian* | Advance | Same as above |
| 30/08/20 | 7 | Barrier* OR complian* OR challenge* OR “non-complian*” | Advance | Same as above |
| 30/08/20 | 8 | “cluster randomized trial” OR “randomised controlled trial” OR “pre and post stud*” OR “evaluation stud*” OR “experimental design” OR “case series” OR “case stud*” OR “interrupted time-series” OR “case control” OR “cohort stud*” | Advance | Same as above |
| 30/08/20 | A | #1 AND #2 AND #3 AND #4 |  |  |
| 31/08/20 | B | #1 AND #4 AND #8 |  |  |
| 31/09/20 | C | #5 AND 6 |  |  |
| 31/09/20 | D | #5 AND #7 |  |  |

1. **PubMed**

The results set of 1, 2, 3, and 4 accounted for 14,694 citations and were exported to the endnote library.

| **Date** | **Set** | **Search string** | **Search Type** | **Search Settings/Filters** |
| --- | --- | --- | --- | --- |
| 31/08/20 | 1 | (("Respirable crystalline silica" OR RCS OR dust OR "Respirable silica" OR "coal dust") AND ("dust suppression" OR "dust prevention" OR "dust control" OR "dust reduction*" OR wetting OR "local exhaust ventilation" OR "air curtain" OR "dust dispersion" OR "Water mist" OR foam OR "dust removal" OR "water spray" OR surfactant OR "exposure control" OR Intervention OR initiative OR program* OR "personal protective equipment" OR "silica control")) AND ("cluster randomized trial" OR "randomised controlled trial" OR "pre and post stud*" OR "evaluation stud*" OR "experimental design" OR "case series" OR "case stud*" OR "interrupted time-series" OR "case control" OR "cohort studies") | Advanced | Search: All fields Search Year: 1971 to 2020 |
| 31/08/20 | 2 | ("personal protective equipment" OR "personal protective wear" OR "respiratory program" OR respirator OR RPP OR PPE) AND (Persua* OR Enhance OR improve OR motivate OR encourage OR complian*) | Advanced | Search: All fields Search Year: 1950 to 2021 |
| 31/08/20 | 3 | ("personal protective equipment" OR "personal protective wear" OR "respiratory program" OR respirator OR RPP OR PPE) AND (Barrier* OR complian* OR challenge* OR "non-complian*") | Advanced | Search: All fields Search Year: 1951 to 2021 |
| 1/09/20 | 4 | ((("Respirable crystalline silica" OR RCS OR dust OR "Respirable silica" OR "coal dust") AND ("dust exposure" OR "silica exposure" OR "personal exposure" OR "respirable crystalline silica exposure" OR "dust concentration*" OR "worker exposure" OR "exposure rate" OR "occupational exposure" OR "RCS exposure")) AND (Occupation* OR construct* OR mine OR mining OR tunnel* OR underground OR "artificial stone" OR farm* OR quar* OR demolition OR "stone cutting" OR tracking OR blast* OR foundr* OR industr*)) AND ("dust suppression" OR "dust prevention" OR "dust control" OR "dust reduction*" OR wetting OR "local exhaust ventilation" OR "air curtain" OR "dust dispersion" OR "Water mist" OR foam OR "dust removal" OR "water spray" OR surfactant OR "exposure control" OR Intervention OR initiative OR program* OR "personal protective equipment" OR "silica control") | Advanced | Search: All fields Search Year: 1955 to 2020 |

1. **Cochrane Library**

Search results of A, B, C and D were exported to the endnote library (6801 citations).

| **Date** | **Set** | **Search string** | **Search Type** | **Search Settings/Filters** |
| --- | --- | --- | --- | --- |
| 02/09/20 | 1 | “Respirable crystalline silica” OR RCS OR dust OR “Respirable silica” OR "coal dust" | Advanced (Search Manager) | Content: in Cochrane Reviews, Cochrane Protocols, Trials and Clinical Answers  (Word variations have been searched) Publication Date: All dates |
| 02/09/20 | 2 | "dust exposure" OR "silica exposure" OR "personal exposure" OR "respirable crystalline silica exposure" OR "dust concentration*" OR "worker exposure" OR "occupational exposure" OR "RCS exposure" | Advanced (Search Manager) | Content: in Cochrane Reviews, Cochrane Protocols, Trials and Clinical Answers  (Word variations have been searched) Publication Date: All dates |
| 02/09/20 | 3 | Occupation* OR construct* OR min* OR tunnel* OR underground OR "artificial stone" OR farm* OR quar* OR demolition OR "stone cutting" OR fracking OR blast* OR foundr* OR industr* | Advanced (Search Manager) | Content: in Cochrane Reviews, Cochrane Protocols, Trials and Clinical Answers  (Word variations have been searched) Publication Date: All dates |
| 02/09/20 | 4 | “silica dust control measure*” OR "dust suppression" OR “dust prevention” OR “dust control” OR “dust reduction*” OR wet* OR “local exhaust ventilation” OR “air curtain” OR “dust dispersion” OR “Water mist” OR foam OR “dust removal” OR "water spray" OR surfactant OR “silica exposure control” OR Intervention OR initiative OR program* OR “personal protective equipment” | Advanced (Search Manager) | Content: in Cochrane Reviews, Cochrane Protocols, Trials and Clinical Answers  (Word variations have been searched) Publication Date: All dates |
| 02/09/20 | 5 | “personal protective equipment” OR “personal protective wear” OR “respiratory program” OR respirator OR RPP OR PPE | Advanced (Search Manager) | Content: in Cochrane Reviews, Cochrane Protocols, Trials and Clinical Answers  (Word variations have been searched) Publication Date: All dates |
| 02/09/20 | 6 | Persua* OR Enhance OR improve OR motivate OR encourage OR complian* | Advanced (Search Manager) | Content: in Cochrane Reviews, Cochrane Protocols, Trials and Clinical Answers  (Word variations have been searched) Publication Date: All dates |
| 02/09/20 | 7 | Barrier* OR complian* OR challenge* OR “non-complian*” | Advanced (Search Manager) | Content: in Cochrane Reviews, Cochrane Protocols, Trials and Clinical Answers  (Word variations have been searched) Publication Date: All dates |
| 02/09/20 | 8 | “cluster randomized trial” OR “randomised controlled trial” OR “pre and post stud*” OR “evaluation stud*” OR “experimental design” OR “case series” OR “case stud*” OR “interrupted time-series” OR “case control” OR “cohort stud*” | Advanced (Search Manager) | Content: in Cochrane Reviews, Cochrane Protocols, Trials and Clinical Answers  (Word variations have been searched) Publication Date: All dates |
| 02/09/20 | A | #1 AND #2 AND #3 AND #4 | Advanced (Search Manager) | Content: in Cochrane Reviews, Cochrane Protocols, Trials and Clinical Answers  (Word variations have been searched) Publication Date: All dates |
| 02/09/20 | B | #1 AND #4 AND #8 | Advanced (Search Manager) | Content: in Cochrane Reviews, Cochrane Protocols, Trials and Clinical Answers  (Word variations have been searched) Publication Date: All dates |
| 02/09/20 | C | #5 AND #6 | Advanced (Search Manager) | Content: in Cochrane Reviews, Cochrane Protocols, Trials and Clinical Answers  (Word variations have been searched) Publication Date: All dates |
| 02/09/20 | D | #5 AND #7 | Advanced (Search Manager) | Content: in Cochrane Reviews, Cochrane Protocols, Trials and Clinical Answers  (Word variations have been searched) Publication Date: All dates |

1. **Web of Science**

Search results of A, B, C, D, and F were exported to the endnote library. A total of 8748 citations were exported.

| **Date** | **Set** | **Search string** | **Search Type** | **Search Settings/Filters** |
| --- | --- | --- | --- | --- |
| 31/08/20 | 1 | TS=(“Respirable crystalline silica” OR RCS OR dust OR “Respirable silica” OR "coal dust") | Advance | Databases= WOS, CSCD, CCC, KJD, MEDLINE, RSCI, SCIELO  Timespan=All years Search language=Auto Auto-suggest publication names=On |
| 31/08/20 | 2 | TS=("dust exposure" OR "silica exposure" OR "personal exposure" OR "respirable crystalline silica exposure" OR "dust concentration*" OR "worker exposure" OR “exposure rate" OR "occupational exposure" OR "RCS exposure") | Advance | Databases= WOS, CSCD, CCC, KJD, MEDLINE, RSCI, SCIELO  Timespan=All years Search language=Auto Auto-suggest publication names=On |
| 31/08/20 | 3 | TS=(Occupation* OR construct* OR min* OR tunnel* OR underground OR "artificial stone" OR farm* OR quar* OR demolition OR "stone cutting" OR fracking OR blast* OR foundr* OR industr*) | Advance | Databases= WOS, CSCD, CCC, KJD, MEDLINE, RSCI, SCIELO  Timespan=All years Search language=Auto Auto-suggest publication names=On |
| 31/08/20 | 4 | TS=(“silica dust control measure*” OR "dust suppression" OR “dust prevention” OR “dust control” OR “dust reduction*” OR wet* OR “local exhaust ventilation” OR “air curtain” OR “dust dispersion” OR “Water mist” OR foam OR “dust removal” OR "water spray" OR surfactant OR “silica exposure control” OR Intervention OR initiative OR program* OR “personal protective equipment”) | Advance | Databases= WOS, CSCD, CCC, KJD, MEDLINE, RSCI, SCIELO  Timespan=All years Search language=Auto Auto-suggest publication names=On |
| 31/08/20 | 5 | TS=(“personal protective equipment” OR “personal protective wear” OR “respiratory program” OR respirator OR RPP OR PPE) | Advance | Databases= WOS, CSCD, CCC, KJD, MEDLINE, RSCI, SCIELO  Timespan=All years Search language=Auto Auto-suggest publication names=On |
| 31/08/20 | 6 | TS=( Persua* OR Enhance OR improve OR motivate OR encourage OR complian*) | Advance | Databases= WOS, CSCD, CCC, KJD, MEDLINE, RSCI, SCIELO  Timespan=All years Search language=Auto Auto-suggest publication names=On |
| 31/08/20 | 7 | TS=(Barrier* OR  complian*  OR  challenge*  OR  “non-complian*”) | Advance | Databases= WOS, CSCD, CCC, KJD, MEDLINE, RSCI, SCIELO  Timespan=All years Search language=Auto Auto-suggest publication names=On |
| 31/08/20 | 8 | TS=(“cluster randomized  trial”  OR  “randomised  controlled  trial”  OR  “pre  and  post  stud*”  OR  “evaluation  stud*”  OR  “experimental  design”  OR  “case  series”  OR  “case  stud*”  OR  “interrupted  time-series”  OR  “case  control”  OR  “cohort  studies”  ) | Advance | Databases= WOS, CSCD, CCC, KJD, MEDLINE, RSCI, SCIELO  Timespan=All years Search language=Auto Auto-suggest publication names=On |
| 31/08/20 | A | #1 AND #2 AND #3 AND #4 | - | Databases= WOS, CSCD, CCC, KJD, MEDLINE, RSCI, SCIELO  Timespan=All years Search language=Auto Auto-suggest publication names=On |
| 31/08/20 | B | #1 AND #4 AND #8 | - | Databases= WOS, CSCD, CCC, KJD, MEDLINE, RSCI, SCIELO  Timespan=All years Search language=Auto Auto-suggest publication names=On |
| 31/08/20 | C | #5 AND #6 | - | Databases= WOS, CSCD, CCC, KJD, MEDLINE, RSCI, SCIELO  Timespan=All years Search language=Auto Auto-suggest publication names=On |
| 31/08/20 | D | #5 AND #7 | - | Databases= WOS, CSCD, CCC, KJD, MEDLINE, RSCI, SCIELO  Timespan=All years Search language=Auto Auto-suggest publication names=On |
| 14/10/20 | F | “dust control" | - | Databases= WOS, CSCD, CCC, KJD, MEDLINE, RSCI, SCIELO  Timespan=All years Search language=Auto Auto-suggest publication names=On |

1. **Proquest**

8568 citations from sets 1, 2, 3 and 4 were exported.

| **Date** | **Set** | **Search string** | **Search Type** | **Search Settings/Filters** |
| --- | --- | --- | --- | --- |
| 05/09/20 | 1 | ab("Respirable crystalline silica" OR RCS OR dust OR "Respirable silica" OR "coal dust") AND ab("dust exposure" OR "silica exposure" OR "personal exposure" OR "respirable crystalline silica exposure" OR "dust concentration*" OR "worker exposure" OR "exposure rate" OR "occupational exposure" OR "RCS exposure") AND ab(Occupation* OR construct* OR min* OR tunnel* OR underground OR "artificial stone" OR farm* OR quar* OR demolition OR "stone cutting" OR fracking OR blast* OR foundr* OR industr*) AND ab("silica dust control measure*" OR "dust suppression" OR "dust prevention" OR "dust control" OR "dust reduction*" OR wet* OR "local exhaust ventilation" OR "air curtain" OR "dust dispersion" OR "Water mist" OR foam OR "dust removal" OR "water spray" OR surfactant OR "silica exposure control" OR Intervention OR initiative OR program* OR "personal protective equipment") | Advanced Search | Source type: All Document: All Language: All Database: Ebook Central, ERIC, ProQuest Central, ProQuest Dissertations $ Theses A&I, Technology Collection |
| 07/09/20 | 2 | ab("Respirable crystalline silica" OR RCS OR dust OR "Respirable silica" OR "coal dust") AND ab(“silica dust control measure*” OR "dust suppression" OR “dust prevention” OR “dust control” OR “dust reduction*” OR wet* OR “local exhaust ventilation” OR “air curtain” OR “dust dispersion” OR “Water mist” OR foam OR “dust removal” OR "water spray" OR surfactant OR “silica exposure control” OR Intervention OR initiative OR program* OR “personal protective equipment”) AND ab(“cluster randomized trial” OR “randomised controlled trial” OR “pre and post stud*” OR “evaluation stud*” OR “experimental design” OR “case series” OR “case stud*” OR “interrupted time-series” OR “case control” OR “cohort stud*” ) | Advanced Search | Source type: All Document: All Language: All Database: Ebook Central, ERIC, ProQuest Central, ProQuest Dissertations $ Theses A&I, Technology Collection |
| 13/09/20 | 3 | ab(“personal protective equipment” OR “personal protective wear” OR “respiratory program” OR respirator OR RPP OR PPE) AND ab(Persua* OR Enhance OR improve OR motivate OR encourage OR complian*) | Advanced Search | Source type: All Document: All Language: All Database: Ebook Central, ERIC, ProQuest Central, ProQuest Dissertations $ Theses A&I, Technology Collection |
| 14/09/20 | 4 | ab("personal protective equipment" OR "personal protective wear" OR "respiratory program" OR respirator OR RPP OR PPE) AND (Barrier* OR complian* OR challenge* OR "non-complian*") | Advanced Search | Source type: All Document: All Language: All Database: Ebook Central, ERIC, ProQuest Central, ProQuest Dissertations $ Theses A&I, Technology Collection |

1. **Google Scholar**

Google Scholar returned 48,273 citations for the study.

| **Date** | **Resource** | **Search string** | **Search Settings/Filters** |
| --- | --- | --- | --- |
| 15/09/20 | Advanced Google Scholar | PPE "personal protective equipment" OR “improve use” "improve compliance" | with all of the words; PPE with the exact phrase; improve compliance with at least one of the words: "personal protective equipment" "improve use" where my words occur: any where in the article |
| 16/09/20 | Advanced Google Scholar | respirator PPE "personal protective equipment" "non compliance" "Barrier" "challenge" | with all of the words; respirator with the exact phrase; "personal protective equipment" "non compliance" "Barrier" "challenge" with at least one of the words: PPE where my words occur: any where in the article |
| 16/09/20 | Advanced Google Scholar | "dust control" | with the exact phrase; dust control where my words occur: any where in the article |
| 06/10/20 | Advanced Google Scholar | Intervention, respirable crystalline silica, exposure, industry tunnel, mine, initiative, program, control | with all of the words; intervention with the exact phrase; respirable crystalline silica with at least one of the words: exposure industry tunnel mine initiative program control without the words where my words occur: anywhere in the article |
| 07/10/20 | Advanced Google Scholar | control, respirable crystalline silica | with all of the words; control with the exact phrase; respirable crystalline silica where my words occur: anywhere in the article |
| 07/10/20 | Advanced Google Scholar | prevention, respirable silica | with all of the words; prevention with the exact phrase; respirable silica where my words occur: anywhere in the article |

1. **Scopus**

Search set A, B, C, D and E returned 82, 970 citations from A, B, C, D and E

| **Date** | **Set** | **Search string** | **Search Type** | **Search Settings/Filters** |
| --- | --- | --- | --- | --- |
| 9/10/20 | 1 | ALL ( "Respirable crystalline silica" OR rcs OR dust OR "Respirable silica" OR "coal dust" ) | Advanced Search | Search in Tittles, Abstracts and keywords |
| 10/10/20 | 2 | ALL ( "dust exposure" OR "silica exposure" OR "personal exposure" OR "respirable crystalline silica exposure" OR "dust concentration*" OR "worker exposure" OR "exposure rate" OR "occupational exposure" OR "RCS exposure" ) | Advanced Search | Search in Tittles, Abstracts and keywords |
| 10/10/20 | 3 | ALL ( occupation* OR construct* OR min* OR tunnel* OR underground OR "artificial stone" OR farm* OR quar* OR demolition OR "stone cutting" OR fracking OR blast* OR foundr* OR industr* ) | Advanced Search | Search in Tittles, Abstracts and keywords |
| 11/10/20 | 4 | ALL ( "silica dust control measure*" OR "dust suppression" OR "dust prevention" OR "dust control" OR "dust reduction*" OR wet* OR "local exhaust ventilation" OR "air curtain" OR "dust dispersion" OR "Water mist" OR foam OR "dust removal" OR "water spray" OR surfactant OR "silica exposure control" OR intervention OR initiative OR program* OR "personal protective equipment" ) | Advanced Search | Search in Tittles, Abstracts and keywords |
| 11/10/20 | 5 | ALL ( "personal protective equipment" OR "personal protective wear" OR "respiratory program" OR respirator OR rpp OR ppe ) | Advanced Search | Search in Tittles, Abstracts and keywords |
| 11/10/20 | 6 | ALL ( persua* OR enhance OR improve OR motivate OR encourage OR complian* ) | Advanced Search | Search in Tittles, Abstracts and keywords |
| 11/10/20 | 7 | ALL ( barrier* OR complian* OR challenge* OR "non-complian*" ) | Advanced Search | Search in Tittles, Abstracts and keywords |
| 11/10/20 | 8 | ALL ( "cluster randomized trial" OR "randomised controlled trial" OR "pre and post stud*" OR "evaluation stud*" OR "experimental design" OR "case series" OR "case stud*" OR "interrupted time-series" OR "case control" OR "cohort stud*" ) | Advanced Search | Search in Tittles, Abstracts and keywords |
| 13/10/20 | A | #1 AND #2 AND #3 AND #4 |  |  |
| 13/10/20 | B | #1 AND #4 AND #8 |  |  |
| 13/10/20 | C | #5 AND #6 |  |  |
| 13/10/20 | D | #5 AND #7 |  |  |
| 14/10/20 | E | dust control | Advanced Search | Search in Tittles, Abstracts and keywords |
