# Supplementary material for: A systematic review of the effectiveness of dust control measures adopted to reduce workplace exposure
Source: Environ Sci Pollut Res Int. 2023 Mar 25;30(19):54407–28. doi: 10.1007/s11356-023-26321-w (PMC10121514; doi:10.1007/s11356-023-26321-w)
Supplement: Supplementary file 2 — Supplementary file2 (DOCX 269 KB) [file 11356_2023_26321_MOESM2_ESM.docx]

**Supplementary Sheet 2 (2a - page 1, 2b - page 31 and 2c - page 34)**

A systematic review of the effectiveness of dust control measures adopted to reduce respirable crystalline silica exposure in the workplace.

NB: *Total dust control efficiency is TDCE, and Respirable dust control efficiency is RDCE*

*Respirable dust refers to the portion of dust particles that can penetrate the airways and reach the lungs. Total dust refers to all dust particles contained in a volume of air.*

***Table 1. Dust control studies***

| **Title** | **Country of Study** | **Study Design** | **Industry - Workplace** | **Aim of the study** | **Intervention Measures** | **Rating of Effectiveness** | **Outcome Measures** |
| --- | --- | --- | --- | --- | --- | --- | --- |
| (Zhou, W et al. 2020) | China | Before and After Study | Construction - Tunnelling | To optimize the parameters of a dust removal ventilation system. | Far-pressing-near-absorption (FPNA) ventilation system with optimised pressurised air flow rate (${6m}^{3}/s$), air extraction flow rate (${10m}^{3}/s$) and diameter of the pressurised air duct (0.85m).  Compared system has a pressurised air flow rate (${10m}^{3}/s$) and air extraction flow rates (${8m}^{3}/s$ ). | Dust fall rate greater than 80.35%. | Dust concentration   Dust control distance |
| (Hu et al. 2020) | China | Quasi-experimental | Construction site | To evaluate the performance of a starch-based dust suppressant. | Liquid starch-based dust suppressant (LSDS) prepared by grafting polyacrylic acid onto a sodium hydroxide pre-treated potato starch. The LSDS consist of 7.5wt% gelatine and 7.5wt% glycerine. A mixture of 0.25wt% LSDS and water at ${10kg/m}^{2}$. Compared with water misting intervention. | TDCE for ${PM}_{2.5}$ is 57%.  TDCE of LSDS is 25% more than water misting. | Reduction in dust concentration |
| (Kokkonen et al. 2017) | Finland | Quasi-experimental | Construction - Renovation site | To evaluate the impact of partitioning and negative pressure applied in a renovated room. | Negative pressure was created between the renovation site and adjacent areas. Portable exhaust fans were installed in the partitioned renovation sites. Exhaust air was filtered and led outdoors. Partitioning was achieved using existing walls and room division or erecting temporary wall structures.  Indoor air quality was compared to the adjacent environment. | Partitioning and negative pressurization practices does not contain dust adequately. | Reduction in dust concentration |
| (Kokkonen et al. 2019) | Finland | Quasi-experimental | Construction - Renovation site | To evaluate the impact of using local exhaust ventilation (LEV) in an enclosed room affects dust dispersing in adjacent areas. | LEVs with on-tool extraction, ventilation shroud, and the moveable capturing hood were used in negatively partitioned renovation rooms.  Compared with dust in adjust rooms. | RDCE is up to 90% in the renovation site and 87% for the intervention. | Reduction in dust concentration |
| (He et al. 2018) | China | Quasi-experimental | Construction - Tunnelling as part of hydropower station construction | To evaluate the effect of a reverse circulation (RC) drilling system used in underground tunnel drilling. | RC air hammer drilling system with double-row suction nozzles uses the supplied compress air for drilling to carry rock cutting upward through the central passage of the drill. The dust is collected and discharged.  Compared with conventional drilling technology. | No visible dust or rock cutting suspended in the underground space. | Improved visibility |
| (Echt et al. 2016) | USA | Before and After Study | Construction - Outdoor testing area | To evaluate the dust control impact of an LEV when using a concrete dowel drilling machinery. | LEV system installed on the dowel drill with hood positioned to create a temporary enclosure. Dust is captured and filtered from the airstream.  Compared with drilling with no LEV. | The intervention reduced emissions over 90%. | Reduction in dust concentration |
| (Summers & Parmigiani 2015) | USA | Quasi-experimental | Construction - Field-laboratory experiment | To evaluate the effectiveness of a surfactant used chainsaw concrete cutting. | A surfactant additive consisting of 40% Sodium C14-16 alpha-olefin sulfonate and 60% water.  Series I – 2.0% surfactant in the spray mixture.  Series II - 0.2% surfactant in the spray mixture.  Series II had the surfactant-water mixture drawn from the same Reservoir.  Compared with OSHA permissible exposure limit (PEL). | TWA below ${5mg}/{m^{3}}$. | Reduction in dust concentration |
| (Garcia et al. 2014) | USA | Quasi-experimental | Construction | To evaluate the effectiveness of aftermarket LEVs on powered saw for cutting concrete roofing tiles. | The LEV was fixed on the worm-driven electric circular saw with the shroud connected to an electric axial fan to collect dust at the point of generation. Extracted dust is bagged and disposed.  Compared to OSHA PEL, NIOSH REL and ACGIH TLV. | Average 8-hour TWA for respirable crystalline dust (RCS) was ${0.28mg}/{m^{3}}$. | Reduction in dust exposure |
| (Shepherd & Woskie 2013) | USA | Quasi-experimental | Construction - Field-laboratory experiment/Outdoors | To evaluate the effectiveness of a water-based dust control on hand-held concrete saw. | Wetting and spraying with water for the saw from the main water hose (wet) or a portable pressurized canister (spray). Water supply was attached through the connector on the blade guard housing on the saw.  Compared with dry cutting. | RCS reduction rate with wet control is 90.44%. RCS reduction rate with water spray is 92.36%.  Water controls may not reduce levels to below OSHA PEL. | Reduction in dust concentration |
| (Wallace & Cheung 2013) | UK | Quasi-experimental | Construction - Demolition | To demonstrate the benefits of atomised water dust control during demolition. | A water misting system was installed on an excavator-mounted hydraulic breaker. Water storage tank, compressor and water pump were installed on the excavator with a nozzle mounted on the hydraulic breaker. Optimal control method was a 45° nozzle at a water flow rate of 44L/h.  Compared with 45° nozzle with 22L/h flow rate, a 60° nozzle at 22L/h and 44L/h flow rate and conventional dust suppression. | RDCE for 45° nozzle with 44L/h is up to 94.1%.  RDCE for 45° nozzle with 22L/h is up to 36.8%.  RDCE for 60° nozzle with 44L/h is 88.6%.  RDCE for 60° nozzle with 22L/h is 56.5%.  RDCE for convention dust control is up to 89.5%. | Reduction in dust concentration |
| (Middaugh et al. 2012) | USA | Quasi-experimental | Construction site | To evaluate the effectiveness wet suppression and LEV for commercial cut-off saws. | The wet dust control was integrated in the cut-off saw by the manufacturer. It had spray nozzle on each side of the saw guard spraying water on the cutting blade.  The LEV saw consists of a spring-loaded, movable guard that diverts dust into a belt-driven fan system below the saw. Extracted dust is bagged and disposed.  Compared with ACGIH TLV. | RDCE for wet suppression is 78.0%. RDCE for LEV is 73%.  Wet suppression and LEV exceed the ACGIH TLV by 8.4 times and 13.2 times, respectively. | Reduction in dust concentration |
| (Fan et al. 2012) | Hong Kong | Quasi-experimental | Construction - Field-laboratory experiment | Evaluated the effectives of the DustBubbles. | Disposable DustBubbles grip the area around industrial drills and catch dust during the drilling process.  Compared to drilling without DustBubbles. | RDCE of Dust Bubble is 63%. | Reduction in dust concentration |
| (Cooper et al. 2012) | USA | Quasi-experimental | Construction - Field-laboratory experiment | To evaluate the effectiveness of an LEV on a movable jig used for pneumatic drilling | An LEV was mounted on a movable jig and had a hood in the shape of a cylindrical shroud that surrounded the drill bit. The shroud was held in place with locks.  Compared with drilling without the jig. | RDCE for drilling with jig and dust control was 94.3%.  RDCE for drilling with jig without dust control was 55.3%. | Reduction in dust concentration |
| (Akbar-Khanzadeh et al. 2010) | USA | Quasi-experimental | Construction - Field-laboratory experiment | To evaluate the effectiveness of dust controls for manual concrete grinding | Industrial fan as general ventilation.  LEV1 was retrofitted to the angle grinders and used the HEPA-Cyclone dust control method.  LEV2a used the HEPA-Tank control method with the angle grinders retrofitted urethane dust shroud and attached Eibenstock tank vacuum with HEPA filters. Auto filter cleaning was utilised.  LEV2b, the outlet of the Eibenstock concrete grinder was attached to vacuum hosing to the Eibenstock tank vacuum with HEPA filters.  LEV3 - angle grinders were retrofitted with a heavy-duty urethane dust shroud and attached to a shop vacuum.  Wet grinding was retrofitted on each grinder. Hole created in the metal blade guards allowed water to flow through a hose and nipple to control dust at a regulated water flow rate.  Compared with conventional uncontrolled grinding. | RDCE for general ventilation is 66%.  RDCE for LEV: HEPA grinding is 99%.  RDCE for LEV: Shop-vac grinding is 98.1%.  RDCE for wet grinding is 94.4%. | Reduction in dust concentration |
| (Li, Y et al. 2020) | China | Before and After Study | Energy - Thermal power plant | To evaluate the effectiveness of dust control for a coal conveying process in power plant. | A dust cleaning device with a dust hood, contact scrubber, suction fan automatic sprinkler on belt, online dust concentration monitors and control system.  Water is sprayed on the absorbing material when the extracted air enters the scrubber. As the air ascends, the turbulent dewatering device removes the water in the air. | Dust collection efficiency was 99.04%.  ${PM}_{5}$ control efficiency was 67% | Reduction in dust concentration |
| (Morteza et al. 2013) | Iran | Before and After Study | Foundry | To evaluate the efficiency of an LEV in a foundry. | The LEV was designed to meet requirements of Method C of the ACGIH. The ventilator also met VS-85-10, VS-50-10, VS-99-01 and VS-50-21 standards.  Compared to NIOSH TLV. | Average crystalline silica control efficiency was 81.82% and lower than NIOSH TLV. | Reduction in dust concentration |
| (Lin et al. 2011) | Taiwan | Longitudinal study | Foundry | To evaluate the impact an engineered dust control intervention in an iron foundry plant. | The intervention was made up of two water misting systems and the use of industrial fans. One misting system was installed at the inlet/outlet point of the sand pouring out machine and the other was at the waste sand deposit area. They were made up of 100 nozzles, each 0.3mm in diameter, and a water flow rate of 100 mL per minute. Two wall-mounted industrial fans brough clean external air. | RDCE is 44.3%. | Worker’s lung function |
| (Kanjiyangat & Hareendran 2018) | India | Before and After Study | Coal fired boiler plant | To evaluate the effectiveness of a water misting system handling in a pulverized coal boiler environment. | The mist system operates at 68.95bar to generate 10 µm sized water droplets from 80 nozzles vertically mounted and spaced 0.5 m apart at a height of 3m. | A reduction of up to 81% in dust concentration. | Reduction in dust concentration |
| (Lin et al. 2014) | China | Before and After Study | Laboratory Work | To evaluate a designed sample preparation room dusting system. | The dedusting system had flat and external suction hood that collected dust from dust sources and passed the dusty air through the bag type dust filter. | Indoor dust control efficiency is 98.16%. | Reduction in dust concentration |
| (Zarei et al. 2018) | Iran | Before and After Study | Manufacturing - Tile manufacturing | To assess the effectiveness of an LEV system for decreasing dust and crystalline silica dust in the plant. | The LEV was designed to meet requirements of the ACGIH VS-50-21 standard. The source pollution, available spaces, physicochemical properties of the pollutant and the method of pollutant emission determined the design of the hood of equipment without VS. Dust air travels to the wet scrubbers. The slurry from the wet scrubber is reused. | TDCE is 66%. Inhalable dust reduction efficiency is 94%. Silica dust reduction efficiency is 96%. | Reduction in dust concentration |
| (Firdaussyah & Suryo 2018) | Indonesia | Cross Sectional Study | Manufacturing - Steel fabrication yard | To evaluate control methods for silica-quartz fraction exposure from almandine garnet as a sandblasting agent in steel construction companies. | LEV complement dust control efforts in the sandblasting room. Administrative controls like safe work procedures, job start meeting, sandblasting health and safety promotion activities and the provision of respiratory protection equipment (RPE).  Compared to Indonesian law regulating silica dust and other OELs like OSHA PEL ACGIH TLV. | ${>108.21mg}/{m^{3}}$ | Reduction in dust concentration |
| (Sun et al. 2019) | China | Quasi-experimental | Mining - Under the hydraulic support in a coal mining face | To evaluate the dust control effect of a novel Venturi negative-pressure secondary dedust device (VNPSDD) at a coal cutting face. | The VNPSDD consists of a tapered section cast with dual-anti ABS engineering plastic and a cross-cut hybrid solid-pyramid nozzle with an X-shaped diversion core and a spray orifice diameter of 1.9 mm. The high-speed water jet from the nozzle creates a negative pressure in the tapered section to inhale dust-laden air from the suction nozzle to mix with the spray. A local spray closure technique was formed with several VNPSDD by arranging them between the hydraulic supports.  Compared with the original dust suppression system. | RDCE was up to 80.7% at the shearer driver position.  Compared with the original dust suppression measures, respiratory dust efficiency improved by 44.3%. | Reduction in dust concentration |
| (Zhou, G et al. 2020) | China | Before and After Study | Mining - Coal Mining Face | To reduce advancing support dust in a coal mine. | The negatively pressured spraying collector consist of a tapered section and ultrasonic atomizing nozzle with a diameter of 2.0mm air pressures of 0.4 MPa. The high-speed water jet from the nozzle creates a negative pressure in the tapered section to inhale dust-laden air from the suction nozzle. The intervention was installed on the top beam of hydraulic support and was arranged along with the length and width directions of advancing support helping to suck dirty airflow in the sidewalk area. | Average TDCE is 81.5%. Average RDCE is 79.1%. | Reduction in dust concentration |
| (Louk et al. 2020) | USA | Quasi-experimental | Mining - Bagging and palleting in industrial sand mining operations | To evaluate the effectiveness of controls used in reducing workers’ exposures to respirable dust in industrial sand bagging and palletizing operations. | A dual nozzle bagging system made up of an inner fill nozzle combined with an outer exhaust nozzle. Operation 3 utilised the traditional fill nozzle without an exhausting component.  An overhead air supply island system that delivered an envelope of clean, filtered air to the bag operator.  A bag and belt cleaning device that cleaned dust from the bags as they travel from the bag loading station to the pelletizing station.  Use of LEV systems.  Plastic wrapping of the bags to prevent residual dust from escaping.  Compared to OSHA PEL, NIOSH and ACGIH RELs. | Average dust concentration in Operations 1, 3 and 4 is ${<100\mu g}/{m^{3}}$.  Average dust concentration in Operation 2 was ${<80\mu g}/{m^{3}}$. | Reduction in dust concentrations |
| (Ren et al. 2020) | China | Quasi-experimental | Mining - Coal mine roadway | To evaluate the effectiveness of a novel dust suppression device based on the principles of Coanda effect. | The intervention was designed with a 15º tapered inlet create negative pressure to draw dust. The inhaled air is sprayed with water to cause heavy particles to drop to the wall. The outlet is incline at 5º and has several filters. The optimal number of the nozzles was 36. | TDCE of the Coanda effect intervention was to 80.7%. TDCE of the original device was up to 59.1%.  RDCE of the Coanda effect intervention was up to 85.2%  RDCE of the original device was up to 32.2%. | Reduction in dust concentration |
| (Reed, Shahan, Ross, et al. 2020) | USA | Before and After Study | Mining - Coal Mining Areas | Tested the effectiveness of a wet dust collector for a roof bolter. | The wet collector was dry vacuum dust collector on roof bolters. A water spray nozzle in the dust box replaces the precleaner and cyclones. The wet material is emptied at the bottom of the box.  Compared with the dry box collector. | Dust reduction is the same except when cleaning the box.  Average dust reduction for cleaning the wet box collector compared with cleaning the dry box collector was about 60%. | Reduction in dust concentration |
| (Patts et al. 2020) | USA | Quasi-experimental | Metal and nonmetal mining | To identify and address specific task that led to  peak exposures. | A camera is used to video the worker's activities during personal exposure data collection. The data from the camera and sampler are evaluated with EVADE software to identify the work practices contributing to exposure. This allows the implementation of new controls or better utilisation of existing controls. | Exposures could be reduced by at least 20%. | Reduction in dust exposure |
| (Peng, Huitian et al. 2020) | China | Before and After Study | Mining - Coal mining face and roadway | To optimize a negative pressure mist-curtain dust suppression device. | The intervention is a three-sided arc-shaped tube installed around the heading machine’s cutting arm with spray nozzles facing the cutting face. The eight single-swirl atomising spray nozzles are surrounded by conical covers and directly connected to the suction hoods which face upwards. The suction effect of the device was further enhanced compared to the original instrument by modifying the positions and angles of the nozzle and the conical cover.  Compared original spray dust suppression device. | Compared with the original spray dust suppression device, the dust removal efficiency was increased by at least 3.73%.  Dust removal efficiency was 77.92%. | Reduction in dust concentration |
| (Li, G et al. 2020) | China | Before and After Study | Mining - Transfer station of a metal mine | To evaluate the effectiveness of a swirling curtain dust collector (SCDC). | Dusty air is drawn into the SCDC. The ultrasonic nebulizer produces a water curtain, and a pressure sprayer causes the dust in the air to form a slurry. The slurry is thrown into the cyclone's wall and removed under the action of the centrifugal force. The intervention was operated at: air pressure: 0.35 MPa, water volume: 30 L/h; liquid-gas ratio: 0.15 L/m3; wind speed: 14–16 m/s.  Compared with the venturi wet dust collector. | The dust removal efficiency was over 97%. | Reduction in dust concentration |
| (Liu et al. 2020) | China | Quasi-experimental | Mining - Coal mining face of a roadway | To improve the dust control in a coal mine tunnel. | The integrated vortex ventilation and dust removal system consist of a vortex ventilation device composed of a wall-attached duct, telescopic duct, and storage device. The wall-attached duct turns the air jet into axial rotating airflow. During dust removal the butterfly valve on the wall-attached duct closed, and the venting valve is opened. The airflow provided by the fan goes through the wall-attached duct and is ejected in the form of swirling airflow.  Compared to a FPNA ventilation. | RDCE of vortex ventilation is 80%. | Reduction in dust concentration |
| (Cheng et al. 2020) | China | Before and After Study | Mining - Coal transportation port | To investigate the coal dust control of a composite binder made from Sodium Dodecyl Dulfate (SDS). | The coal dust suppression system is made up of a composite binder spraying subsystem and wind prevention and dust suppression network subsystem. The composite binder: SDS had an optimal concentration of ${0.004g}/{{cm}^{3}-0.006g/{cm}^{3}}$ and could effectively wet dust of 60−80 mesh diameter. | Efficiency on day 15 after using the suppressant is 78%. | Reduction in dust concentration |
| (Cai et al. 2020) | China | Before and After Study | Mining - Coal Mining Face | To reduce the dust exposure at the shearer drivers working area. | Two extraction-type dust purifying fans are arranged symmetrically on the rocker arm of the shearer, with the fan's axis facing the roller directly. There is a ring of nozzles that spray the extracted dust with water before it exits. The nozzles  and fan are housed in drum-shaped casing. | TDCE at the shearer driver area 90.47%. | Reduction in dust concentration |
| (Wang et al. 2020) | China | Quasi-experimental | Mining - Coal mine roadway | Tested a foam-water mist integration technology. | The integrated foam-mist device has an air and water supply pipeline, switch valve set, function valve set, regulating valve set, foam generating device, integrated nozzle and foam conveying pipeline. The water supply and air supply pipelines have a diameter of 51 mm to export the foam. And regulated by the switch valve. The new foam mist has 6 flat fan foam nozzles, the injection parameters of a single nozzle were 26° angle of diffusion, and the effective injection length was 1.5 m. The compression angle and uniform angle of foam nozzle are determined to be 6° and, 25° respectively. | TDCE at the driver’s place is 93%, 75% and 94% when using foam-water mist integration technology,  water mist function only and the foam function only respectively.  RDCE is 95%, 71% and 93% when using foam-water mist integration technology, water mist function only and the foam function only respectively. | Reduction in dust concentration |
| (Reed et al. 2019) | USA | Quasi-experimental | Mining - Coal Mining Areas | To evaluate the effectiveness of the roof bolter canopy air curtain (CAC) for mine dust control. | The CAC system was integrated into the roof bolter machine and the plenum provided uniform airflow over the miner’s breathing zone. The 2nd generation had staggered perimeter nozzles to prevent infiltration of contaminated air into the protection zone and consisted of a pattern of holes providing lower-velocity uniform airflow over the roof bolter operator.  Compared to when not working under the CAC. | Dust control efficiencies was up to 91%. | Reduction in dust concentration |
| (Reed, Shahan, Klima, et al. 2020) | USA | Quasi-experimental | Mining - Coal Mining Areas | To evaluate the effectiveness of the 3rd generation roof bolter canopy air curtain (CAC) in mine operating conditions. | The intervention is a CAC system integrated into the roof bolter machine. The 3rd generation device consists of a plenum constructed of a single flat aluminium plate, smaller-diameter airflow openings, and a single row of perimeter nozzles. It blows uniform air over the miner’s breathing zone. | Efficiencies was up to 60% for the left roof bolter.  Efficiencies 47% for the right bolter operator. | Reduction in dust concentration |
| (Guo et al. 2020) | China | Quasi-experimental | Mining - Coal Mining Face | To improve the external spray system of a continuous miner. | The spray system uses a round-mouth nozzle with an X-type diversion core with a calibre of 2.4 mm at an inclination angle of 15 and a spray pressure of 7 MPa.  Compared to efficiencies at different spray pressures. | TDCE at 1 MPa reached 44.7%.  TDCE at 3 MPa reached 72.6%.  TDCE at 5 MPa reached 85.3%.  TDCE at 7 MPa reached 90.6%.  TDCE at 7 MPa reached 91.7%.  RDCE at 1 MPa reached 44.3%.  RDCE at 3 MPa reached 75.1%.  RDCE at 5 MPa reached 83.8%.  RDCE at 7 MPa reached 90.4%.  RDCE at 8 MPa reached 91.4%. | Reduction in dust concentration |
| (Zhu et al. 2020) | China | Quasi-experimental | Mining - Tunnelling Face | Verified the performance of a foam generating system for workplaces with low water inlet pressure and high ventilation volume. | The foam spraying intervention has a cavitating jet pump (CJP), vertical foam generator (VFG), and 3D printed nozzles. The flow of high-pressure water through the CJP automatically draws in foaming agent (1wt% DSFA) into the system. DSFA mixes with the air to form foam with foam expansion 28-30 and delivered to the 3D printed nozzles for spraying. The system operates with an airflow rate of ${{35m}^{3}}/h$, air pressure of 0.22MPa, water flow rate ${{1.5m}^{3}}/h$, water pressure of 0.87MPa and nozzle pressure of 0.13 MPa. The VFG prevents inadequate air and liquid mixing during foam generation.  Compared with water misting system. | Average TDCE for foam was 86.9%. Average TDCE for water misting was 42.8%.  Averaged RDCE for foam was 86%  Averaged RDCE for water was 23.5%. | Reduction in dust concentration |
| (Bao et al. 2020) | China | Quasi-experimental | Mining - Coal Mining Face | Evaluated a dust suppression gel prepared by using graft copolymerization of itaconic acid and acrylic acid. | The dust suppressant prepared by graft copolymerization of itaconic acid-acrylic acid polymer and bentonite. The dust suppression gel was used to spray dust in a coal mine.  Compared with water misting. | The dust control efficiency is more than 9 times that of spraying water. | Reduction in dust concentration |
| (Hua et al. 2020) | China | Quasi-experimental | Mining - Tunnelling Face | To evaluate the dust control performance of a novel multi-radial-vortex-based ventilation system. | The air curtain was formed with a multi-radial vortex airflow generator with a diameter of 0.6m, a length of 0.95m and an 0.75 inflow-to-outflow. The outlet is 20m from the tunnelling face. The exhaust and blower ducts have a 0.6m diameter and their inlets are 3m and 10m away from tunnelling face respectively. A KCS-300D-type wet dust-removal ventilation fan with an outflow of around ${325m^{3}}/{min}$ is used.  Compared to the use or non-use of the generator at an inflow-to-outflow ratio of 1.25. | Average dust suppression efficiency at 0.75 inflow-to-outflow was 82.93%.  Average dust suppression efficiency at 1.25 inflow-to-outflow was 70.84%. | Reduction in dust concentration  Dust diffusion distance |
| (Xu et al. 2020) | China | Quasi-experimental | Mining - Under the hydraulic support in a coal mining face | Analysed the impacts of spray pressure, nozzle radius, and suction port of spraying dust settling device on dust suppression under the hydraulic support in a coal mine. | The intervention is made up of a 2.4mm spray nozzle covered with an ejector tube. The tube has a ring attachment for hanging on the hydraulic support. As high-speed water droplets flow out of the nozzle, dust is absorbed from the suction port. Each hydraulic support has two of the devices mounted and operated at 8MPa.  Compared with a similar device with side suction ports, 2.4mm nozzle and 6 MPa spray pressure. | TDCE and RDCE for the intervention was 86.0% and 89.3% respectively.  TDCE and RDCE for the comparison was 76.4% and 72.9% respectively. | Reduction in dust concentration |
| (Peng, H. et al. 2020) | China | Quasi-experimental | Mining - Large coal mining driveway | To evaluate the dust control impact of a water curtain covering the full section of the roadway in coal mining face. | The water curtain was designed with a flat opening type II nozzle with a 2mm aperture inclined at 15° towards the upwind side. At a water spray pressure of 7MPa the water curtain covers 93.1% of the roadway. Three sets of water curtains were later adopted.  Compared with similar water curtain with nozzle inclined at 0° and different spray pressures. | The ${PM}_{2.5}$settling rate at 7 MPa and nozzle inclination angle of 15° was 95.7%. | Reduction in dust concentration |
| (Ma et al. 2020) | China | Quasi-experimental | Mining - Fully mechanised coal mine | To evaluate a disc-type spraying device with a large spray field. | The external spraying device is an atomizer mounted at the front end of the movable arm of the coal cutter. Four nozzles were uniformly distributed on the side of the atomizer. The optimal spraying pressure was 8 MPa. The nozzle caliber was 2.4 mm.  Compared to the external spraying device at 2 MPa, 4 MPa and 6 MPa spraying pressure. | TDCE was 90.33% at the position of the driver. | Reduction in dust concentration |
| (Guo et al. 2019) | China | Quasi-experimental | Mining - Coal mining face of the air return roadway | To evaluate the effectiveness of composite foaming agent in a tunnelling operation. | A composite foaming agent with a static contact angle between 19 - 26° and maximum viscosity of 751 mPa s at an expansion ratio of 30. The foaming agent is automatically drawn by a Venturi-type foam adding device. A rotatable nozzle made of steel with a 120° spray angle is used.  Compared with water misting. | At the driver’s position TDCE for foam was 87.9% and RDCE was 75.97%.  At the driver’s position TDCE for water misting was 41.88% and RDCE was 32.1%. | Reduction in dust concentration  Visibility in the work area |
| (Wang, X et al. 2019) | China | Quasi-experimental | Mining - Fully mechanised coal mining face | To evaluate the effectiveness of a compound surfactant that meets the MT 506–1996 national standard of China. | Compound surfactant of 0.025 wt% Fatty Acid Methyl Esters ethoxylate sulfonate (FMES) and 0.025 wt% Coconut diethanolamine solution (CDEA)  Compared with untreated water. | TDCE of the compound surfactant was 90.32%.  RDCE of the compound surfactant was 87.96%.  Improved dust suppression up to 48.81% when compared to untreated water. | Reduction in dust concentration |
| (Wang, K et al. 2019) | China | Quasi-experimental | Mining - Coal mine roadway | To evaluate the dust control ability of a compound wetting agent and a nozzle with secondary atomisation. | Wetting agent E was formed from several surfactants and was used with a circular nozzle. Wetting agent E had a water consumption rate of ${0.76m^{3}}/h$ and a contact angle of 14.4°. The low-speed ejector air of circular nozzle promotes secondary atomisation.  Compared with high pressure strays with wetting agent A, elliptical nozzles and water misting. | All measurements are from the driver’s position.  TDCE of wetting agent E with circular nozzles was 89%.  TDCE of wetting agent A with circular nozzles was 87%.  TDCE of water misting with circular nozzles was 70%.  RDCE of wetting agent E with circular nozzles was 86%.  RDCE of wetting agent A with circular nozzles was 82%.  RDCE of water misting with circular nozzles was 58%. | Reduction in dust concentration |
| (Peng, Huitian et al. 2019) | China | Quasi-experimental | Mining - Coal Mining Face | To improve the efficiency of a shearer’s external spray device. | The ${PM}_{10}$ wet type air assisted spraying device is a 400mm diameter stainless-steel drum-shaped casing with an installed explosion-proof waterproof fan. Ten nozzles are installed in a ring shape at the air outlet. The nozzles have a 91.6° spray angle, 4.35 L/ min flow rate and uses a No. III’s spraying field with 2.01m spray range. | The dust control rate was up to 91.5%. | Reduction in ${PM}_{10}$ concentrations |
| (Peng, H. et al. 2019) | China | Before and After Study | Mining - Under the hydraulic support in a coal mining face | To test the effectiveness of a wind-assisted centralised hydraulic support spraying dedusting device for dust control under. | The device was a three-sided cover over a spraying jet, negative-pressure pneumatic motor with impellers around the air suction inlet. The air-induced cover around the spraying jet is set at angle of 45° to guide the airflow downwards. The spraying jet was formed by three nozzles and mist were assisted by high-velocity airflow from the motor. At a spray pressure of 8 MPa and an air pressure of 1.0 MPa. | Dust suppression rate up to 87.96%. | Reduction in dust concentration |
| (Lu et al. 2019) | China | Quasi-experimental | Mining - Coal mining face | To test a parallel jet adding device (PJAD) for foam dust suppression in a coal mine. | The PJAD automatically adds the foaming agent as pressure water flows through it. It generates foam with a foam expansion ratio of 24 – 27 and a foam spraying distance of 2.2 – 3.4 m.  Compared to water misting. | TDCE for foam was 85.7%. TDCE for water misting was 40.2%.  RDCE for foam was 87.7%. RDCE for water misting was 35.6%. | Reduction in dust concentration |
| (Li, S et al. 2019) | China | Quasi-experimental | Mining - Coal mine roadway | To reduce dust in a narrow coal mine roadway using a dry-type filtration dust collector. | The dry-type filtration dust collector was part of a FPNA ventilation system and had filtration system, a pulse jet cleaning system and an extraction fan. The forced air duct and exhaust ducts were 13m and 3m from the heading face respectively. The forced air duct and exhaust ducts were 2.5m and 2.2m above the roadway floor respectively. 140 pleated filter cartridges and pulse valves injection pipes were used to clean extracted air. | TDCE at Point A was 88.36%.  TDCE at Point B was 88.08%.  RDCE at Point A was 98.22%.  RDCE at Point B was 97.84%. | Reduction in dust concentration Improved environment |
| (Li et al. 2017) | China | Before and After Study | Tunnelling | Tested the application of dry-type filtration dust collection technology to control dust in the construction of a large tunnel. | The dry-type filtration dust collector is made up of the shell, filtration system, cleaning system, discharging system and extraction fan. The exhaust hood extracts dusty air into the filter chamber due to the negative pressure of the extraction fan. The large particles fall due to gravity and the fine particles are trapped the outer wall of the filter cartridge in the filter chamber. The clean air is discharged through the extraction fan. | TDCE of the dry-type filtration dust collection system under lining trolley shotcreting is 88.17%.  RDCE of the dry-type filtration dust collection system under lining trolley shotcreting is 87.28%. | Reduction in dust concentration  Visibility (no measurement) |
| (Hu et al. 2019) | China | Quasi-experimental | Mining - Coal mine roadway | To evaluate the use of an atomization device in the middle of a coal mine excavation roadway. | The atomisation device is mounted on a telescopic bracket on the coal cutter. Pressurised water from the water chamber is sprayed through six fan-shaped nozzles distributed around the walls the chamber. Compressed air from the air chamber flows through the annular chamber and is blown out under high pressure through the seam causing the airflow to impinge on the mist causing secondary.  Compared to water curtain. | TDCE for the atomisation device was 84.1%. TDCE for the water curtain was 59.3%.  RDCE for the atomisation device was 76.4%. RDCE for the water curtain was 33.4. | Reduction in dust concentration |
| (Gottesfeld et al. 2019) | Nigeria | Quasi-experimental | Mining | To reduce respirable silica exposure among artisanal small scale mining communities in Nigeria. | The intervention involved the provision of a bore well, water storage tanks, water misting systems and training sessions on silica dust awareness and exposure reduction practices. Water misting were installed at the inlet and outlets of the crushing and grinding machines. This was created with two commercially available 0.25-inch brass nozzles that operated at 0.4bar and between 0.86 and 1.4L/min. | Mean airborne respirable silica (quartz) was reduced by 80%. | Reduction in respirable quartz exposure |
| (Fang et al. 2019) | China | Quasi-experimental | Mining - Coal mining face and roadway | To determine the optimal ratio of the long-pressure short-pumping system in a fully mechanized coal mining face. | The air curtain generator (Φ1.0m×4m) is fixed into the air supply duct, 15m away from the face. The outlet of the air supply is 10m from the face and the exhaust duct of diameter of 0.6m has a suction head of 1m×1.5m×0.3m, 3m away from the face. The axial air outlet ratio was maintained at 1:3.  Compared with the original dust control. | The overall dust control efficiency was up to 94%. | Reduction in dust concentration  Visibility |
| (Li, P et al. 2019) | China | Quasi-experimental | Mining - Coal mine roadway | To evaluate the effectiveness of a wet mix shotcrete equipment. | The wet-mix shotcrete sand and coarse aggregates were mixed for 20s with cement added after 15s. Water and water-reducing admixtures are then added and mixed for two minutes.  The accelerator was injected with compressed air at the spray gun at a pressure of 0.2 MPa with 1m spraying distance and 90° angle of spraying.  Compared with dry mixing with a rotor-type spray machine. | Average dust concentration decreased by 90% for the wet-mix shotcrete.  Dust control efficiency was increased by 55% with the accelerator. | Reduction in dust concentration |
| (Ge et al. 2019) | China | Quasi-experimental | Mining - Coal preparation plant of a open pit mine | To investigate the use of a remote fogger dust suppression in the receiving pit (ROM pad) of coal mines. | A remote sprayer with a swing function, large coverage, long-range small droplet size and fast speed. It has a wind speed of 30m/s. The angle of the nose of the sprayer is between - 40° and 40° of the central axis.  Compared to the original dust control system. | The average dust control efficiency was up to 93%. | Reduction in dust concentration |
| (Yin et al. 2019) | China | Before and After Study | Mining - Tunnelling Face | To evaluate the use of space-time evolution of dust diffusion with or without an air curtain for dust control. | The forced air duct was 9m from the cutting face with a diameter of 0.6m and a blowing air volume of 298.6m3/min. It was 2.91m above the floor and 0.54m from the left wall of the tunnel. The exhaust dust diameter was 0.6m and an extraction air volume of 349.2m3/min with the duct 4.5m from the cutting face. The air-curtain generator was 20m from the cutting face and had two outlet types. One divided the semi-circular surface of the generator into three equal parts and took 2/3 as the air outlet and the other outlet type divided the curtain into five equal parts and took 3/5 as the air outlet.  Compared to the use of air curtain at different positions from the cutting face. | The air-cleaning efficiency of more than 97.77%. | Air-cleaning effect   Improved visibility |
| (Xu et al. 2019) | China | Before and After Study | Mining - Coal Mining Face | To the advancing support coal mine dust. | The hydraulic support spray dust device had 2.4mm nozzle pore diameter and a spray pressure of 8 MPa. | TDCE was more than 78%. RDCE was more than 83%. | Reduction in dust concentration |
| (Wang, J et al. 2019) | China | Quasi-experimental | Mining - Under the hydraulic support in a coal mining face | To evaluate the efficiency of a multi nozzle atomization interference dust suppression system. | The hydraulic support dust suppression system was formed by a suite of No. 1 nozzles operated at a spray pressure of 8 MPa and had a spray field overlapping coefficient of 0.4  Compared to water misting at different spray field overlapping coefficients. | TDCE of the intervention was 79.38%. RDCE of the intervention was 80.94%. | Reduction in dust concentration |
| (Yang et al. 2019) | China | Quasi-experimental | Mining - Coal Mining Face | To evaluate the effects of hydraulic pressure and the installation angle of nozzles on the performance of external spraying de-dusting system. | The misting system was formed with type-C nozzles operating at a spraying pressure of 8 MPa. Four nozzles were installed around the cutting motor in a spiral uniform pattern while another six were arranged on the rocker arm of two drums with a 30° inclination angle relative to the coal wall.  Compared with the use of the intervention at 2,4 and 6 MPa. | Dust control efficiency at 8 MPa was 90.1%. Dust control efficiency at 2 MPa was 34.25%. Dust control efficiency at 4 MPa was 56.65%. Dust control efficiency at 6 MPa was 77.53%. | Reduction in dust dispersion |
| (Zhou et al. 2019) | China | Quasi-experimental | Mining - Coal Mining Face | To evaluate the effectiveness of a dust removal spray system in a coal mine. | Surfactant-magnetised water was used for the external spray at a spray pressure of 5 MPa. The surfactant-magnetised water was generated by passing the 0.03 wt% surfactant solution through a 350 mT magnetized field, with a water velocity of 4 m/s. Spraying was done with 4 nozzles with an outlet diameter of 1.5 mm. Two of the nozzles were installed on the rocker arm of the coal cutter. | TDCE was 89.7%.  RDCE was 88.6%. | Reduction in dust concentration |
| (Wang, Y et al. 2019) | China | Before and After Study | Mining - Ore pass in an iron ore mine | To evaluate the dust control effect of a high-pressure air curtain combined with gas water spray in the unloading process of metal mine roadway. | The air curtain is formed with a 10 mm diameter tube, extending from the main steel tube with 50 mm, a hole spacing of 30 mm, a spray angle of 30° and a pore outlet speed of 20 m/s.  The gas water spray system is installed in the crosscut with 1.5m between spray heads and uses a nozzle of a radius of 2 mm at a water flow rate to gas flow rate of 0.011. | TDCE reached 74%. RDCE reached 65%. | Reduction in dust concentration |
| (Chen & Liu 2019) | China | Quasi-experimental | Mining - Coal mine roadway | To evaluate the dust control effect of a ventilation system for the simultaneous operation of rock drilling and shotcreting in a tunnel roadway. | A turbulator was placed in the forced air cylinder at the shotcreting area to create a dust control air curtain. An exhaust air outlet (SEAO) was installed in the exhaust duct at the shotcreting area. The turbulator was 20 m from the heading face and the SEAO was 40 m. The forced air volume, pressure-exhaust ratio, the turbulator air volume and SEAO air volume were 300 m3/min, 0.75, 150 m3/min and 180 m3/min, respectively.  Compared with a blanket ventilation system. | The efficiency of the intervention compared with the blank pattern ventilation measured from 35m to 3m away from the heading face is 75.6%, with the highest efficiency of 90% recorded at 35 m from the heading face. | Reduction in dust concentration |
| (Liu et al. 2018) | China  (Liu et al. 2018) | Before and After Study | Mining - Tunnelling Face | To determine the position of a swirling air-curtain generator in a long-forced-short-exhaust ventilation system that gave the best dust control. | The multi-radial swirling air-curtain generator is installed 25m from the heading face in the forced air duct of the FPNA ventilation system. The forced air volume and exhausted air volume were 258.6m3/min and 256.7m3/min respectively. The radial outlet had two sets of five air-out strips, distributed alternately on the radial side of the generator, with a coverage range of 180° and a spacing of 0.05 m.  Compared to different positions of the multi-radial swirling air-curtain generator from the heading face. | Efficiency at the road her header’s driver location is 98.79%. | Reduction in dust concentration  Dust diffusion distance |
| (Liao et al. 2018) | China | Quasi-experimental | Mining - Coal mine roadway | To evaluate the effectiveness of a complex dust suppressant (SSC) used in a coal mine roadway. | The SSC was made from 0.025 wt.% MgCl2.6H2O; 0.01 wt.% NaSO4; 0.015 wt.% PAM; 0.005 wt.% AEO 7; 0.005 wt.% SAS-60; and 0.005 wt.% HPMC. SSC was used in a water curtain dust control system.  Compared with water without SSC. | Average TDCE of SSC was 89.2%.  Average TDCE of water curtain without SSC was 44.7%.  Average RDCE of SSC was 87.7% Average RDCE of water curtain without SSC was 22.1%. | Reduction in dust concentration |
| (Gao et al. 2018) | China | Quasi-experimental | Mining - Coal mine roadway | To evaluate the effectiveness of a swirl nozzle used in a coal mine. | By converting the liquid pressure into kinetic energy, a conical cavity is formed by the liquid eddy in the centre of the swirl chamber. As the water spray spreads in the air, any slight air disturbance breaks the liquid film to form smaller water droplets.  Compared with the traditional nozzle. | The swirl nozzle was up to 23% more effective in controlling dust than the traditional nozzle. | Reduction in dust concentration |
| (Chen et al. 2018) | China | Before and After Study | Mining - Coal mine roadway | To evaluate the dust system in the goaf of a large cross-sectioned coal mine with a fast airflow velocity. | The intervention consists of a roller shutter shaft, dust-collecting net and water curtain. Sprayed water on the wet dust collecting net formed a water film to trap dust.  Compared to water spray system between the hydraulic supports. | The efficiency of the original water spray system was up to 20.3%.  The efficiency of the wet dust collecting nets was up to 69.6%. | Reduction in dust concentration |
| (Sun et al. 2018) | China | Quasi-experimental | Mining - Fully mechanised coal mining face | To evaluate the effects of a local spray closure dedust technique. | The local spray closure dedust technique formed an airflow curtain and spray curtain from the wet scrubber on the shearer. A wet scrubber was used to wet and cover the cutting drums. A spray curtain was formed with Venturi negative pressure dedust device between the shearer and the upright. Water spraying with an atomising system was done between the hydraulic supports. The wet scrubber was arranged with 10 nozzles and flow rate of about ${250m^{3}}/{min}$. The Venturi negative-pressure dedust device was made up of venturi device and cross-cut hybrid nozzles with an X-shaped diversion core.  Compared with the internal and external spray system of the shearer. | RDCE for the local spray closure technique was 26.7% compared with the original dust suppression system. | Reduction in dust concentration |
| (Zhou, G et al. 2018) | China | Before and After Study | Mining - Coal Mining Face | To evaluate the dust control effect of optimised nozzles and their arrangements in narrow and small spaces around the coal cutter. | The cuboid shaped dust removal fan had sets of nozzles set on three sides. The X-type hybrid (No. 6) nozzle with pore diameter of 1.6 mm, was used at a spray pressure of 8 MPa and an atomising angle of 89°. The effective spraying range was 5.7 m, and the flow rate was 6.36 L/min. The dust removal fan was on both sides of the coal cutter and the sprayed water mist on the inhaled air. The water droplets trap the dust which falls and is discharged to the floor. | Average TDCE was 66.5%.  Average RDCE was 61.8%. | Reduction in dust concentration |
| (Han & Liu 2018) | China | Quasi-experimental | Mining - Coal mine roadway | To evaluate effect of dust control measures for ring shape dust source of the cutting head of road header. | The water spray system produced a ring-shaped water mist to cover the dust source using 6 arc fan nozzles.  Compared to the use of full cone nozzles. | TDCE of the spray by arc fan nozzles was 88.1%. TDCE of the spray by full cone nozzles was 65.7%.  RDCE of the arc fan nozzles was 65.4%. RDCE of the spray by full cone nozzles was 86.5%. | Reduction in dust concentration |
| (Wang et al. 2018) | China | Before and After Study | Mining - Coal Mining Face | To evaluate the effectiveness of a two-stage cavitation jet device in a coal excavation face. | The two-stage cavitation jet device consists of primary and secondary single-stage jet pumps with parallel structures and a common import. The jet pumps share the same water supply source while the secondary jet pump's export is connected to the primary jet pump's suction inlet. Surfactant is drawn by secondary pump as pressurised water flows through the primary jet.  The 5mm diameter air atomizing nozzle has an air inlet, circular water inlets each with a diameter of 5mm around the nozzle, and an atomizing chamber where the air further atomises the water and an outlet. | TDCE of the intervention was 80.9%.  RDCE of the intervention was 79.4%. | Reduction in dust concentration |
| (Zhou, Q et al. 2018) | China | Quasi-experimental | Mining - Coal mine roadway | To evaluate the dust control effective of a surfactant magnetised water (SMW) solution. | SMW was produced by passing a solution of 0.03 wt% surfactant and 0.05 wt% NaAc through 350 mT magnetized field at a water velocity of 4 m/s.  Compared to the use of a surfactant solution. | TDCE of SMW was 86.45%. RDCE of SMW was 84.97%.  TDCE of untreated water was 56.23%. RDCE of untreated water was 41.45%.  TDCE of surfactant was 78.32%. RDCE of surfactant was 75.15%. | Reduction in dust concentration |
| (Zhou, Q et al. 2017) | China | Quasi-experimental | Mining that adopts blasting method to dig the roadway | To evaluate the dust control effective of a surfactant magnetised water (SMW) solution. | Fatty acid methyl ester ethoxylate (FMEE) and sodium dodecylbenzene sulfonate (SDBS) were mixed in a mass ratio of 5:1 to produce the surfactant. The SMW was produced by passing a solution of 0.03 wt% of the surfactant through 350 mT magnetized field at a water velocity of 4 m/s. The SMW was sprayed at 3–4 MPa.  Compared with misting with surfactant mended solution (SMS), magnetised water (MW) and untreated water. | TDCE of SMW was 84.75%. RDCE of SMW was 83.54%.  TDCE SMS was 75.46%. RDCE of SMS was 69.73%.  TDCE of MW was 63.65%. RDCE of MW was 56.53%.  TDCE of untreated water was 52.96%. RDCE of untreated water was 38.6%. | Improved atmospheric environment |
| (Lu et al. 2017) | China | Before and After Study | Mining - Coal mine roadway | To evaluate the effectiveness of a new foaming device at the heading face of a coal mine. | A foaming agent adding device is integrated into a foam generator to form foam with an expansion ratio of 38.6 and a spraying distance of 3.4 m. The water pressure was 0.39 MPa  Compared with conventional water spraying. | TDCE was 87.1%. RDCE was 88.3%.  TDCE and RDCE were 2.14 and 2.37 times higher compared with the conventional water spraying. | Reduction in dust concentration |
| (Zhou, G, Xu, M, et al. 2017) | China | Before and After Study | Mining - Coal Mining Face | To improve the efficiency of water misting mechanical nozzles. | The external water spray system consists of No.1 full cone stainless steel nozzles with an orifice diameter of 2 mm and vortex centrifugal type atomization. The spray pressure was 8 MPa and the nozzles were 1.5m away from the shearer’s drum.  Compared with the shearer’s external spray system. | TDCE reached 92%.  RDCE reached 90%. | Reduction in dust concentration |
| (Nie et al. 2017) | China | Quasi-experimental | Mining - Coal Mining Face | To evaluate the effectiveness of a novel external-spraying injection dedusting device for in a fully mechanized coal excavation face. | The intervention was an arch-shaped device made of a rigid water pipe, eight nozzles, cone-shaped caps, an injection tube and suction hoods. It was installed on the left, top and right of the arm cutting arm of the shearer. The nozzles and caps on the bottom are tilted 30° downwards, whereas those on the top are tilted 20° upwards. The cone-shaped cap and suction hood are each equipped with one 20# nozzle with a pore diameter of 2.2 mm.  Compared to the original external-spraying dust device. | TDCE at the driver’s position was up to 71.1%.  RDCE at the driver’s position was up to 70.4%. | Reduction in dust concentration |
| (Zhou, G, Zhang, Q, et al. 2017) | China | Before and After Study | Mining - Coal Mining Face | To evaluate a respirable dust control system at a fully mechanized caving face. | The air-blast nozzle set was arranged on the upper and lower arc surfaces of the shearer rocker arm and the upper-end face of the shearer’s main body perpendicular to the roof. The upper ones sprayed upward, and the lower ones sprayed downward. A water mist curtain is created by a high-pressure micro-fog nozzle installed at the top beam and front canopy of heading supports. | The mean dust-settling rate reaches 91.06%. | Reduction in dust concentration |
| (Roberts & Wypych 2017) | Australia | Before and After Study | Mining -ROM bin in an iron ore mine | To evaluate the effectiveness of a high energy micromist nozzles with lower rates of water consumption for the run-of-mine (ROM) dumping area in an iron ore mine. | The installed EnviroMist (micro-mist) nozzles were operated from 100–300 bar and able to overcome high crosswinds. The EnviroMist nozzle has the same water consumption as the traditional nozzle but operates at flow rates as much as 50% lower than current systems.  Compared to the traditional spray system. | The installed system delivered 100 per cent airborne dust capture. | Reduction in dust concentration |
| (Xia et al. 2016) | China | Before and After Study | Mining - Operatoration and monitoring of crushing plant and belt conveying system | To redesign and rebuilt a coal transfer chute to control the coal dust emission in a coal power plant. | The chute was redesigned with fillets to minimise build-up in the corners. A bigger stilling chamber was installed with several dust curtains to reduce the airflow velocity and assist settle the dust. A dust extraction system that utilises vacuum pressure in the stilling chamber was designed and connected to a misting room with fogging nozzles and a wet cyclone.  Compared to the traditional transfer chute. | TDCE was 96.4% after the modification. | Reduction in dust concentration |
| (Wang, Q et al. 2016) | China | Quasi-experimental | Mining - Coal mining face of a roadway | To test the effectiveness of an internal foam-spraying system for use in a high wind speed heading. | The internal foam-spraying system transported foam through the road header’s inner pipeline to the cutting head. It combines a water-jet suction device with a porous spiral coupling foaming device. The spray nozzle was a of 8 mm diameter and had filtrating screens to prevent blockage. A foam with an expansion ratio of 27 was produced at a water flow of ${1.4m^{3}}/h.$  Compared with traditional foam method and water misting. | TDCE of the new foam method was 89.4%. RDCE of the new foam method was 92.3%.  TDCE of water misting was 34.6%. RDCE of water misting was 31.8%.  TDCE of the traditional foam was 82.9%. RDCE of the traditional foam was 86.4%. | Reduction in dust concentration |
| (Du Plessis et al. 2016) | South Africa | Quasi-experimental | Mining-Haul Road on an open pit iron ore mine | To evaluate the effectiveness of a dust palliative on a haul road of an opencast iron ore mine. | Lignosulphonate-based dust palliative. | RDCE of the lignosulphonate-based dust palliative was 47.3%. | Reduction in airborne particulate concentration |
| (Nie, Ma, et al. 2016) | China | Quasi-experimental | Mining | To evaluate the effectiveness of spraying/negative-pressure secondary dust suppression device for dust control between hydraulic supports. | The intervention was installed at the oil cylinder where the jack of the hydraulic supports is located. It is made of a trumpet-shaped dust extraction port which covered the spraying field of the nozzles. The side dust suction inlets are slanted at an angle of 15° with the direction of the wind flow and inhale dust as the support moves. Three type J nozzles were installed with angles of 0°, 45° and 90° to the horizontal and covered completely the full cross-section between coal walls and hydraulic supports. The spraying pressure was set at 8 MPa.  Compared to the original dust suppression at a spraying pressure was set at 8 MPa. | Average TDCE of the secondary dust control device was 82.0%. Average TDCE of the original dust control system was 55.8%.  Average RDCE of the secondary dust control device was 80.9%. Average RDCE of the original dust control system was 53.6%. | Reduction in dust concentration |
| (Wang, D et al. 2016) | China | Quasi-experimental | Mining - Coal mining face | To test the dust suppression capability of the new foam mixing device in a heading face driven by a powerful road header. | As air flows through the intervention, the foaming agent is added automatically. The foam generated has an expansion foam expansion rate of 28–30 and a spraying distance of 3.0 -3.3m. The device uses air pressure of 0.45 MPa and an airflow rate of 43–45m3/h to draw in the foaming agent.  Compared to water misting. | TDCE for foam was 85.81%. RDCE for foam was 88.66%. TDCE for water misting was 38.72%. RDCE for water misting was 36.09%. | Reduction in dust concentration |
| (Cheng et al. 2016) | China | Quasi-experimental | Mining - Coal Mining Face | To test the effectiveness of selected nozzles and sprays pressures for dust control. | The intervention consists of water misting using the internal, external spray systems of mining equipment or both. Various spray pressure values and nozzle types were tested to obtain optimal results based on the dust characteristics of different positions.  Compared with water misting with different spray pressures and nozzles. | Using optimal nozzles, the dust removal rates of both total dust and respirable dust could exceed 70%. | Reduction in dust concentration |
| (Han et al. 2016) | China | Quasi-experimental | Mining - Coal mine roadway | To evaluate the effectiveness of the arc jet nozzle in a coal mine. | The foam spray nozzle has an extended segment that controls the pattern of the arc jet. The optimal working condition for the single nozzle was a liquid quantity is ${0.375m^{3}}/h$and a gas-liquid ratio of 30.  Compared to other nozzle types. | TDCE for the arc fan nozzle was 85.8%.  TDCE for the flat nozzle was 71.7%.  TDCE for the full cone nozzle was 61.9%.  RDCE for the arc fan nozzle was 82.6%.  RDCE for the flat nozzle was 69.6%.  RDCE for the full cone nozzle was 60.2%. | Reduction in dust concentration |
| (Wang, K et al. 2016) | China | Quasi-experimental | Mining - Coal mine roadway | To examine `the effect of a wetting agent for water injection and spraying on dust control in tunnelling of outburst coal seam. | The complex wetting agent A was made from different surfactants, neutral salts, and chemical additives. It has strong wetting effect even after multiple dilution of the starting concentration. It was used in water injection and then spraying system at the roadway of the tunnelling face.  Compared the performance of untreated water and wetting agent SRJ-1. | Dust control efficiency after using the wetting agent A in water infusion and spray was 93.6% at the tunnelling position.  Compared to using pure water from water infusion, the reduction of dust concentration at the tunnelling position was 44.7% when using wetting agent.  Compared with pure water after using wetting agent A for high-pressure spray, the dust-proofing efficiency of the tunnelling position was 66.7%. | Reduction in dust concentration  Improved work environment |
| (Hu et al. 2016) | China | Before and After Study | Mining - Fully-mechanised coal mining face with high gas content | To study the possibility of implementing water injection through gas drainage boreholes for dust control during the influence of abutment pressure. | A water injection system that utilises the inseam gas drainage boreholes under the influence of abutment pressure. Water injection was performed during daily maintenance at a pressure of 5 MPa and was stopped when a leak is observed on the coal wall. The optimal distance for water injection ahead of the working face (DWAF) was 35m.  Compared to different 10 and 25m of DWAF. | At a DWAF of 35 m, the average reduction rate of coal dust was 25.74%.  At a DWAF of 10 m, the average reduction rate of coal dust was 16.71%.  At a DWAF of 25 m, the average reduction rate of coal dust was 22.93%. | Reduction in dust concentration |
| (Nie, Liu, et al. 2016) | China | Before and After Study | Mining - Coal Mining Face | To evaluate the dust control effectiveness of a multi-direction whirling air curtain on the fully mechanized coal mining face. | A multi-direction whirling air curtain generator was used in FPNA ventilated tunnel, 20m away from the tunnelling face. The air curtain generator was 0.6 m in diameter and 0.95 m in length and had two kinds of air-outlet groups arranged in an alternating manner along the semi-circle. The radial air outlet in one group was divided into 5 equal parts according to the 36° angle with 3 strips. The other was divided into 3 equal parts according to the 60° angle with 2 strips of air outlet. Air and non-air outlet with a ductwork entity of 0.15 m diameter in the middle. Each air-outlet group had two groups: one group was the air-outlets with a width of 0.1 m, and the others had a width of 0.15 m. | The dust control efficiency when the distance from the pressure ventilation outlet to the heading face was 10m is 82.4%.  The dust control efficiency when the distance from the pressure ventilation outlet to the heading face was 20m is 94.4%. | Reduction in dust concentration |
| (Lu, X et al. 2015) | China | Quasi-experimental | Mining - Coal mining face | To test the application of a new complex foaming agent and tested its application in a hard coal road roadway. | As pressurised water flows through the jet device to the venturi foam generator, the foaming agent is automatically added. Compressed air is added in the foam generator to foam which is sprayed through five foam nozzles to cover the cutting head of the road header. The foam nozzle consists of a bevel and two circular arcs which disperse foam between 90-100° without pulverisation.  Compared with water misting. | TDCE for foam reached 85.7% TDCE of water misting reached 37.9%.  RDCE for foam reached 88.1% RDCE for water misting reached 35%. | Air visibility Reduction in dust concentration |
| (Chen et al. 2015) | China | Quasi-experimental | Mining - Fully mechanised coal mine | To evaluate the effectiveness of developed foam from three kinds of foaming agents and four kinds of stable foam agents. | Foam is produced from the foam agent FP-1 with an optimal concentration of 0.4% and a 1% solution of stable foam agent WP-3.  Compared with water misting. | TDCE for foam was 86.3%. RDCE for foam was 72.6%.  TDCE for water misting was 42.5%. RDCE for water misting was 25.2%. | Reduction in dust concentration |
| (Lu, X-x et al. 2015) | China | Before and After Study | Mining - The heading face of a coal excavation roadway | To study a new foam agent adding device for foam dust control. | As pressurised water flows through the jet device, which is made of hyaloid organic glass, the foaming agent is automatically added. Using a water flow rate of ${1.5m^{3}}/h,$ the foaming generator produces foam of expansion ratio of 20 – 22 and has a spraying distance of 3.0−3.2m.  Compared to the previous foaming technology and water misting. | The foam dust suppression efficiency reaches up to 86.5%.  The air visibility of the excavation roadway increases from less than 1.0m to 5.3m.  Foam efficiency increased by 12.6%−16.5% compared with the previous foam technology.  The foam efficiency is 2.53 times higher than that of water misting. | Reduction in dust concentration  Increased visibility |
| (Wang et al. 2015) | China | Quasi-experimental | Mining - Coal mine roadway | To evaluate the dust control effectiveness of an optimized foam generator, distribution support and nozzle. | The foaming system has a self-suction part which automatically draws in air and foaming agent as pressurised water flows. The foam-producing part consist of the swirler that accelerates the mixing of air and liquid to produce foam at ${60m^{3}}/h$. The foam is sprayed through four arc-fan nozzles at a water flow of ${1m^{3}}/{h.}$ The intervention consists of two foam generators.  Compared with the traditional foam generating system. | TDCE for the new foam was 87.3%. RDCE for the new foam was 85.9%.  TDCE for traditional foam was 86.4%. RDCE for traditional foam was 83.7%. | Reduction in dust concentration |
| (Wang et al. 2014) | China | Quasi-experimental | Mining - Coal mining face | To evaluate the dust control effectiveness of an air self-suction foam generator. | The air self-suction type foam generator utilises the eductor pump principles to operate. As pressurised water flows through the conical nozzle, air and foaming agents are automatically added. The water-air-foaming agent mixture moves to the foaming chamber where foam is produced. The foam nozzle has a fast connector, a semicircular injection cavity, tapered vanes, and an arc injection groove, where the width of the arc injection groove. The arc shape of the sprayed foam is due to the arc injection groove.  Compared to water misting. | TDCE of foam reached 88.2% at the driver’s position.  TDCE of water misting reached 29.8% at the driver’s position.  RDCE of foam reached 85.8% at the driver’s position.  RDCE of water misting reached 21.4% at the driver’s position. | Reduction in dust concentration |
| (Ren, W et al. 2014) | China | Quasi-experimental | Mining - Mechanised coal mine | To evaluate the effectiveness of a foam technology and equipment to overcome the dust control challenges associated with water spray and dust collecting fan at a mining face. | The foaming agent comes from a mixture of different surfactants and an auxiliary agent. The foam is formed with 0.5% of the foaming agent. The foam generating system utilises ${1m^{3}}/h$ of pressurised water, ${40m^{3}}/h$ of compressed air and 5 kg/h of the foaming agent to produce foam with a foaming multiple of 40.  Compared with water misting. | TDCE of the new foam is 2.11 times that of water spraying.  RDCE of the new foam is 1.72 times that of water spraying. | Reduction in dust concentration |
| (Han et al. 2014) | China | Before and After Study | Mining - Coal Mining Face | To study the effect of forced ventilation on water spraying on the cutting head of road header. | The ventilation duct with Coanda effect helped to change the wind distribution. It partly changes the original axial wind in the forced ventilation duct supplied to heading face to rotating wind or radial wind.  Compared to water misting. | TDCE of water misting and VDCE was 69.4%. TDCE of water misting was 58.9%.  RDCE of water misting and VDCE was 66.9%. RDCE of water misting was 57.6%. | Reduction in dust concentration |
| (Ren, T et al. 2014) | Australia | Before and After Study | Mining - Coal mine roadway | To study the airflow and dust flow behaviour above the bin in a coal mine to develop a dust control measure. | The new water misting system had four sprays nozzles on each side of the sizer. Each nozzle was a flat fan nozzle with standard capacity. | RDCE of the water misting was 68% along the belt roadway. | Reduction in dust concentration |
| (Shi et al. 2013) | China | Before and After Study | Mining - Tunnelling Face | To test the dust control effect of a tunnel wet dust precipitator which is free of filter blockages and can be used in mines with limited space. | The tunnel wet dust precipitator has a centrifugal net disc which rotates when the motor in the centre of the disc operates. Inhaled dusty air is sprayed by the nozzles in front of the disc to wet dust. The centrifugal force aids in the separation of the dust into the collecting tank. The clean air flows into the tunnel. | The average collection efficiency of the wet dust precipitator was 80.31%. | Reduction in dust concentration |
| (Zongyin 2013) | China | Before and After Study | Mining / Crushing Station of copper mine | To evaluate a high-pressure spray dedusting mechanism using high pressure spray pump in an underground mine. | The high-pressure spray dust-settling system was made up of several nozzles around the perimeter of the crusher. Water spray pressure of 8 MPa ~ 10 MPa was used and above the feeder machine a low-pressure spray of 0.5 MPa was adopted, providing a flow rate of 128 ~ 160 L/min. Some mist particles become charged during the high-pressure spray process. | Dust concentration reduced by approximately 90%. | Reduction in dust concentration |
| (Wang et al. 2013) | China | Before and After Study | Mining - Coal Mining Face | To validate the dust control effect of a foam dust suppression technology. | Foaming agent was automatically added in the integrated foam generating device as pressurised water flowed. Compressed air is added to the foaming agent and water mixture and by jet flow action, entrainment and turbulence foam is generated. Spraying was done with six fan-shaped foam spray nozzles installed on the road header. Nozzle angle was between 18-120° spraying about ${7-9m^{3}}/h$ of foam per nozzle.  Compared to water misting system. | TDCE of the foam technology was 86.2%. TDCE of water misting was 23.9%.  RDCE of the foam technology was 84.4%.  RDCE of water misting was 18.5%. | Reduction in dust concentration |
| (Yin et al. 2013) | China | Before and After Study | Mining - Molybdenum ore open pit mine | To evaluate the dust control effect of a drill bit designed to control down-the-hole (DTH) drilling dust. | The DTH reverse circulation air hammer drilling system uses compressed air for drilling. The air carries the cuttings upward through the central passage of the drill tool and discharges the cuttings. | No visible drilling dust escaped from the borehole entrance when used for downward drilling. | Reduction in visible dust concentration |
| (Ren et al. 2013) | Australia | Before and After Study | Mining - Coal Mining Face | To evaluate the effect of a water mist-based venturi units used for longwall dust control. | The venturi system consists of a tapered section and ultrasonic atomizers. The high velocity air stream breaks the water ejected from the nozzle which then collides with a resonator placed in front of the nozzle. The water mist is further shattered into fine droplets. | RDECE was up to 30% when the units are installed on a longwall. | Reduction in dust concentration |
| (Zhou et al. 2013) | China | Before and After Study | Mining - Coal Mining Face | To determine the best air-draft volume and arrangement mode of forced-exhausted fan drum and dedusting fan to control dust. | The forced air duct and exhaust duct were 30m and 1m away from the tunnelling face respectively. Each dust was about 0.4m away from the nearest tunnel wall and 3m away from the floor. The air-draft volume was ${600m^{3}}/{min}$ and a KCS-700 wet dedusting fan was used. | TDCE was 96.1% when using the closed-end dust control system.  RDCE was 96.1% when using the closed-end dust control system. | Reduction in dust concentration |
| (Colinet et al. 2013) | USA | Quasi-experimental | Mining - Coal mining areas | To evaluate dust control effect of a flooded-bed scrubber in a coal mine. | The fan-powered flooded-bed scrubber was used in a 20-ft cuts with exhaust face ventilation and an extended curtain setback. Inhaled dusty is cleaned by passing it through a wetted filter panel. The cleaned air is discharged into the mine. Cleaning of the 30-layer scrubber screen was done after each cut with water and the scrubber inlets and ductworks were cleaned before the start of shift. | Operation of the scrubber reduced dust concentrations up to 91% in the mine. | Reduction in dust concentration  Reduction in quartz concentration. |
| (Cheng, Nie, Zhou, Yang, et al. 2012) | China | Before and After Study | Mining - Coal mine roadway | To evaluate the dust control effect of an air curtain formed by a mural cylinder developed from new light polymer materials. | The forced air duct and exhaust duct were 5m and 1m away from the tunnelling face respectively. The force air duct had 1m diameter and the diameter of the exhaust duct was 0.8m. Both ducts were away from the nearest tunnel wall with the duct axis 3.5m away from the ground. The mural cylinder was 5m long and was located 27m away from the tunnelling face. | TDCE was 97.4%.  RDCE was 96.8%. | Reduction in dust concentration |
| (Zhou, Wang, et al. 2012) | China | Quasi-experimental | Mining - Coal Mining Face | To improve the dust removal system in full rock heading face. | The intervention used a fan vibration twist-action type dust removal system to modify the existing dust control made up of dust isolation, dust removal fan and dust extraction. A drawer -type suck about was placed on the turntable rear cover and a column type cylinder with a flat suction hood was fixed on the digging machine right into the body front. | TDCE of the improved dust control was 95.1%. RDCE of the improved dust control was 96.1%.  TDCE for the original dust control was 66.3%. RDCE for the original dust control is 66.4%. | Reduction in dust concentration |
| (Xie et al. 2012) | China | Before and After Study | Mining - Coal mine roadway | To evaluate the duct control effect of an enclosed dust control system (EDCS) by studying the relationship between airflow and dust distribution at the face of ab coal roadway. | The EDCS was made up of a wet vibration dust removal fan, wire muffler, high-strength vacuum duct, rotating suction device, and rib attaching duct.  The inhaled dusty air flows through the vibration filtering plate. The water sprayers sprayed on the plate and enhanced the capture of dust. Dust particles are separated from water in the dust-water separator and the clean water is discharged or reuse with the air discharged. | The dust control efficiency in the working area of the roadway ranged was up to 95%. | Reduction in dust concentration |
| (Wang et al. 2012) | China | Before and After Study | Mining - Coal Mining Face | To evaluate the dust control effect of a new foaming agent adding device. | The new foaming agent automatic adding device is made of a main pipe, filter, jet, branch pipe, shutoff valve, check valve, needle valve, hose connectors and suction hose. The flow of high-pressure water automatically draws in foaming agent to mix with the pressure water due to the negative pressure formed by the jet. Precise foaming agent addition is enhanced by the control of the needle valve.  Compared with a conventional foam technology. | The new foam adding device achieved 84.4% dust suppression efficiency.  The old foaming device achieved 84.6% dust suppression efficiency. | Reduction in dust concentration |
| (Joy 2012) | USA | Cross Sectional Study | Mining | To evaluate the effectiveness of MSHA regulatory standard in reducing coal dust. | The regulation provides the opportunity for a reduction of the respirable dust standard when the quartz content of airborne respirable dust exceeds 5% by weight.  Effects of field controls compared with the regulatory requirement. | The legal required for respirable quartz exposure control did not reliably reduce miner exposure below ${100g}/{m^{3}}$quartz. | Reduction in respirable quartz exposure |
| (Ren et al. 2012) | China | Quasi-experimental | Mining - Fully mechanised coal mining face | To evaluate the dust control effect of a new foam technology. | Three surfactants were mixed with sodium silicate and sodium chloride (auxiliary agents) to generate the new foam foaming agent. The concentration of the foaming agent was 0.5%. The foam generator is based on the turbulent jet theory providing a strong mixing intensity.  Compared with water misting. | TDCE for the foam was 84.3%. RDCE for the foam was 68.85%.  TDCE for water misting is 39.45%. RDCE for water misting is 39.95%. | Reduction in dust concentration |
| (Cheng, Nie, Zhou, Yu, et al. 2012) | China | Before and After Study | Mining - fully mechanized caving coal face | To evaluate the dust control effect of a water injection technique in low permeability of coal seam. | A penetration agent was developed from ten kinds of ion and anionic surfactants for the infusion. When the mass concentration of the penetration agent reaches 0.05%, the surface tension is 31.18 mN/m. The water infusion pressure fluctuates between 7 MPa and 25 MPa during application. | Average TDCE was 32.9%.  Average RDCE was 33.8%. | Reduction in dust concentration |
| (Zhou, Nie, et al. 2012) | China | Before and After Study | Mining - Coal Mining Face | To evaluate the dust control effectiveness of wet dedusting system. | The spiral wind generator which is placed at the top of the winning machine in front of the driver forms spiral air curtain along the cross-section of the roadway. The forced air duct is 8m to 14m away from the heading face and the exhaust air duct is 3m away from the heading face. | TDCE of the whole workplace was 93.3%.  RDCE of the whole workplace was 92.5%. | Reduction in dust concentration |
| (Du et al. 2011) | China | Quasi-experimental | Mining-Haul Road on an open pit iron ore mine | To evaluate the road dust suppression performance of a dust suppressant. | The dust suppressant was formed by 20% magnesium chloride, 0.1% water-soluble polymer, 0.08% SDBS and 1.5% addictive agent. The average spray volume of dust suppressant was ${2.37L}/{m^{2}}$.  Compared with untreated water misting. | The moment dust raising concentration behind a driving truck on the tenth day was ${4.9mg}/{m^{3}}$. | Reduction in dust concentration |
| (Wang et al. 2011) | Singapore | Before and After Study | Mining - Coal Mining Face | To evaluate the performance of a two-phase foam preparation system. | Water is pumped into pipelines with the simultaneous addition of a proportional foaming agent using a ratio mixer. The foaming agent-water mixture flows into the foam generator which also inhales air. Under the effect of violent mixing, jet, roll suck and turbulent functions the foam generator produces stable foam.  Compared with water misting. | TDCE of the foam reached 90.5%.  RDCE of the of the foam reached 85.4%. | Reduction in dust concentration  visibility of the heading face |
| (Potts & Reed 2011) | USA | Quasi-experimental | Mining - Limestone and Coal open pit mines | To evaluate the effectiveness of an air blocking shelf for dust control on blasthole drills. | The air-blocking shelf drill shroud was made of light gauge angle iron and thin conveyor belting material with the angle iron bolted to the inside perimeter of the shroud. A complete coverage of the parametric cross-section was ensured with the design of the shroud and helped to disrupt the airflow from striking the ground and redirect it towards the centre of the enclosure to be extracted by the LEV. The redirection of air reduced dust leakage from underneath the shroud. | Dust reductions in the immediate vicinity of the shroud were reduced up to 81% and up 70% in areas surrounding an operating blasthole drill. | Reduction in dust concentration |
| (Gurley et al. 2010) | USA | Before and After Study | Mining - Coal Mining Areas | To verify the dust control effect of a second line of defence added to a spray system in room-and-pillar mining areas. | The continuous miner chassis spray system was modified to include additional sprays in the loading pan, on the left side of the main chassis, and as a "second line of defense5' (SLD) on the main chassis. Total water volume for sprays on the modified CM was 155 1pm. SLD sprays were located 4.9 m (196 in.) from the leading edge of the cutting drum on the top of the CM chassis. SLD sprays were oriented towards the cutting drum at an upward inclination of 45 degrees. | Results showed up to 50% reduction of dust.  Quartz reduction rate was up to 25%. | Reduction in respirable quartz exposure |
| (Alexander et al. 2016) | USA | Quasi-experimental | Oil and Gas -transfer of sand into sand movers for hydraulic fracturing. | To evaluate the dust control effect of NIOSH mini baghouse retrofit assembly (NMBRA) used to control dust during filling of sand movers. | The mini baghouse retrofit assembly was fixed the thief hatches on top of sand movers during filling. It was made up of a baseplate and clamping assembly and two sections of ductwork connected to a 48-cm diameter, 122cm long section of baghouse filter material. | Average RDCE was up to 98%.  Average RCS dust control efficiency was up to 99%. | Reduction in dust concentration |
| (Alexander et al. 2018) | USA | Before and After Study | Oil and Gas | Evaluated the effectiveness of the 3rd generation (NMBRA) used the transfer of propellant for hydraulic fracturing. | The NMBRA has no moving parts and requires no power source. The dusty air is passed through a suspended filter bag inflated by air pressure produced by the pneumatic transfer of sand from delivery trucks to sand movers. The filter bag acts as a substrate for the formation of a dust cake. | Reductions of airborne respirable dust were estimated above 99%.  Reductions in airborne RCS ranged from 98 to 99%. | Reduction in dust concentration |
| (Shang 2014) | China | Before and After Study | Port and shipping industries | To evaluate the dust control effect of the Dry-Cleaning Mode as a dust control model in port dust control. | The “Dry Cleaning Technology” consists of several adopted dust control measures used to reduce dust based on the actual condition of the port. | Dust control effect increases by 40% after adopting the Dry-Cleaning Mode. | Reduction in dust concentration |
| (Hedges et al. 2010) | Australia | Before and After Study | Quarries | To evaluate the impact of an air-cleaning device on the air quality inside an excavator cabin in a quarry. | RESPA is a manufactured product of Sy-Klone international and combines the technology of motorised PFP (precleaner, filter and pressuriser) unit. RESPA puts the cabin under positive pressure. The RESPA PFP filter (HEPA 0.3 micron at 99.997% efficiency). | Dust control efficiency when RESPA is used in the excavator cabin reached 57.1%. | Reduction in dust concentration |
| (Qi & LO 2016) | USA | Quasi-experimental | Stone Processing - Stone countertop fabrication | To evaluate the effectiveness of dust control measures adopted in stone countertop fabrication and installations. | The polishers and some grinders were equipped with a centre water feed feature, but the grinders used with turbo blades to cut thin stone strips released from a water hose that discharged at the edge of the turbo blade instead of a water feed feature. Two LEVs were provided primarily for the grinders working with different blades. The LEV was equipped with high-efficiency particulate air filters.  Compared with OSHA PEL and NIOSH REL. | The short-term RCS exposure was ${122.9g}/{m^{3}}$ for the polishers, and ${583.2g}/{m^{3}}$ for the grinders.  Wet grinding and wet polishing of engineered stone may still lead to over exposure to RCS. | Reduction in respirable quartz exposure |
| (Zhang et al. 2014) | China | Quasi-experimental | Stone Processing workshop | To investigate the dust control methods in medium size stone processing enterprises. | The dust removal method was made up of dust collecting hood, windpipe, blower, dust removal equipment, and dust transport device. The extracted dust is cleaned in the bag-type dust collector. The clean air is released into the air. | The dedusting efficiency reached 98.75%. | Reduction in dust concentration |
| (Liu, Nie, Hua, Peng, et al. 2019) | China | Quasi-experimental | Tunnelling - Siltstone tunnel | To determine the optimal distance between the tunnel face and the pressure ventilation ducts for effective dust control. | The tunnel used a long-duct forced and short-duct exhaust ventilation system with the forced air duct outlet 35 m from tunnel face and the extraction duct 3m away from the tunnel face. The forced air-flow rate was ${380m^{3}}/{min}$ and the exhaust airflow rate was ${305m^{3}}/{min}$.  Compared to the Chinese Coal Mine Safety Regulations on dust control. | Dust concentration was reduced below ${18.2mg}/{m^{3}}$ at the cross-section 10 m from the tunnel face. | Reduction in dust concentration   Dust diffusion distance |
| (Liu, Nie, Hua, Jia, et al. 2019) | China | Before and After Study | Tunnelling | To evaluate the effect of different extraction flow rates in a TBM ventilation system on dust control. | The tunnel uses a FPNA ventilation system with a pressurized air duct diameter of 1000 mm and a secondary pressurized air duct diameter was 600 mm as was the extraction duct. The primary pressurized air flow rate was ${20m^{3}}/s$ and the air flow rates of the secondary pressurized and the dedusting fan were ${8m^{3}}/s$. | The dust control efficiency when the dust extraction flow rate was up to 72.05%. | Dust diffusion distance   Reduction in dust concentration |
| (Warden & Warden 2019) | Canada | Before and After Study | Tunnelling | To evaluate a dust control solution that using a wet de-duster high dust road-header tunnelling operation. | Dusty air is extracted by the De-Duster where the integrated impeller mixes the dusty air and water. The dust-laden water travels around the motor in a sealed compartment so to keep the motor operating in clean air. After leaving this bifurcated section it is again sprayed with water using an internal water spray system. The dust-laden water is separated from the air stream using a series of mist eliminators and impingement panels. | Average dust reduction efficiency was 95.3% at the road header.  Dust reduction efficiency at Scoop #1 and #2 reached 99%. | Reduction in dust concentration |
| (Cheng et al. 2013) | China | Quasi-experimental | Tunnelling | To assess the effect of an eddy air-curtain dust-controlled system in hard rock mechanized road tunnel. | A mural cylinder of 5m was used in FPNA ventilated tunnel, 27m away from the tunnelling face. The forced air duct and the exhaust dust each has a diameter of 0.8m with an axis of 3.5m to the nearest wall and 0.4m to the ground. The forced air duct is 5m from the tunnelling face and the exhaust duct is 1.25. The forced air volume in the working face is ${400m^{3}}/{min}$ and the exhaust air volume is about 550m3/min ${550m^{3}}/{min}$. | Average TDCE was 97.4%.  Average RDCE was 96.8%. | Reduction in dust concentration |
| (Jian et al. 2012) | China | Before and After Study | Tunnelling | To test the dust control effectiveness of a water jet vacuum device. | A rotating cone jet vacuum formed by the negative pressure zone is generated to control dust as high-pressure water jet from the spray nozzle while been rotated at high speed. Nozzles were 3.5m from the tunnel face. | Dust control efficiency was up to 21.43%. | Reduction in dust concentration |

**Supplementary Sheet 2b**

***Table 1. PPE improvement studies***

| **Title** | **Country of Study** | **Design** | **Industry study implemented** | **Aim of the study** | **Intervention** | **Outcome Measures** | **Study results** |
| --- | --- | --- | --- | --- | --- | --- | --- |
| (Robertsen et al. 2020) | Norway | Randomised controlled (RCT)/ before-and-after study. | Smelter industry | To assess the effect of a knowledge-based intervention on dust control knowledge, attitudes and use of respiratory protective equipment of smelters | Group 1: fit testing with information about respirators. Group 2: dust exposure and health effects seminar and fit testing. The Control group did not receive any intervention | Intention to use RPE  Rate of respirator use | No observed increase in the intention or frequency of respirator use.  The perception of RPE being an inconvenience decreased.  The intervention improved the attitudes toward the use of respirators and projected a more positive view of the organization.  There was no significant change in knowledge about respirators in the control group. |
| (Chen et al. 2019) | China | Cluster randomized controlled trial/before-and-after study. | Small and medium-sized enterprises (SMEs) that use organic solvents | To evaluate the effect of a behavioural intervention on the appropriate use of respirators in SMEs | The top-down intervention (TDI) group received safety training and mHealth (mobile messages sent to participants).  The comprehensive intervention group received the TDI and a peer education where workers were trained to train other workers. Peer educators received online support.  The control group did not receive any intervention. | Appropriate use of RPE  Level of occupational health knowledge, attitude toward RPE use.  Participation in occupational health check-ups. | The interventions did have any significant effect on the appropriate use of RPE at three months.  Appropriate RPE use increased by 12% in the TDI group and 20.2% in the comprehensive group at the end of the study period. The control group remain almost the same over the period.  Age, sex and education did not significantly affect the appropriate use of RPE. |
| (Shamsi et al. 2016) | Iran | Quasi-experimental/before-and-after study. | Subway construction activities | To assess the effects of a social marketing intervention in promoting the use of PPE in subway construction activities. | The intervention group received helmet, a dust mask and safety gloves. To promote use of PPE, stickers with messages were fixed on helmets, pamphlet highlighting the benefits of PPE use were distributed, and unschooled participants received face-to-face counselling.  The control group did not receive the intervention | Increased use of PPE | The intervention significantly increased the use of helmets and respirators.  There was no significant difference in the use of safety shoes, hand gloves and safety clothes.  Education level, work history and daily working hours did not significantly influence the use of helmets.  By showcasing the benefits and/or reducing the tangible and intangible costs of PPE use behaviour, the uptake of that behaviour can be improved.  Self-reported scores were higher than observed behaviour scores. |
| (Snipes et al. 2015) | USA | Before and after study | Farming | To assess whether the provision of PPE increases use among farmworkers | The study participants received pretested hand gloves, safety glasses, long-sleeved shirts and daily mHealth messaging to cue PPE use. | Use of PPE use | The intervention significantly increased the use of safety glasses and hand gloves.  The use of long-sleeved shirts, pants, and hats showed no significant increase after the study.  Gender, language or income did not influence significantly influence the use of PPE. |
| (Woith et al. 2015) | Russia | Quasi-experimental/before-and-after study. | Health Care/Regional TB hospital | To assess whether the use of a photovoice intervention can change the attitudes and behaviours of healthcare workers aimed at reducing nosocomial tuberculosis. | Participants in the Photovoice intervention photographed someone or something important to them and wrote a short note about how the person or something motivates them to use and fit-check respirators. Posters were produced from photographs and narratives and displayed at the workplace.  There was no control group. | Intention to wear and to fit-check respirators | The intervention significantly increased the intention to wear and fit check respirators.  Belief, subjective norm, and perceived behavioural control were significantly correlated with the intention to use and fit check respirators. |
| (Tovar-Aguilar et al. 2014) | USA | Times series quasi-experimental design with non-randomized control group | Farming/Citrus harvesting | To assess the effectiveness of a community-based prevention marketing model in promoting the adoption of safety glasses among citrus farmers. | Some citrus harvesters were trained as community health workers (CHWs), wore glasses most of the day, and provided educational messages and first aid to co-workers and kept activity diaries  The control group received safety glasses but there were no CHWs | Changes in worker perception about the use of safety glasses  Increased use of PPE  Reduction in eye injury | CHWs significantly improved the use of safety glasses. The more time the CHW spent with the crew, the greater the use of safety glasses.    The intervention changed the perception that wearing safety glasses reduced productivity and earning capacity.  The number of eye irritation recorded reduced after the study.  Receiving help from a CHW significantly increased the use of safety glasses more than just knowing the CHW. These had better influence in using safety glasses than not knowing the CHW. |
| (Pounds et al. 2014) | USA | Cross Sectional Study | Farming/activities that exposure farmers to respiratory illness | To assess the effects of a social marketing intervention in promoting the use of respiratory protective devices (RPD) use among farmers. | Based on consultations with the farmers, the intervention was designed to YouTube videos to address beliefs, knowledge and attitude related to RPD use. Emails, and farm shows were used to raise awareness and direct farmers to the videos. Bags with motivational messages were used to ensure farmers had PPE close to them.  There was no control group. | Knowledge of PPE usage  Intention to use RPDs in dusty environments | The response rate of participants was low, but the study had a positive impact on the knowledge about the health risks of dust exposure and increased the likelihood of using an RPD.  The educational emails changed the frequency of use of dust mask in 40% of the participants. |
| (Hennessy & Dynan 2014) | USA | Quasi-experimental | Health Care/Cancer treament center | To improve compliance with PPE use by nurses administering chemotherapy in the outpatient setting. | The study utilised a multifaceted intervention with no control group.  Mandatory comprehensive education about the hazards and standards of exposure to chemotherapy was implemented and incorporated into the skills training programme. Best PPE practices were identified and implemented, and PPE compliance report was issued quarterly. A safe-handling awareness campaign was launched. A monthly audit and feedback were initiated, and the results were shared and tracked. | PPE use compliance | The intervention improved PPE compliance.  Education and awareness alone do not result in a substantive change in PPE compliance practice. |
| (Adewoye et al. 2014) | Nigeria | RCT/ before-and-after study. | Small-scale electrical arc welding enterprises | To evaluate the impact of a training intervention on the safety awareness and practices of small-scale welders. | The health education intervention was completed in five sessions and was delivered through health talk, posters, flip charts, and audio-visual materials.  Control group did not receive the training intervention | Increased PPE awareness Increased use of PPEs | The intervention significantly increased awareness about hand gloves,  eye googles, welding helmet, face mask, apron and protective clothing.  The intervention significantly increased the use of facemask, hand gloves and safety boots. |
| (Seixas et al. 2011) | USA | RCT/ before-and-after study. | Construction Industry | To assess the effect of a multi-facet intervention to promote the adoption of hearing protection devices among construction workers. | Group 1: baseline (BL) training,  Group 2: BL training and Toolbox (TB) training,  Group 3: BL training and the noise level indicator (NLI), and  Group 4: Received all interventions (BL, TB, and the NLI).  Control group only received the baseline training. | increase in HPD | Whiles the intervention increased the use HPDs, there was a reduction in use at during follow-up but remained high compared to per-intervention.  Participants in the BL and TB group had the highest HPD use. Participants who received BL, TB, NLI had the lowest HPD use rate, but it was more sustained of the study period.  The use of HPD was lower in the BL and TB group at post-intervention compared to preintervention. |
| (Donham et al. 2011) | USA | Cohort study (Prospective) (Randomised Control) | Farming | To evaluate the respiratory hazards reduction effect of the Certified Safe Farm (CSF) intervention. | The study utilised a multifaceted intervention consisting of risk assessment of all operations, clinical screenings, on-farm safety audits, farmer health and safety education, and incentives for achieving or retaining CSF status. Participants benefited from quarterly newsletter, web site access, and yearly group educational meetings.  Control group did not receive any intervention. | Increased use of PPE and   Decreased risk of ODTS | The CSF intervention increased the use of PPE (including respiratory protection) and remained relatively stable over the course of the study.  The use of respirators was more prominent among participants under 60 years.  The use of HPD was more prominent among younger workers, highly educated employees, those with self-reported hearing problems or employees in ‘other’ trades.  The prevalence of acute respiratory condition ODTS significantly reduced in the intervention group suggestion use of PPE may be a contributing factor. |
|  | USA | Quasi-experimental/before-and-after study. | Construction - Renovation | To evaluate how effectively a theory-based intervention can influence the adoption of ventilated drywall sanding tools by drywall finishing workers | The intervention utilised training sessions to create awareness about drywall dust hazards and had hands-on practice with a ventilated sander. Hard hat stickers and printed T-shirts were used to cue the use of PPEs  The control group did not receive any intervention. | Adoption readiness for ventilated sanders.  Trust in technology | Although the intervention improves adoption readiness significantly, it is short lived as there was no difference between the study group and control group at follow-up.  The intervention does not increase health knowledge, perceived risk to health, or trust in organization. |

**Supplementary Sheet 2c**

***Table 1. Dust control studies***

| **Title** | **Country of Study** | **Design** | **Industry study implemented** | **Aim of the study** | **Intervention** | **Outcome Measures** | **Study results** |
| --- | --- | --- | --- | --- | --- | --- | --- |
| (Weidman et al. 2016) | USA | Quasi-experimental/before-and-after study. | Construction - Renovation | To evaluate how effectively a theory-based intervention can influence the adoption of ventilated drywall sanding tools by drywall finishing workers | The intervention utilised training sessions to create awareness about drywall dust hazards and had hands-on practice with a ventilated sander. Hard hat stickers and printed T-shirts were used to cue the use of PPEs  The control group did not receive any intervention. | Adoption readiness for ventilated sanders.  Trust in technology | Although the intervention improves adoption readiness significantly, it is short lived as there was no difference between the study group and control group at follow-up.  The intervention does not increase health knowledge, perceived risk to health, or trust in organization. |

Adewoye, KR, Awoyemi, AO, Babatunde, OA, Atoyebi, OA, Salami, SK & Issa, FY 2014, ‘Effect of health education intervention on the awareness and use of personal protective equipments among small scale electric arc welders in Ilorin, Nigeria’, *Indian Journal of Occupational & Environmental Medicine*, vol. 18, no. 1, pp. 3-8.

Akbar-Khanzadeh, F, Milz, SA, Wagner, CD, Bisesi, MS, Ames, AL, Khuder, S, Susi, P & Akbar-Khanzadeh, M 2010, ‘Effectiveness of dust control methods for crystalline silica and respirable suspended particulate matter exposure during manual concrete surface grinding’, *J Occup Environ Hyg*, vol. 7, no. 12, pp. 700-11.

Alexander, BM, Esswein, EJ, Gressel, MG, Kratzer, JL, Feng, HA, King, B, Miller, AL & Cauda, E 2016, ‘The development and testing of a prototype mini-baghouse to control the release of respirable crystalline silica from sand movers’, *J Occup Environ Hyg*, vol. 13, no. 8, pp. 628-38.

Alexander, BM, Esswein, EJ, Gressel, MG, Kratzer, JL, Feng, HA, Miller, AL, Cauda, E & Heil, G 2018, ‘Evaluation of an improved prototype mini-baghouse to control the release of respirable crystalline silica from sand movers’, *J Occup Environ Hyg*, vol. 15, no. 1, pp. 24-37.

Bao, Q, Nie, W, Liu, C, Zhang, H, Wang, H, Jin, H, Yan, J & Liu, Q 2020, ‘The preparation of a novel hydrogel based on crosslinked polymers for suppressing coal dusts’, *Journal of Cleaner Production*, vol. 249, p. 119343.

Cai, P, Nie, W, Liu, Z, Xiu, Z, Peng, H, Du, T & Yang, B 2020, ‘Study on the air curtain dust control technology with a dust purifying fan for fully mechanized mining face’, *Powder Technology*, vol. 374, pp. 507-21.

Chen, D, Nie, W, Cai, P & Liu, Z 2018, ‘The diffusion of dust in a fully-mechanized mining face with a mining height of 7 m and the application of wet dust-collecting nets’, *Journal of Cleaner Production*, vol. 205, pp. 463-76.

Chen, L & Liu, G 2019, ‘Airflow-Dust Migration Law and Control Technology Under the Simultaneous Operations of Shotcreting and Drilling in Roadways’, *Arabian Journal for Science and Engineering*, vol. 44, no. 5, pp. 4961-9.

Chen, W, Li, T, Zou, G, Renzaho, A, Li, X, Shi, L & Ling, L 2019, ‘Results of a cluster randomized controlled trial to promote the use of respiratory protective equipment among migrant workers exposed to organic solvents in small and medium-sized enterprises’, *International Journal of Environmental Research and Public Health*, vol. 16, no. 17, p. 3187.

Chen, X, Hu, H, Xu, Y, Zhang, Y & Yang, G 2015, ‘Experimental investigation of foam dedusting agent in underground coal mine’, *Materials Research Innovations*, vol. 19, no. sup8, pp. S8-508-S8-11.

Cheng, J, Zheng, X, Lei, Y, Luo, W, Wang, Y, Borowski, M, Li, X, Song, W, Wang, Z & Wang, K 2020, ‘A Compound Binder of Coal Dust Wetting and Suppression for Coal Pile’, *Process Safety and Environmental Protection*.

Cheng, W, Ma, Y, Yang, J & Sun, B 2016, ‘Effects of atomization parameters of dust removal nozzles on the de-dusting results for different dust sources’, *International Journal of Mining Science and Technology*, vol. 26, no. 6, pp. 1025-32.

Cheng, W-m, Nie, W, Zhou, G & Yang, J-l 2013, ‘Research on Eddy Air-Curtain Dust Controlled Flow Field in Hard Rock Mechanized Driving Face’, *Journal of Networks*, vol. 8, no. 2, pp. 453-60.

Cheng, WM, Nie, W, Zhou, G, Yang, JL, Du, WZ & Zhang, XH 2012, *Numerical simulation of eddy air-curtain dust controlled flow field in hard rock mechanized driving face*, Kunming, 16609336 (ISSN); 9783037854969 (ISBN), Conference Paper, <https://www.scopus.com/inward/record.uri?eid=2-s2.0-84870592534&doi=10.4028%2fwww.scientific.net%2fAMM.214.440&partnerID=40&md5=8795cd4829e3bc7fa5ff1cd5f2d5531b>

<https://www.scientific.net/AMM.214.440>.

Cheng, WM, Nie, W, Zhou, G, Yu, Y, Ma, Y & Xue, J 2012, ‘Research and practice on fluctuation water injection technology at low permeability coal seam’, *Safety Science*, vol. 50, no. 4, pp. 851-6.

Colinet, J, Reed, W & Potts, JD 2013, ‘Impact on respirable dust levels when operating a flooded-bed scrubber in 20-foot cuts’.

Cooper, MR, Susi, P & Rempel, D 2012, ‘Evaluation and control of respirable silica exposure during lateral drilling of concrete’, *J Occup Environ Hyg*, vol. 9, no. 2, pp. D35-41.

Donham, K, Lange, J, Kline, A, Rautiainen, R & Grafft, L 2011, ‘Prevention of Occupational Respiratory Symptoms Among Certified Safe Farm Intervention Participants’, *Journal of Agromedicine*, vol. 16, no. 1, pp. 40-51.

Du, CF, Du, JH & Li, HS 2011, ‘Experiment study on formula and field of the dust suppressant for the open mine road’, in *Advanced Materials Research*, vol. 152, pp. 1847-55.

Du Plessis, J, Janse van Rensburg, L & Jansen van Rensburg, L 2016, ‘Effectiveness of applying dust suppression palliatives on haul roads’.

Echt, AS, Sanderson, WT, Mead, KR, Feng, HA, Farwick, DR & Farwick, DR 2016, ‘Effective dust control systems on concrete dowel drilling machinery’, *J Occup Environ Hyg*, vol. 13, no. 9, pp. 718-24.

Fan, S, Wong, Y-w, Shen, L, Lu, W, Wang, T, Yu, A & Shen, Q 2012, ‘The effectiveness of DustBubbles on dust control in the process of concrete drilling’, *Safety Science*, vol. 50, no. 5, pp. 1284-9.

Fang, C, Sheji, Z, Wei, M & Jinming, M 2019, ‘Research and application of dust control technology based on long pressure and short pumping system in mechanized tunneling face of coal roadway’, in Place of Publication: Taiyuan, China. Country of Publication: UK., vol. 1300, p. 012058.

Firdaussyah, AT & Suryo, T 2018, ‘Evaluation of Control Method Failures for Exposure to Sandblasting Silica Dust in a Steel Construction Company, Indonesia’, *KnE Life Sciences*, pp. 391–400-391–400.

Gao, G, Wang, C & Kou, Z 2018, ‘Experimental studies on the spraying pattern of a swirl nozzle for coal dust control’, *Applied Sciences*, vol. 8, no. 10, p. 1770.

Garcia, A, Jones, E, Echt, AS & Hall, RM 2014, ‘An evaluation of an aftermarket local exhaust ventilation device for suppressing respirable dust and respirable crystalline silica dust from powered saws’, *J Occup Environ Hyg*, vol. 11, no. 11, pp. D200-7.

Ge, S, Huang, Z & Jing, D 2019, ‘RESEARCH AND APPLICATION OF DUST POLLUTION RULE OF MATERIAL PIT AND FOG REDUCTION TECHNOLOGY OF FOGGER’, *Fresenius Environmental Bulletin*, vol. 28, no. 5, pp. 4185-92.

Gottesfeld, P, Tirima, S, Anka, SM, Fotso, A & Nota, MM 2019, ‘Reducing Lead and Silica Dust Exposures in Small-Scale Mining in Northern Nigeria’, *Ann Work Expo Health*, vol. 63, no. 1, pp. 1-8.

Guo, C, Nie, W, Xu, C, Peng, H, Zhang, C, Li, S, Yue, N, Liu, Z, Yang, S & Ma, Q 2020, ‘A study of the spray atomization and suppression of tunnel dust pollution based on a CFD-based simulation’, *Journal of Cleaner Production*, vol. 276, p. 123632.

Guo, Q, Ren, W & Shi, J 2019, ‘Foam for coal dust suppression during underground coal mine tunneling’, *Tunnelling and Underground Space Technology*, vol. 89, pp. 170-8.

Gurley, H, Chugh, Y & Hirschi, J 2010, ‘Field performance of a modified continuous miner for coal and quartz dust control’.

Han, F & Liu, J 2018, ‘Flow field characteristics and coal dust removal performance of an arc fan nozzle used for water spray’, *PLoS One*, vol. 13, no. 9, p. e0203875.

Han, F, Wang, D, Jiang, J & Zhu, X 2014, ‘Modeling the influence of forced ventilation on the dispersion of droplets ejected from roadheader-mounted external sprayer’, *International Journal of Mining Science and Technology*, vol. 24, no. 1, pp. 129-35.

—— 2016, ‘A new design of foam spray nozzle used for precise dust control in underground coal mines’, *International Journal of Mining Science and Technology*, vol. 26, no. 2, pp. 241-6.

He, J-f, Sun, B-x, Liang, Y-p & Luo, Y-j 2018, ‘Research on suction capacity and dust suppression performance of a reverse circulation air hammer in tunnel drilling’, *Tunnelling and Underground Space Technology*, vol. 71, pp. 391-402.

Hedges, K, Reed, S, Mulley, RC, Djukic, W & Tiernan, G 2010, ‘Exposure, health effects and control of respirable crystalline silica in Queensland quarries’, *Journal of Health, Safety and Environment*, pp. 109-21.

Hennessy, KA & Dynan, J 2014, ‘Improving compliance with personal protective equipment use through the model for improvement and staff champions’, *Clin J Oncol Nurs*, vol. 18, no. 5, pp. 497-500.

Hu, G, Xu, J, Ren, T, Dong, Y, Qin, W & Shan, Z 2016, ‘Field investigation of using water injection through inseam gas drainage boreholes to control coal dust from the longwall face during the influence of abutment pressure’, *International Journal of Mining, Reclamation and Environment*, vol. 30, no. 1, pp. 48-63.

Hu, S, Huang, Y, Feng, G, Shao, H, Liao, Q, Gao, Y & Hu, F 2019, ‘Investigation on the design of atomization device for coal dust suppression in underground roadways’, *Process Safety and Environmental Protection*, vol. 129, pp. 230-7.

Hu, Y, Shi, L, Shan, Z, Dai, R & Chen, H 2020, ‘Efficient removal of atmospheric dust by a suppressant made of potato starch, polyacrylic acid and gelatin’, *Environmental Chemistry Letters*, vol. 18, no. 5, pp. 1-11.

Hua, Y, Nie, W, Liu, Q, Peng, H, Wei, W & Cai, P 2020, ‘The development and application of a novel multi-radial-vortex-based ventilation system for dust removal in a fully mechanized tunnelling face’, *Tunnelling and Underground Space Technology*, vol. 98, p. 103253.

Jian, L, Na, H, Yinwen, W & Jin, L 2012, ‘Water jet vacuum dust suppression device used to tunnel development’, *Advanced Materials Research*, vol. 594-597, pp. 1188-92.

Joy, GJ 2012, ‘Evaluation of the approach to respirable quartz exposure control in U.S. coal mines’, *J Occup Environ Hyg*, vol. 9, no. 2, pp. 65-8.

Kanjiyangat, V & Hareendran, M 2018, ‘Coal dust exposure reduction using water mist system: A case study’, *Journal of Chemical Health & Safety*, vol. 25, no. 4, pp. 28-32.

Kokkonen, A, Linnainmaa, M, Säämänen, A, Kanerva, T, Sorvari, J, Kolehmainen, M, Lappalainen, V & Pasanen, P 2019, ‘Control of Dust Dispersion From an Enclosed Renovation Site Into Adjacent Areas by Using Local Exhaust Ventilation’, *Ann Work Expo Health*, vol. 63, no. 4, pp. 468-79.

Kokkonen, A, Linnainmaa, M, Säämänen, A, Lappalainen, V, Kolehmainen, M & Pasanen, P 2017, ‘Evaluation of Real-World Implementation of Partitioning and Negative Pressurization for Preventing the Dispersion of Dust From Renovation Sites’, *Ann Work Expo Health*, vol. 61, no. 6, pp. 681-91.

Li, G, Hu, J, Hao, X & Qu, H 2020, ‘Application and Research of Swirling Curtain Dust Collection Technology in Mines’, *Applied Sciences-Basel*, vol. 10, no. 6.

Li, P, Zhou, Z, Chen, L, Liu, G & Xiao, W 2019, ‘Research on Dust Suppression Technology of Shotcrete Based on New Spray Equipment and Process Optimization’, *Advances in Civil Engineering*, vol. 2019.

Li, S, Xie, B, Hu, S, Jin, H, Liu, H, Tan, X & Zhou, F 2019, ‘Removal of dust produced in the roadway of coal mine using a mining dust filtration system’, *Advanced Powder Technology*, vol. 30, no. 5, pp. 911-9.

Li, S, Zhou, F, Wang, F & Xie, B 2017, ‘Application and research of dry-type filtration dust collection technology in large tunnel construction’, *Advanced Powder Technology*, vol. 28, no. 12, pp. 3213-21.

Li, Y, Li, Y, Li, Y & Fang, L 2020, ‘A Combustible Dust Cleaning Device and Application’, *Earth and Environmental Science* vol. 446, no. 2, p. 022015.

Liao, Q, Feng, G, Fan, Y, Hu, S, Shao, H & Huang, Y 2018, ‘Experimental Investigations and Field Applications of Chemical Suppressants for Dust Control in Coal Mines’, *Advances in Materials Science and Engineering*, vol. 2018, pp. 1-9.

Lin, M-H, Liou, S-H, Chang, C-W, Huang, IH, Strickland, PT & Lai, C-H 2011, ‘An engineering intervention resulting in improvement in lung function and change in urinary 8-hydroxydeoxyguanosine among foundry workers in Taiwan’, *International Archives of Occupational and Environmental Health*, vol. 84, no. 2, pp. 175-83.

Lin, MS, Li, Z & Zhang, HL 2014, ‘The comprehensive management of coal dust and dust removal system development’, in *2013 2nd International Conference on Frontiers of Energy and Environment Engineering, ICFEEE 2013*, Hong Kong, vol. 1, pp. 157-60.

Liu, Q, Nie, W, Hua, Y, Jia, L, Li, C, Ma, H, Wei, C, Liu, C, Zhou, W & Peng, H 2019, ‘A study on the dust control effect of the dust extraction system in TBM construction tunnels based on CFD computer simulation technology’, *Advanced Powder Technology*, vol. 30, no. 10, pp. 2059-75.

Liu, Q, Nie, W, Hua, Y, Peng, H & Liu, Z 2018, ‘The effects of the installation position of a multi-radial swirling air-curtain generator on dust diffusion and pollution rules in a fully-mechanized excavation face: A case study’, *Powder Technology*, vol. 329, pp. 371-85.

Liu, Q, Nie, W, Hua, Y, Peng, H, Ma, H, Yin, S & Guo, L 2019, ‘Long-duct forced and short-duct exhaust ventilation system in tunnels: Formation and dust control analysis of pressure ventilation air curtain’, *Process Safety and Environmental Protection*, vol. 132, pp. 367-77.

Liu, X, Qian, J, Wang, E & Zhang, Z 2020, ‘Study of integrated vortex ventilation and dust removal system in mechanized excavation face’, *Proceedings of the Institution of Mechanical Engineers, Part E: Journal of Process Mechanical Engineering*, vol. 235, no. 1, p. 0954408920936795.

Louk, AK, Patts, JR, Haas, EJ & Cecala, AB 2020, ‘Evaluation of Engineering Controls at Bagging Operations to Reduce Exposures to Respirable Crystalline Silica Dust’, *Mining Metallurgy & Exploration*, vol. 37, no. 4, pp. 1055-64.

Lu, X, Wang, D, Xu, C, Zhu, C & Shen, W 2015, ‘Experimental investigation and field application of foam used for suppressing roadheader cutting hard rock in underground tunneling’, *Tunnelling and Underground Space Technology*, vol. 49, pp. 1-8.

Lu, X, Zhu, H & Wang, D 2017, ‘Investigation on the new design of foaming device used for dust suppression in underground coal mines’, *Powder Technology*, vol. 315, pp. 270-5.

Lu, X-x, Wang, D-m, Zhu, C-b, Shen, W, Zhong, X-x & Xu, C-h 2015, ‘A new adding method of foaming agent used for foam dust suppression in underground coal mines’, *Journal of Central South University*, vol. 22, no. 8, pp. 3116-22.

Lu, X-X, Zhu, H-Q & Wang, D-M 2019, ‘New technology and practice of dust pollution control with foam jet in underground mines’, *Journal of Environmental Science and Health Part a-Toxic/Hazardous Substances & Environmental Engineering*, vol. 54, no. 1, pp. 39-47.

Ma, Q, Nie, W, Yang, S, Xu, C, Peng, H, Liu, Z, Guo, C & Cai, X 2020, ‘Effect of spraying on coal dust diffusion in a coal mine based on a numerical simulation’, *Environ Pollut*, vol. 264, p. 114717.

Middaugh, B, Hubbard, B, Zimmerman, N & McGlothlin, J 2012, ‘Evaluation of Cut-Off Saw Exposure Control Methods for Respirable Dust and Crystalline Silica in Roadway Construction’, *Journal of Occupational and Environmental Hygiene*, vol. 9, no. 3, pp. 157-65.

Morteza, MM, Hossein, K, Amirhossein, M, Naser, H, Gholamhossein, H & Hossein, F 2013, ‘Designing, construction, assessment, and efficiency of local exhaust ventilation in controlling crystalline silica dust and particles, and formaldehyde in a foundry industry plant’, *Arh Hig Rada Toksikol*, vol. 64, no. 1, pp. 123-31.

Nie, W, Liu, Y, Wang, H, Wei, W, Peng, H, Cai, P, Hua, Y & Jin, H 2017, ‘The development and testing of a novel external-spraying injection dedusting device for the heading machine in a fully-mechanized excavation face’, *Process Safety and Environmental Protection*, vol. 109, pp. 716-31.

Nie, W, Liu, Y, Wei, W, Hu, X, Ma, X & Peng, H 2016, ‘Effect of suppressing dust by multi-direction whirling air curtain on fully mechanized mining face’, *International Journal of Mining Science and Technology*, vol. 26, no. 4, pp. 629-35.

Nie, W, Ma, X, Cheng, W, Liu, Y, Xin, L, Peng, H & Wei, W 2016, ‘A novel spraying/negative-pressure secondary dust suppression device used in fully mechanized mining face: A case study’, *Process Safety and Environmental Protection*, vol. 103, pp. 126-35.

Patts, JR, , AB & Haas, EJ 2020, ‘Helmet-CAM: Strategically Minimizing Exposures to Respirable Dust Through Video Exposure Monitoring’, *Mining Metallurgy & Exploration*, vol. 37, no. 2, pp. 727-32.

Peng, H, Cheng, W, Guo, Y, Xu, C, Guo, C, Ma, Q, Liu, Z & Yang, S 2020, ‘Study on the spray field distribution of the roadway full-section water curtain device and its effect on the settlement of PM2.5’, *Process Safety and Environmental Protection*, vol. 143, pp. 101-13.

Peng, H, Nie, W, Cai, P, Liu, Q, Liu, Z & Yang, S 2019, ‘Development of a novel wind-assisted centralized spraying dedusting device for dust suppression in a fully mechanized mining face’, *Environmental Science and Pollution Research*, vol. 26, no. 4, pp. 3292-307.

Peng, H, Nie, W, Liu, Z, Xiu, Z, Yang, S, Xu, C, Ma, Q & Guo, C 2020, ‘Optimization of external spray negative-pressure mist-curtain dust suppression devices for roadheaders based on a multi-factor orthogonal experiment’, *Journal of Cleaner Production*, vol. 275, p. 123603.

Peng, H, Nie, W, Yu, H, Cheng, W, Bai, P, Liu, Q, Liu, Z, Yang, S, Xu, C, Hua, Y, Guo, C & Ma, Q 2019, ‘Research on mine dust suppression by spraying: Development of an air-assisted PM10 control device based on CFD technology’, *Advanced Powder Technology*, vol. 30, no. 11, pp. 2588-99.

Potts, JD & Reed, WR 2011, ‘Field evaluation of air-blocking shelf for dust control on blasthole drills’, *International Journal of Mining, Reclamation and Environment*, vol. 25, no. 1, pp. 32-40.

Pounds, L, Duysen, E, Romberger, D, Cramer, ME, Wendl, M & Rautiainen, R 2014, ‘Social Marketing Campaign Promoting the Use of Respiratory Protection Devices Among Farmers’, *Journal of Agromedicine*, vol. 19, no. 3, pp. 316-24.

Qi, C & LO, L-M 2016, ‘Engineering Control of Silica Dust from Stone Countertop Fabrication and Installation’, *CDC, NIOSH, March*.

Reed, WR, Klima, S, Shahan, M, Ross, GJH, Singh, K, Cross, R & Grounds, T 2019, ‘A field study of a roof bolter canopy air curtain (2nd generation) for respirable coal mine dust control’, *Int J Min Sci Technol*, vol. 29, no. 5, pp. 711-20.

Reed, WR, Shahan, M, Klima, S, Ross, G, Singh, K, Cross, R & Grounds, T 2020, ‘Field study results of a 3rd generation roof bolter canopy air curtain for respirable coal mine dust control’, *International journal of coal science & technology*, vol. 7, no. 1, pp. 79-87.

Reed, WR, Shahan, M, Ross, G, Blackwell, D & Peters, S 2020, ‘Field comparison of a roof bolter dry dust collection system with an original designed wet collection system for dust control’, *Mining Metallurgy & Exploration*, vol. 37, pp. 1885-98.

Ren, T, Karekal, S, Cooper, G, Wang, Z & Plush, B 2013, ‘Design and field trials of water-mist based venturi systems for dust mitigation on longwall faces’.

Ren, T, Wang, Z & Cooper, G 2014, ‘CFD modelling of ventilation and dust flow behaviour above an underground bin and the design of an innovative dust mitigation system’, *Tunnelling and Underground Space Technology*, vol. 41, pp. 241-54.

Ren, W, Shi, J, Zhu, J & Guo, Q 2020, ‘An innovative dust suppression device used in underground tunneling’, *Tunnelling and Underground Space Technology*, vol. 99, p. 103337.

Ren, W, Wang, D, Guo, Q & Zuo, B 2014, ‘Application of foam technology for dust control in underground coal mine’, *International Journal of Mining Science and Technology*, vol. 24, no. 1, pp. 13-6.

Ren, XW, Wang, MD, Kang, HZ & Lu, XX 2012, ‘Engineering case report: A new method for reducing the prevalence of pneumoconiosis among coal miners: Foam technology for dust control’, *Journal of Occupational and Environmental Hygiene*, vol. 9, no. 4, pp. D77-D83.

Roberts, J & Wypych, P 2017, ‘Research into Improving the Efficiency of Water Spraying Airborne Dust Control Techniques in the Iron Ore Industry’, *Iron Ore Conference*.

Robertsen, O, Hegseth, MN, Foreland, S, Siebler, F, Eisemann, M & Vangberg, HCB 2020, ‘The Effect of a Knowledge-Based Intervention on the Use of Respirators in the Norwegian Smelter Industry’, *Frontiers in Psychology*, vol. 11, p. 270.

Seixas, N, Neitzel, R, Stover, B, Sheppard, L, Daniell, B, J, E & H, M 2011, ‘A multi-component intervention to promote hearing protector use among construction workers’, *International Journal of Audiology*, vol. 50, pp. S46-S56.

Shamsi, M, Pariani, A, Shams, M & Soleymani-nejad, M 2016, ‘Persuasion to use personal protective equipment in constructing subway stations: application of social marketing’, *Injury Prevention*, vol. 22, no. 2, pp. 149-52.

Shang, JR 2014, ‘Study on the Application Results of" Dry Cleaning" Mode in Port Dust Control’, in *Applied Mechanics and Materials*, vol. 448, pp. 4358-64.

Shepherd, S & Woskie, S 2013, ‘Controlling Dust from Concrete Saw Cutting’, *Journal of Occupational and Environmental Hygiene*, vol. 10, no. 2, p. 64.

Shi, L, Yang, CW, Yu, XM & Luo, XW 2013, *Development of the tunnel wet dust precipitator and testing*, Zhangjia Jie, 10226680 (ISSN); 9783037858509 (ISBN), Conference Paper, <https://www.scopus.com/inward/record.uri?eid=2-s2.0-84886299968&doi=10.4028%2fwww.scientific.net%2fAMR.800.8&partnerID=40&md5=aeb4060ec1510baacf9cf84eabf1cf81>

<https://www.scientific.net/AMR.800.8>.

Snipes, SA, Smyth, JM, Murphy, D, Miranda, PY & Ishino, FA 2015, ‘Provision Increases Reported PPE Use for Mexican Immigrant Farmworkers: An mHealth Pilot Study’, *J Occup Environ Med*, vol. 57, no. 12, pp. 1343-6.

Summers, MP & Parmigiani, JP 2015, ‘A Water Soluble Additive to Suppress Respirable Dust from Concrete-Cutting Chainsaws: A Case Study’, *Journal of Occupational and Environmental Hygiene*, vol. 12, no. 4, pp. D29-D34.

Sun, B, Cheng, W, Wang, J & Wang, H 2018, ‘Effects of turbulent airflow from coal cutting on pollution characteristics of coal dust in fully-mechanized mining face: A case study’, *Journal of Cleaner Production*, vol. 201, pp. 308-24.

Sun, B, Cheng, W, Wang, J, Wang, H & Ma, Y 2019, ‘Development of Venturi negative-pressure secondary dedust device and application of local spray closure technique’, *Advanced Powder Technology*, vol. 30, no. 1, pp. 42-54.

Tovar-Aguilar, JA, Monaghan, PF, Bryant, CA, Esposito, A, Wade, M, Ruiz, O & McDermott, RJ 2014, ‘Improving Eye Safety in Citrus Harvest Crews Through the Acceptance of Personal Protective Equipment, Community-Based Participatory Research, Social Marketing, and Community Health Workers’, *Journal of Agromedicine*, vol. 19, no. 2, pp. 107-16.

Wallace, KA & Cheung, WM 2013, ‘Development of a compact excavator mounted dust suppression system’, *Journal of Cleaner Production*, vol. 54, pp. 344-52.

Wang, D, Lu, X, Wang, H & Chen, M 2016, ‘A new design of foaming agent mixing device for a pneumatic foaming system used for mine dust suppression’, *International Journal of Mining Science and Technology*, vol. 26, no. 2, pp. 187-92.

Wang, H, Wang, D, Lu, X, Gao, Q, Ren, W & Zhang, Y 2012, ‘Experimental investigations on the performance of a new design of foaming agent adding device used for dust control in underground coal mines’, *Journal of Loss Prevention in the Process Industries*, vol. 25, no. 6, pp. 1075-84.

Wang, H, Wang, D, Ren, W, Lu, X, Han, F & Zhang, Y 2013, ‘Application of foam to suppress rock dust in a large cross-section rock roadway driven with roadheader’, *Advanced Powder Technology*, vol. 24, no. 1, pp. 257-62.

Wang, H, Wang, D, Wang, Q & Jia, Z 2014, ‘Novel Approach for Suppressing Cutting Dust Using Foam on a Fully Mechanized Face with Hard Parting’, *Journal of Occupational and Environmental Hygiene*, vol. 11, no. 3, pp. 154-64.

Wang, HT, Wang, DM & Ren, WX 2011, ‘A new technique for preparation of two-phase foam materials for controlling mine dust and its application’, *Advanced Materials Research*, vol. 328-330, pp. 372-5.

Wang, J, Zhou, G, Wei, X & Wang, S 2019, ‘Experimental characterization of multi-nozzle atomization interference for dust reduction between hydraulic supports at a fully mechanized coal mining face’, *Environ Sci Pollut Res Int*, vol. 26, no. 10, pp. 10023-36.

Wang, K, Ding, C, Jiang, S, Zhengyan, W, Shao, H & Zhang, W 2019, ‘Application of the addition of ionic liquids using a complex wetting agent to enhance dust control efficiency during coal mining’, *Process Safety and Environmental Protection*, vol. 122, pp. 13-22.

Wang, K, Ma, X, Jiang, S, Wu, Z, Shao, H & Pei, X 2016, ‘Application study on complex wetting agent for dust-proof after gas drainage by outburst seams in coal mines’, *International Journal of Mining Science and Technology*, vol. 26, no. 4, pp. 669-75.

Wang, Q, Wang, D, Han, F, Yang, F & Sheng, Y 2020, ‘Study and application on foam-water mist integrated dust control technology in fully mechanized excavation face’, *Process Safety and Environmental Protection*, vol. 133, pp. 41-50.

Wang, Q, Wang, D, Wang, H, Han, F, Zhu, X, Tang, Y & Si, W 2015, ‘Optimization and implementation of a foam system to suppress dust in coal mine excavation face’, *Process Safety and Environmental Protection*, vol. 96, pp. 184-90.

Wang, Q, Wang, D, Wang, H, Liu, J & He, F 2016, ‘Experimental study and implementation of a novel internal foam spraying system for roadheaders’, *Tunnelling and Underground Space Technology*, vol. 59, pp. 127-33.

Wang, Q, Wang, D, Wang, H, Shen, Y & Zhu, X 2018, ‘Experimental investigations of a new surfactant adding device used for mine dust control’, *Powder Technology*, vol. 327, pp. 303-9.

Wang, X, Yuan, S, Li, X & Jiang, B 2019, ‘Synergistic effect of surfactant compounding on improving dust suppression in a coal mine in Erdos, China’, *Powder Technology*, vol. 344, pp. 561-9.

Wang, Y, Jiang, Z, Chen, J, Chen, J & Wang, M 2019, ‘Study of high-pressure air curtain and combined dedusting of gas water spray in multilevel ore pass based on CFD-DEM’, *Advanced Powder Technology*, vol. 30, no. 9, pp. 1789-804.

Warden, TW & Warden, CM 2019, ‘Tunnel dust control project’, in D Peila, G Viggiani, G Viggiani & T Celestino (eds), *World Tunnel Congress, WTC 2019 and the 45th General Assembly of the International Tunnelling and Underground Space Association, ITA-AITES 2019*, pp. 557-64.

Weidman, J, Dickerson, DE & Koebel, CT 2016, ‘Effective Intervention Strategy to Improve Worker Readiness to Adopt Ventilated Tools’, *Journal of Construction Engineering and Management*, vol. 142, no. 8.

Woith, WM, Bykova, A & Abdulrehman, M 2015, ‘Feasibility of a Photovoice Study Promoting Respirator Use among Russian Health Care Workers’, *Public Health Nursing*, vol. 32, no. 5, pp. 471-7.

Xia, B, Liao, R, Grima, A, Pan, R & Wypych, P 2016, ‘Dust control technology for coal handling and processing: Crushing station case study’, in *12th International Conference on Bulk Materials Storage, Handling and Transportation (ICBMH 2016), The*, p. 73.

Xie, J, Xue, S, Chen, W & Zhou, G 2012, ‘An enclosed dust removal system with ducting’, *AGH Journal of Mining and Geoengineering*, vol. 36, no. 3, pp. 423-33.

Xu, C, Nie, W, Liu, Z, Peng, H, Yang, S & Liu, Q 2019, ‘Multi-factor numerical simulation study on spray dust suppression device in coal mining process’, *Energy*, vol. 182, pp. 544-58.

Xu, C, Nie, W, Yang, S, Peng, H, Liu, Z, Ma, Q, Guo, C & Liu, Q 2020, ‘Numerical simulation of the multi-index orthogonal experiments on the spray dust-settling devices’, *Powder Technology*, vol. 371, pp. 217-30.

Yang, S, Nie, W, Lv, S, Li, Z, Peng, H, Ma, X, Cai, P & Xu, C 2019, ‘Effects of spraying pressure and installation angle of nozzles on atomization characteristics of external spraying system at a fully-mechanized mining face’, *Powder Technology*, vol. 343, pp. 754-64.

Yin, Q, Peng, J, Bo, K, He, J, Kui, Y & Gan, X 2013, ‘Study on dust control performance of a hammer drill bit’, *International Journal of Mining, Reclamation and Environment*, vol. 27, no. 6, pp. 393-406.

Yin, S, Nie, W, Liu, Q & Hua, Y 2019, ‘Transient CFD modelling of space-time evolution of dust pollutants and air-curtain generator position during tunneling’, *Journal of Cleaner Production*, vol. 239.

Zarei, A, Jahangiri, M, Koohpaei, A, Zolfaghari, A, Barkhordari, A & Mortezavi Mehrizi, M 2018, ‘Design, Construction and Evaluation of Local Exhaust Ventilation System for the Control of Total Dust and Crystalline Silica in a Tile Manufacturing Factory’, *Journal of Health Sciences and Surveillance System*, vol. 6, no. 4, pp. 165-72.

Zhang, YF, Xiao, Q, Zhao, M, Guo, WW & Zhang, L 2014, *Study on dust treatment process of stone processing*, Trans Tech Publications Ltd, 10226680 (ISSN); 9783038352488 (ISBN), Conference Paper, <https://www.scopus.com/inward/record.uri?eid=2-s2.0-84913597885&doi=10.4028%2fwww.scientific.net%2fAMR.1030-1032.322&partnerID=40&md5=2c49e65d65546463aa736136b664d1de>

<https://www.scientific.net/AMR.1030-1032.322>.

Zhou, G, Feng, B, Yin, W & Wang, J 2018, ‘Numerical simulations on airflow-dust diffusion rules with the use of coal cutter dust removal fans and related engineering applications in a fully-mechanized coal mining face’, *Powder Technology*, vol. 339, pp. 354-67.

Zhou, G, Nie, W, Cheng, W, Wang, D, Yu, Y & Yang, J 2012, ‘Dust Simulation and Application of Forced and Exhausted Mixed Ventilation System in Half Coal-rock Fully-mechanized Excavation Face’, in J Wu, J Yang, N Nakagoshi, X Lu & H Xu (eds), *Natural Resources and Sustainable Development Ii, Pts 1-4*, vol. 524-527, pp. 285-+.

Zhou, G, Wang, D, Cheng, W, Pan, G & Cao, S 2012, ‘Research of Ventilation and Dust Removal Technology for Whole Rock Fully-mechanized Excavation Face’, in WZ Chen, Q Li, YL Chen, PQ Dai & ZY Jiang (eds), *New Materials and Processes, Pts 1-3*, vol. 476-478, pp. 1297-+.

Zhou, G, Wang, DM, Cheng, WM & Cao, S 2013, *Numerical simulation research on gas-dust flow field of forced-exhausted hybrid ventilation in whole rock mechanized heading face*, Guilin, 16609336 (ISSN); 9783037857519 (ISBN), Conference Paper, <https://www.scopus.com/inward/record.uri?eid=2-s2.0-84882971190&doi=10.4028%2fwww.scientific.net%2fAMM.336-338.873&partnerID=40&md5=cbec9bfd5f80a3bdf9ace1d771d43349>

<https://www.scientific.net/AMM.336-338.873.pdf>.

Zhou, G, Xu, M, Qiu, H, Nie, W, Cheng, W & Chen, C 2017, ‘Experimental investigation about the influence of airflow on droplet sizes of mechanical nozzles for coal mining face’, *Tehnički vjesnik*, vol. 24, no. 6, pp. 1713-21.

Zhou, G, Zhang, Q, Bai, R, Fan, T & Wang, G 2017, ‘The diffusion behavior law of respirable dust at fully mechanized caving face in coal mine: CFD numerical simulation and engineering application’, *Process Safety and Environmental Protection*, vol. 106, pp. 117-28.

Zhou, G, Zhang, Q, Hu, Y, Gao, D, Wang, S & Sun, B 2020, ‘Dust removal effect of negatively-pressured spraying collector for advancing support in fully mechanized coal mining face: Numerical simulation and engineering application’, *Tunnelling and Underground Space Technology*, vol. 95.

Zhou, Q, Qin, B, Ma, D & Jiang, N 2017, ‘Novel technology for synergetic dust suppression using surfactant-magnetized water in underground coal mines’, *Process Safety and Environmental Protection*, vol. 109, pp. 631-8.

Zhou, Q, Qin, B, Wang, F, Wang, H, Hou, J & Wang, Z 2019, ‘Effects of droplet formation patterns on the atomization characteristics of a dust removal spray in a coal cutter’, *Powder Technology*, vol. 344, pp. 570-80.

Zhou, Q, Qin, B, Wang, J, Wang, H & Wang, F 2018, ‘Effects of preparation parameters on the wetting features of surfactant-magnetized water for dust control in Luwa mine, China’, *Powder Technology*, vol. 326, pp. 7-15.

Zhou, W, Nie, W, Liu, X, Zhou, C, Wei, C, Liu, C, Liu, Q & Yin, S 2020, ‘Optimization of dust removal performance of ventilation system in tunnel constructed using shield tunneling machine’, *Building and Environment*, vol. 173, p. 106745.

Zhu, X, Wang, H, Wang, D, Xu, C, Zhou, W & Zhu, Y 2020, ‘Improved foam application at the tunnel face with large ventilation volume and low pressure supplied water’, *Tunnelling and Underground Space Technology*, vol. 95.

Zongyin, D 2013, ‘Research on dust suppression of a copper mine underground middle level crushing station’, *Advanced Materials Research*, vol. 726-731, pp. 907-12.
