# Supplementary material for: A systematic review of the effectiveness of dust control measures adopted to reduce workplace exposure
Source: Environ Sci Pollut Res Int. 2023 Mar 25;30(19):54407–28. doi: 10.1007/s11356-023-26321-w (PMC10121514; doi:10.1007/s11356-023-26321-w)
Supplement: Supplementary file 3 — Supplementary file3 (DOCX 193 KB) [file 11356_2023_26321_MOESM3_ESM.docx]

**SUPPLEMENTARY SHEET 3**

A systematic review of the effectiveness of dust control measures adopted to reduce respirable crystalline silica exposure in the workplace

**Dust control methods and dust sampling information**

| **Mode of Dust Control** | **Dust Control Intervention** | **Study** | **Workplace** | **Main equipment targeted** | **Dust sampler or sampling method** | **Sampling Points** | **Sampling Height** |
| --- | --- | --- | --- | --- | --- | --- | --- |
| Legislative | Regulatory | (Joy 2012) | Coal mines | Not stated | Not stated | Not stated | Not stated |
| Foam Technology | Foaming agent | (Guo et al. 2019) | Coal tunnelling face | Road header | AKFC-92A | Road header’s driver position 5m behind the driver’s position 10m behind the driver’s position | Not stated |
|  | Foaming generating device | (Wang, D et al. 2016) | Coal mining face | Road header | Not stated | The driver’s position | Not stated |
|  | Internal foam-spraying system | (Wang, Q et al. 2016) | Coal mining face | Longitudinal road header | Not stated | Driver’s position | Not stated |
|  | Foaming generating device | (Lu et al. 2017) | Coal mine roadway | An EBZ160 type road header | A charge coupled device (CCD), CCD-1000 direct-reading dust-measuring instrument | The driver’s position 5 m behind the road header | 1.7m |
|  | Foaming spray system | (Lu, X et al. 2015) | Coal mining face | An EBZ-200H road header | The CCF-7000 direct-reading dust-measuring instrument. | The driver’s position  5 m behind the road header | 1.7m |
|  | Foaming agent | (Chen et al. 2015) | Coal mining face | Not stated | Not stated | Not stated | Not stated |
|  | Foaming agent adding device | (Lu, X-x et al. 2015) | Coal mining face | An EBZ132 type road header | CCGZ-1000 direct-reading dust-measuring instrument | At the driver’s position 5 m behind the road header – opposite to the side with the ventilating duct | Not stated |
|  | Foaming generating device | (Wang et al. 2015) | Coal mine roadway | Longitudinal road header | Not stated | Not stated | Not stated |
|  | Foam spray system | (Ren, W et al. 2014) | Coal mining face | Fully mechanized digging machine | The light scattering digital conimeter | Three measuring points near the digging machine. | Not stated |
|  | Foaming agent adding device | (Wang et al. 2012) | Coal mining face | Road header | Not stated | Two points beside the driver | Not stated |
|  | Foaming spray system | (Wang et al. 2011) | Coal mining face | Excavating machine | Not stated | The excavating machine driver site.  The return air side behind the excavating machine. | Not stated |
|  | Foaming spray system | (Ren et al. 2012) | Coal mining face | Fully mechanized coal mining machine | The P-5L 2 light scattering digital conimeter | Three points near the mining machine and in the return-airway. | Not stated |
|  | Foam generating device | (Wang et al. 2013) | Coal mining face | Road header | The CCGZ-1000 direct-reading dust-measuring instrument | Two dust-measuring locations were arranged beside the driver’s position | 1.7 m |
|  | Foam generator | (Wang et al. 2014) | Coal mining face | A MG550/1380-WD shearer with double drums | A CCGZ-1000 direct-reading dust monitor  AZF-01 respirable dust samplers | Near the shearer driver  Downwind at 15 m from the shearer. | Not stated |
|  | Foam spray nozzle | (Han et al. 2016) | Coal roadway | EBZ160 road header | Not stated | Around the driver’s position | Not stated |
|  | Foam spray system | (Zhu et al. 2020) | Tunnelling face | An EBZ160 road header | AZF-01 respirable dust samplers.  CCZ-1000 dust monitor. | At the driver’s position.  The wind return side of the tunnelling machine. | Not stated |
|  | Foaming agent adding device | (Lu et al. 2019) | Coal Mining Face | EBH-120 horizontal road header | Not stated | Road headers driver position | Not stated |
| Surfactant | Surfactant | (Wang, X et al. 2019) | Coal mining face | Shearer | AKFC-92A type dust sampler | Not stated. | Not stated |
|  | Surfactant adding device | (Wang et al. 2018) | Coal excavation face | An EBZ200 longitudinal road header | Not stated | Not stated | Not stated |
|  | Surfactant-magnetized water | (Zhou, Q et al. 2018) | Coal roadway | Not stated | AKFC92A-type dust sampler | Not stated | Not stated |
|  | Surfactant | (Du et al. 2011) | Haul road on an open pit iron ore mine | N/A | The P5 photoelectric dust determinator. | On the road haul roads. | Not stated |
|  | Surfactant | (Du Plessis et al. 2016) | Haul road on an open pit iron ore mine | N/A | A Thermo Scientific personal DataRAM (pDR) monitor | Designated fixed location for each test section every hour over the study period. | 1 m from the ground surface |
|  | Surfactant-magnetized water | (Zhou, Q et al. 2017) | Coal mining face | Not stated | Not stated | Not stated | Not stated |
|  | Surfactant | (Liao et al. 2018) | Coal roadway | Not stated | Not stated | 1m in front and behind the water curtain.  Each point was 2.5m away from the other and 1.25m from the nearest wall. | 1.5 m |
|  | Surfactant | (Zhou et al. 2019) | Coal mining face | coal cutter | AKFC92A-type dust sampler | Two novel nozzle distribution devices installed on the rocker arm of the coal cutter | Not stated |
|  | Surfactant and spray nozzle | (Wang, K et al. 2019) | Coal roadway | Road header | AZF 02 dust sampler | Driver’s Location 10 m from the driver’s rear 50 m from the driver’s rear 100 m from the driver’s rear | Not stated |
|  | Surfactant | (Bao et al. 2020) | Coal mining face | Shearer | TSI9306 handheld laser particle counter | At the position of shearer  The position of shifting frame  At 10 m, 20 m from shearer  The position of reproduction point | 1.5 m |
|  | Surfactant | (Cheng et al. 2020) | Coal storage area in a coal transportation port | Not stated | Not stated | Not stated | Not stated |
|  | Surfactant | (Summers & Parmigiani 2015) | Construction - covered outdoor laboratory | Concrete-cutting chainsaw | SKC AirChek52 sampling pump.  Aluminum cyclones to capture only the respirable fraction of dust (1–10 μm). | Respirable dust samples were collected near each of the four wall corners and on the operator’s lapel | Breathing zone of employees |
|  | Surfactant | (Hu et al. 2020) | Construction site | Not stated | JCH-6120 air sampler | Not stated | Not stated |
| Air currents | Air Curtain | (Zhou, W et al. 2020) | Tunnelling | Shield tunneling machine | TSL-9306 handheld laser particle counter | Dust is sampled at 10, 15, 20, 25, 30, and 35m away from the tunnel face | The operators’ respiratory zone |
|  | Air curtain | (Liu, Nie, Hua, Peng, et al. 2019) | Tunnelling | MH620 type tunneller | AKFC-92A dust sampler | At 2.5, 5.5, 10, 20, 30 and 35m away from the tunnel face.  Three points are selected in each cross section. | 1.55 m  2.65 m |
|  | Air curtain generator | (Cheng, Nie, Zhou, Yang, et al. 2012) | Coal mine roadway | Road header | Not stated | Tunnelling place Driver’s position Downwind of loader Downwind of telescopic belt 100m AND 200M away from the tunnelling place. | Not stated |
|  | Air curtain generator | (Yin et al. 2019) | Coal mine roadway | EBZ-160 Cantilever tunnel boring machine | The AKFC-92A dust concentration tester | 5, 10, 15, 30, and 45m from the cutting face. | 1.55 m |
|  | Air curtain generator | (Fang et al. 2019) | Coal mine roadway | EBZ-220 cantilever roadheader | CCZ-20 sampler Filter membrane | Driver's position 5m away from tail | 2.3 m |
|  | Air curtain | (Reed et al. 2019) | Coal mining areas | Roof bolter | CMDPSU gravimetric sampler. pDR-1000 instantaneous sampler. | Rear of the bolter  Return airway Infront and at the back of the roof bolter operator. In front, middle and rear of the roof bolter machine. | At the lapels of the vest worn by the operator and at his back. |
|  | Air curtain | (Reed, Shahan, Klima, et al. 2020) | Coal mining areas | Roof bolter | pDR- 1000 instantaneous sampler. gravimetric personal sampler. | At the entrance and exit of the line curtain into the roof bolting sections.  The return of the roof bolting section.  Behind the canopy and rear of the centre of the roof bolter.  Directly underneath the plenum outflow for each side of the roof bolter. | Fitted on the operator |
|  | Air curtain generator | (Hua et al. 2020) | Tunnelling Face | The EBZ220-type tunneller | Not stated | At 2, 5, 10, 20 and 30m away from the tunnelling face 0.4m away from the tunnel wall at the blower duct side.  At 2, 5, 10, 20 and 30m away from the tunnelling face 0.4m away from the tunnel wall at the exhaust duct side.  At the mining operation location, 5m from the tunnelling face | 1.55 m.  and 2.15 from the tunnel floor. |
|  | Air curtain | (Cai et al. 2020) | Coal mining face | Shearer | Not stated | Advancing support Shearer driver’s area 10, 50 and 100m at the downwind side of the front roller. Air return corner At the crusher. Transfer point between stage loader and belt conveyor transfer point 10 m from air outlet. | At the height of the footway's respiratory zone |
|  | Air curtain generator | (Nie, Liu, et al. 2016) | Coal mining face | Not stated | CCHZ-1000 full-automatic dust monitor. | 5 m and 10 m from the heading face. | 1.55m |
|  | Air curtain generator and wet dust extractor | (Liu et al. 2018) | Coal excavation face | EBZ230 road header | AKFC-92A mine dust sampler. | At 5m, 10m, 15m, 20m, 25m, 30m, 35m, 40m and 45m. | Respiratory zones |
|  | Air curtain  Turbulator and an exhaust air outlet (SEAO) | (Chen & Liu 2019) | Coal mine roadway | Not stated | Not stated | 3, 7, 9, 25 30 and 35m away from the heading face. | 1.55 m |
|  | Air curtain | (Zhou, Wang, et al. 2012) | Coal mining face | Hard rock digging machine | Not stated | Not stated | Not stated |
| Dry extraction system | Dust extraction drill | (He et al. 2018) | Tunnelling in a hydropower station construction | The intervention is the drill bit | No monitoring of dust | N/A | N/A |
|  | Dust extraction during open pit drilling | (Potts & Reed 2011) | Open pit limestone and coal mines | A Reich C-650-C blasthole drill. Atlas-Copco DM45E blasthole drill. | Thermo Electron model 1000 personal data rams (PDRs).  Gravimetric samplers | The environment around the drilling machine. | Not stated |
|  | Baghouse dust collector | (Alexander et al. 2018) | Sand mine | A NOV APPCO FS-30 frac-sander (sand mover) | SKC sampling pumps. | At the four corners and two in the middle of the FS-30. At the head region of the sand mover, one in the middle region and the another at the tail end. This is repeated at the opposite side. | Breathing zone of employees |
|  | Baghouse dust collector (Bag house | (Alexander et al. 2016) | Sand mine | A NOV APPCO FS-30 frac-sander (sand mover) | SKC® XR 5000® personal sampling pumps. | At the four corners and towards the middle of the FS-30.  Around each corner of the FS-30 on the ground and one at the middle on either side. | Breathing zone of employees |
|  | Bag filter | (Zhang et al. 2014) | Stone processing workshop | Not stated | Not stated | Not stated | Not stated |
|  | Dust collector | (Liu, Nie, Hua, Jia, et al. 2019) | Tunnelling | Tunnel boring machine (TBM) | TSI9306 Holding Laser Particle Counter | At 15, 20, 30, 40, 50, 60, 70, 80, 90, and 100m away from the tunnel’s face. | 1.55 m |
|  | Baghouse dust collector | (Li et al. 2017) | Tunnelling | Not stated | CCZ-1000 direct-reading instrument | 1m in front of the dust exhaust hood.  15 m behind the dust collector | 1 m |
|  | Dust isolation and extraction | (Kokkonen et al. 2019) | Construction- renovation Sites | Construction tools that can generate dust | IOM samplers with MultiDust foam   Split2 direct reading dust monitors. | About 1−2 meters from the dust-generating activity | Not stated |
|  | Local exhaust ventilation (LEV) | (Echt et al. 2016) | Construction - outdoor testing area | Two dowel drilling machines (rotary-type pneumatic rock drills). | Polyvinyl chloride filters in three-piece cassettes.  Higgins-Dewell type respirable dust cyclones (Model BGI-4L. | In front of the dowel drilling machine.  At the side of the machine near the control panel.  At the rear of the machine adjacent to the dust collector. | 1.5 m |
|  | LEV | (Garcia et al. 2014) | Construction site | A 7–1/4-inch worm-drive electric circular saw | 37-millimeter (mm) diameter Polyvinyl chloride (PVC) filters.  An active sampling portable laser photometer. | The three sample locations were not identified in the report | Not stated |
|  | Room dedusting system | (Lin et al. 2014) | Laboratory sampling room | Not stated | Not stated | Not stated | Not stated |
|  | Dust extraction drill | (Yin et al. 2013) | Open pit molybdenum ore mine | Reverse circulation DTH air hammer drilling system with a CD-3 coring drill rig | Not stated | Not stated | Not stated |
|  | Dust extraction | (Li, S et al. 2019) | Coal mine roadway | EBZ-318 type road header | CCZ-1000 direct-reading instrument | Inlet of the dust collector -12 m from the heading face. Outlet of the extraction fan - 24 m from the heading face. | 1.5 m |
|  | LEV with wet multi-scrubber | (Zarei et al. 2018) | Tile manufacturing plant | Dust sources in the plant | Model SKC sampling pump. | Not stated | Not stated |
|  | Disposable dust collector | (Fan et al. 2012) | Construction - field laboratory (indoor area) | Electric hammers for small-size holes (Bosch GBH 2-20 D Professional) | GilAir-3 pumps connected to 10 mm nylon cyclones | Personal samplers carried by workers | Breathing zone of employees. |
|  | LEV on a jig | (Cooper et al. 2012) | Construction - training site | APT Model 137 | GilAir-5, Sensidyne, Inc., Clearwater, Fla. | Personal air samples were collected | Breathing zone of employees |
|  | LEV | (Morteza et al. 2013) | Foundry | Various equipment’s at a foundry | PVC membrane filer with a diameter of 37 mm and a pore size of 5 μm using an HD cyclone. Method No.7601of NIOSH. Individual pump and an SKC micro pump. | Not stated | Breathing zone of employees |
|  | Isolation and dust extraction | (Hedges et al. 2010) | Quarries | Excavator saw (350 LCH Hitachi) | AM510 TSI dust analysers DRX TSI dust analyser (Model 8533) | In the excavator cabin. | Not stated |
|  | Dust isolation and extraction | (Kokkonen et al. 2017) | Construction – renovation site | Construction tools that can generate dust | DustTrak 8533 (TSI Inc.) | As close as possible to the dust-generating activity in the work area.  In front of the main entry into the enclosure. | Not stated |
| Water based dust control | Water infusion | (Cheng, Nie, Zhou, Yu, et al. 2012) | Coal mining face | N/A | Not stated | The driver’s position of the coal-winning machine. 5-10m on the leeward side of the coal-winning machine. The moving framework place The putting coal mouth place.  The back and front place of sneaking head. The leeward 5 m place of the crusher The leeward 5–10 m place of the reproduced machine. | Not stated |
|  | Water infusion | (Hu et al. 2016) | Coal mining face | N/A | Not stated | 15m downwind side of the shearer. 15m downwind side of the support. | Not stated |
| Water based dust control | Water curtain | (Hu et al. 2019) | Coal mine roadway | Not targeted at an equipment | The AFKC-92A dust-measuring instrument | Three sample points each 1m away from the other and located in front of the atomization device.  Three sample points each 1m away from the other and located behind the atomization device | 1.7 m above the ground |
|  | Water curtain | (Peng, H. et al. 2020) | Coal mine - return airway | Not targeted at an equipment | TSI9306 particle counter | 10, 20, 50 and 150m away from the down-wind side of the water curtain device. For the multiple set of air curtains monitoring was at 200m away from the coal mining face in the return airway. | 1.6 m |
|  | Water curtain | (Sun et al. 2018) | Coal mining face | Shearer and hydraulic support | Not stated | Hydraulic support advancing workers area.  Position of the front and rear drum drivers. In the centre of shearer.  Where coal fell from cutting process. At 20m and 30m on the lee side of the coal mining machine. | Not stated |
|  | Water curtain | (Zhang et al. 2012) | Coal mining face | Not stated | Not stated | 10 m far away the return airway | Not stated |
| Water based dust control | Water misting | (Wallace & Cheung 2013) | Construction - demolition (test area) | A mini excavator with hydraulic breaker | DustMate dust monitor | Not stated | Not stated |
|  | Water misting | (Xu et al. 2020) | Coal hydraulic support and mining face | Mining machine | The explosion-proof dust samplers | The rest area in the tunnel. The support moving worker's area The mining machine driver's area The crushing plant The reversed loader | The height of the workers' breathing zone |
|  | Coal cutter external misting system | (Ma et al. 2020) | Mechanized coal working face | The coal cutter and the hydraulic supports. | AKFC-92A mine-dust sampler | Coal cutter driver’s position.  Where the advancing-support workers operated.  At 15 m from the coal cutter on the leeward side. | The height of the respiratory zone (1.6m) |
|  | Water misting | (Guo et al. 2020) | Coal mining face | A continuous miner (CM) | A CCZ-1000 direct-reading dust meter | At the tail of the scraper.  At the CM driver’s operating position. | Not stated |
|  | Water misting | (Gottesfeld et al. 2019) | Artisanal small scale open pit gold mining | Crusher or mechanical ore processing machines | Aluminium cyclone (SKC) as per NIOSH method 7500 | On selected miners | Breathing zone of employees |
|  | Water misting | (Kanjiyangat & Hareendran 2018) | Coal fired boiler | All equipment’s in the work area | APM 801 (Envirotech) | Near the breathing zone of worker | Breathing zone of employees |
|  | Water misting | (Ge et al. 2019) | Coal preparation plant | Dumping truck and dump bin | Not stated | At 0, 5, 7.5, 10, 12.5 and 15m from the receiving pits | Not stated |
|  | Water misting | (Xu et al. 2019) | Coal mining face | Shearer | AKFC-92A dust detector | The shearer driver’s position.  10m downwind side of the shearer.  Hydraulic support advancing workers area.  The place with multiple processes. Location of the crusher.  Location of the reversed loader. | Not stated |
|  | Water misting | (Yang et al. 2019) | Coal mining face | Electrical traction coal cutter | AKFC-92A dust sampler | Around the coal cutter driver. | Not stated |
|  | Water misting | (Nie et al. 2017) | Coal excavation face | A heading machine (EBZ-160A) | AKFC-92A dust sampler | At the position of the cutting head.  At the machine’s driver position.  Near the reversed loader.  50 and 100m away from the cutting head | Not stated |
|  | Water misting | (Jian et al. 2012) | Subway heading face | Tunnel digging Machine | Not stated | 5, 10, 50 and 100m away from the tunnelling face. | Not stated |
|  | Water misting | (Gurley et al. 2010) | Mining room and pillar mining areas | Continuous miner | Thermo-Electron personal dust monitors. Gravimetric samplers. | At the intake - upwind of the CM At the CM operator’s position. At the haulage-unit operator’s position  At the return - downwind of the CM. | Not stated |
|  | Water misting | (Ren et al. 2013) | Coal mining face | Hydraulic supports | Approved universal pump and sample heads according to AS2985 | Mine A - at MG Drive, Chock No 8 and shadowing shearer operators. Mine B - at the maingate (BSL) and at Chock No 6. | Not stated |
|  | Water misting | (Zongyin 2013) | Level crushing station of underground (UG) copper mine | Crusher in the UG mine | Not stated | Not stated | Not stated |
|  | Water misting | (Ren, T et al. 2014) | Coal mine roadway | N/A | The ‘TSI Dust Trak 2’ Aerosol Monitor | Adjacent to #007 belt at top of MT01 drift Adjacent to #030 belt, #060 belt, 090 belt and 109 belt. Between Magnet & LTU. 14CT Splicing Station. Between 19 & 20 C/T. 10m outbye 2nd Tripper 51 C/T. | Not stated |
|  | Water misting | (Nie, Ma, et al. 2016) | Coal mining face | Coal mining machine | Not stated | At the coal mining machine driver’s position. 10 m on the lee side of the coal mining machine. Hydraulic support advancing workers area. The position of scraper conveyer tip. At the crushing machine; Near the reversed loader. 30m along the air return way. | Not stated |
|  | Water misting | (Zhou, G et al. 2020) | Coal mining face | Advancing support | Dust filter membrane | Dust sampled at 10, 15, 20, 25, 30 and 35m behind the advancing support. | Not stated |
|  | Water misting | (Sun et al. 2019) | Coal mining face | Hydraulic supports and the shearer | Not stated | Hydraulic support advancing workers area. The shearer operator’s position. 5 m on the leeward side of the shearer. At the position of conveyer and 10 m away from the upwind side of conveyer. At the crushing machine. Near the reversed loader. | Not stated |
|  | Water misting | (Roberts & Wypych 2017) | ROM bin in an iron ore mine | ROM bin | Not stated | Not stated | Not stated |
|  | Water misting | (Peng, Huitian et al. 2020) | Coal mine roadway | Road header | AKFC-92A dust sampler | The road header operator’s position  The reversed loader,  At points 50 and 100 m from the heading face | Not stated |
|  | Water misting | (Han et al. 2014) | Coal mining face | EBZ132 road header | Not stated | At the position of the heading machine operator. | Not stated |
|  | Water misting – air assisted | (Peng, H. et al. 2019) | Coal mining face | Coal shearer and hydraulic support | AKFC-92 | Not stated | Not stated |
|  | Water misting – air assisted | (Wang, Y et al. 2019) | Iron or mining - ore pass | N/A | Not stated | Near the wellhead of ore pass on the fourth level crosscut | Not stated |
|  | Water misting - air assisted | (Peng, Huitian et al. 2019) | Coal mining face | Coal shearer | TSI9306 dust counter | At the shearer driver’s position. 10 m to the leeward side of shearer driver’s position.  Hydraulic support advancing workers area. | Breathing zone of the employees (1.6m) |
|  | Water misting – spray nozzles | (Wang, J et al. 2019) | Coal hydraulic support area | Hydraulic support system | An AKFC-92A dust sampler | 5.0 m downstream of multi-nozzle before and after spray dust suppression | Not stated |
|  | Water misting – spray nozzles | (Gao et al. 2018) | Coal mine roadway | Not stated | Not stated | Not stated | Not stated |
|  | Water misting – spray nozzles | (Han & Liu 2018) | Coal mine roadway | Road header | A direct reading dust detector | At the road header driver’s position | 1.7 m above the floor |
|  | Water misting – spray nozzles | (Cheng et al. 2016) | Coal mining face | Double-ended shearer. An EBZ-160TY road header. | An AKFC-92A mine dust sampler | Near the shearer drum and cutting head of the road header.  At the operator’s position. At the support advancing area. Drawing opening Crusher Stage loader Return airway Stage loader  100 and 200m away from the driving head. | Not stated |
|  | Water misting – spray nozzles | (Zhou, G, Xu, M, et al. 2017) | Coal mining face | Shearer | Not stated | Not stated | Not stated |
| Water based dust control | Wet type dust extraction | (Ren et al. 2020) | Coal mine roadway | Digging Machine | Not stated | At the driver’s position. 20 m behind the drilling machine. | Not stated |
|  | Wet type dust extraction | (Reed, Shahan, Ross, et al. 2020) | Coal mining areas | Underground roof bolter machine | CMDPSU gravimetric samplers. pDR-1000 instantaneous sampler. | The intake sampler at the entry upwind of the roof bolter.  The return sampler at the entry of the ventilation exhaust tubing inlet.  The rear right-side corner of the roof bolting machine. Above the front and rear dust collector box doors On the right side near the right side pre-cleaner dump and on the left side near the left side pre-cleaner dump. Sampling vest is worn by the operator | In the centre of the entry. 106–122 cm above the mine floor. |
|  | Wet type dust extraction | (Shi et al. 2013) | Mineral mine tunnel | Not stated | As per GBZ/T192.2-2007 and GBZ/T192.4-2007 standards. | In the front of the fan and in the rear of the wet dust precipitator | Not stated |
|  | Wet type dust extraction | (Warden & Warden 2019) | Tunnelling | Road Header | Not stated | Various locations – not stated | Not stated |
|  | Wet type dust extraction | (Li, Y et al. 2020) | Thermal power plant | N/A | Not stated | Not stated | Not stated |
|  | Wet type dust extraction | (Colinet et al. 2013) | Coal mining face | Continuous miners (CM) | Thermo Scientific Model pDR-1000AN Personal Data Rams (pDRs).  Thermo Scientific Model PDM3600 Personal Dust Monitor (PDM). | Immediate intake and return for the CM The right rear corner of the CM In the shuttle car cab.  In the bolter intake and on-board the bolter | Not stated |
|  | Wet type dust extraction | (Xia et al. 2016) | Coal bulk handling plant | Coal transfer chute | Not stated | Not stated | Not stated |
|  | Wet type dust extraction | (Zhou, G, Zhang, Q, et al. 2017) | Coal excavation face | Shearer and hydraulic support | AKFC-92A dust sampler | At the leeward side of the advancing support. The position of the shearer’s driver | 1.55m from the base height of 0.4m. |
|  | Wet type dust extraction | (Xie et al. 2012) | Coal mine roadway | Coal cutting machine | Not stated | The working face  Operator’s position  The end of conveyor | Not stated |
|  | Wet type dust extraction | (Liu et al. 2020) | Coal tunnelling face | Tunnelling Machine | AKFC explosion-proof dust sampler. | Working face. Exhausting air inlet. The operator’ position. Middle part of the overlap. Purified air outlet. 10m after dust collector. | Not stated |
|  | Wet type dust extraction | (Zhou et al. 2013) | Coal mining face | Entry driving machine | N/A | N/A | N/A |
|  | Wet type dust extraction | (Li, G et al. 2020) | Metal mining transfer station | Not directed at an equipment | TH-880 F dust-measuring instrument | At the blanking area of number 8 and number 9 belt conveyor  At the end unit of number 9 and number 10 belt conveyor. | 1.5 m |
|  | Wet type dust extraction | (Zhou, G et al. 2018) | Coal mining face | Coal cutter (shearer) | AKFC-92A dust sampler | In front and behind the coal cutter driver.  0 – 20m on the leeward side of the driver. | Not stated |
| Wetting | Wetting - Dust source is made wet before using | (Li, P et al. 2019) | Coal mine roadway | N/A | The filter weighing method was used for dust measurement. | About 3 ∼ 5m from the nozzle  Central part of the shotcreting zone  1m, 4 ∼ m and 19 ∼ 35m from the end of Wet or dry spraying and mixer machine | Not stated |
| Mixed Method | Wetting  Water misting | (Shepherd & Woskie 2013) | Construction - field-lab experiment | Two-stroke gas engines saws with 14-in. diameter blades. | Thermo DataRAM pDR1200  A GilAir- 5 personal sampling pump | Not stated | Not stated |
|  | Wet dust control  LEV | (Middaugh et al. 2012) | Construction site | A gas-powered cut-off saw.  A 35.6-cm diameter, diamond cutting blade. | SKC sampling pump with aluminum cyclone. 5 μm pore size, preweighed PVC filters loaded in three-piece 37-mm cassettes. | On the left lapel of the operator. | Breathing zone of employees |
|  | General ventilation Dust extraction Water wetting | (Akbar-Khanzadeh et al. 2010) | Construction - field-lab experiment | Concrete grinding Equipment. | Portable personal pump | Not stated | Not stated |
|  | General method  Water misting | (Lin et al. 2011) | Foundry (iron) | N/A | Personal SKC air sampling pumps | Fixed on research participants | Breathing zone of participants. |
|  | LEV  Isolations  Standard operating procedures. | (Firdaussyah & Suryo 2018) | Steel fabrication plant | Sandblasting room | NIOSH 7500 for collecting silica dust in ambient air | Eight sampling points located around the sandblasting room. | Not stated |
|  | Ventilation (OASIS, LEV). Use of bag valve type of bags and dual nozzle.  Bag belt cleaning device.  Semi-automated bag palletizing with hydraulic lift. Daily housekeeping  Wet floor cleaning | (Louk et al. 2020) | Industrial sand mining (Bagging and pelleting) | On the material been worked on. | Thermo Scientific pDR-1000 instantaneous respirable dust monitor | Eight locations were sampled in Operation 1  Eight locations were sampled in Operation 2  Nine locations were sampled in Operation 3  Six locations were sampled in Operation 4 | Not stated |
|  | Foam spray system  Water Mist | (Wang et al. 2020) | Coal mine roadway | Road Header | Not stated | Driver’s position | Not stated |
|  | Various dust control methods as part of a management system | (Shang 2014) | Port and shipping industries (Bulk material storage and handling) | Various | Not stated | Cape ship dock N1, ore dock N2, inside the storage yard N3, village nearby the factory N4, main road outside the port N5). | Not stated |
|  | LEV  Wetting  Water misting | (Qi & LO 2016) | Stone countertop fabrication and installation industry | Pneumatic/electric wet grinders.  Pneumatic wet polishers. | FL battery-operated sampling pump. NIOSH Method 7500. | Personal samplers carried by workers | Breathing zone of employees |
|  | Water injection  Surfactant water misting | (Wang, K et al. 2016) | Coal mine roadway | Not stated | Not stated | At the position of the mining machine driver. 10m and 100m away from the tunnelling face. | Not stated |
|  | Air curtain  Wet dust extraction | (Zhou, Nie, et al. 2012) | Coal mining face | Coal winning machine | Not stated | Not stated | Not stated |
|  | Dust collecting net, dust diffusion and water sprays | (Chen et al. 2018) | Coal mine roadway | A double drum coal cutter. | Not stated | At 2m in front and behind the dust collecting net. | Breathing zone of employees (1.55 to 2.25m) |
|  | Behavioural Intervention  Other control types | (Patts et al. 2020) | Metal and non-metal mines | N/A | pDR-1500 real-time aerosol monitor | On the operator | Breathing zone of employees |
