# Supplementary material for: A systematic review of the effectiveness of dust control measures adopted to reduce workplace exposure
Source: Environ Sci Pollut Res Int. 2023 Mar 25;30(19):54407–28. doi: 10.1007/s11356-023-26321-w (PMC10121514; doi:10.1007/s11356-023-26321-w)
Supplement: Supplementary file 4 — Supplementary file4 (DOCX 188 KB) [file 11356_2023_26321_MOESM4_ESM.docx]

**SUPPLEMENTARY SHEET 4**

A systematic review of the effectiveness of dust control measures adopted to reduce respirable crystalline silica exposure in the workplace

**Factors influencing the effectiveness of dust control interventions adopted at the workplace**

| **Control Methods** | **Factors affecting efficiency** | **Study** |
| --- | --- | --- |
| Any method | Environmental factors - size of focus area, wind velocities, water consumption/availability, physical barriers, hazards (such as machine or equipment movements, flying rock etc) | (Garcia et al. 2014; Roberts & Wypych 2017) |
|  | Dust properties - dust particle size distribution, velocity, concentration | (Chen et al. 2018; Cheng et al. 2016; Roberts & Wypych 2017) |
| Air curtain | Relative positions of ventilation facilities | (Zhou, Nie, et al. 2012) |
|  | Pressurized air flow rate | (Hua et al. 2020; Zhou, W et al. 2020) |
|  | Air extraction flow rate | (Hua et al. 2020; Liu, Nie, Hua, Jia, et al. 2019; Zhou, W et al. 2020) |
|  | Diameter of the pressurized air duct | (Zhou, W et al. 2020) |
|  | Inflow-to-outflow ratio | (Hua et al. 2020; Zhou, Nie, et al. 2012) |
|  | Axial radial ratio of the forced air | (Fang et al. 2019) |
|  | Relative position of turbulator and the second exhaust air outlet from the heading face | (Chen & Liu 2019) |
|  | Distance from the pressure ventilation outlet to the heading face | (Liu, Nie, Hua, Peng, et al. 2019; Nie, Liu, et al. 2016) |
|  | The wind velocity vector of the air curtain generator | (Cheng, Nie, Zhou, Yang, et al. 2012) |
|  | Effectiveness of the drawer-type air dryer collector | (Zhou, Wang, et al. 2012) |
|  | Position of the air curtain generator from the tunnel face | (Liu et al. 2018; Yin et al. 2019) |
| Surfactant-based interventions | Viscosity of the surfactant | (Hu et al. 2020; Liao et al. 2018) |
|  | Dust fixation ability (water retention ability of the suppressant) | (Bao et al. 2020; Hu et al. 2020) |
|  | Wettability of the suppressant | (Bao et al. 2020; Hu et al. 2020; Liao et al. 2018; Wang, K et al. 2019; Wang, K et al. 2016; Wang, X et al. 2019) |
|  | Wind erosion resistance of the surfactant | (Bao et al. 2020) |
|  | Addition of ionic liquid to the wetting agent | (Wang, K et al. 2019) |
|  | The solidifying bonding effect | (Bao et al. 2020) |
|  | Flow rates of the water surfactant mixture | (Echt et al. 2016; Wang et al. 2018; Zhou et al. 2019) |
|  | Water velocity through the magnetic field for magnetised surfactant water | (Zhou, Q et al. 2017) |
|  | Critical micelle concentration | (Wang, K et al. 2016) |
|  | Intensity of the magnetic field in a magnetised surfactant water | (Zhou, Q et al. 2017) |
| Reverse circulation air hammer drill bit | Suction capacity (dependent on defection angle, vertical space, horizontal space) | (He et al. 2018; Yin et al. 2013) |
|  | Number of suction nozzles or inclination angles of suction nozzles  Position of the suction nozzles in the drill bit body | (Yin et al. 2013) |
| Wet Shotcrete spray | Spraying angle  Velocity, spray distance and the application of additives. | (Li, P et al. 2019) |
| Water curtain | Angle of tilt of the venturi negative-pressure secondary dedust device in the curtain arrangement | (Sun et al. 2019) |
| Water misting interventions | Particle size of the water mist | (Ge et al. 2019; Ma et al. 2020; Nie et al. 2017; Nie, Ma, et al. 2016; Peng, Huitian et al. 2019; Sun et al. 2019; Wang, Y et al. 2019; Xia et al. 2016; Yang et al. 2019; Zongyin 2013) |
|  | Droplet concentration | (Guo et al. 2020; Li et al. 2020; Ma et al. 2020; Peng, H. et al. 2019; Peng, Huitian et al. 2019) |
|  | Droplet distribution or coverage | (Gao et al. 2018; Ge et al. 2019; Peng, H. et al. 2020; Sun et al. 2019; Wallace & Cheung 2013; Zhou, G et al. 2017; Zhou, G et al. 2020) |
|  | Droplet velocity | (Guo et al. 2020; Peng, Huitian et al. 2019; Wallace & Cheung 2013) |
|  | Spray pattern | (Nie, Ma, et al. 2016; Wallace & Cheung 2013) |
|  | Spray range | (Li et al. 2020; Zhou et al. 2018; Zhou, G et al. 2020) |
|  | Spray pressure | (Cheng et al. 2016; Gao et al. 2018; Guo et al. 2020; Gurley et al. 2010; Jian et al. 2012; Nie et al. 2017; Nie, Ma, et al. 2016; Peng, H. et al. 2019; Sun et al. 2019; Wallace & Cheung 2013; Wang, J et al. 2019; Wang et al. 2018; Xu et al. 2019; Yang et al. 2019; Zhou, G et al. 2017) |
|  | Water flow rate | (Gurley et al. 2010; Wang, J et al. 2019; Wang, Y et al. 2019; Zhou et al. 2018; Zhou, G et al. 2020; Zongyin 2013) |
|  | Atomisation angle | (Gao et al. 2018; Guo et al. 2020; Jian et al. 2012; Ren et al. 2014; Wang, J et al. 2019; Zhou, G et al. 2020) |
|  | Angle of conical cover over the nozzle and its length in the external spray negative-pressure mist-curtain dust suppression device | (Peng, Huitian et al. 2020) |
|  | Impact of airflow or speed in the work area | (Chen et al. 2018; Han et al. 2014; Peng, H. et al. 2019; Ren et al. 2014; Wang, Y et al. 2019; Warden & Warden 2019; Zhou, G et al. 2017) |
|  | Air pressure in air assisted water misting | (Hu et al. 2019; Peng, H. et al. 2019; Ren et al. 2013; Wang, Y et al. 2019) |
|  | The side suction port of the spray dust-settling device | (Xu et al. 2020) |
| Wet dust extraction systems | Inlet and outlet inclination angles of the Coanda effect extraction device  Number of orifices on the Coanda effect extraction device | (Ren et al. 2020) |
|  | Liquid-gas ratio | (Li et al. 2020) |
| Interventions using spray nozzles | Type of spray nozzle | (Han & Liu 2018; Han et al. 2016; Jian et al. 2012; Li et al. 2020; Roberts & Wypych 2017; Sun et al. 2019; Wallace & Cheung 2013; Wang et al. 2015; Zhou, G et al. 2020) |
|  | Number of spray nozzles used in the intervention | (Jian et al. 2012; Ren et al. 2020) |
|  | Nozzle installation position | (Nie et al. 2017; Peng, Huitian et al. 2020; Ren et al. 2014; Ren et al. 2020; Roberts & Wypych 2017; Yang et al. 2019) |
|  | Arrangement of the nozzles at the work face | (Ren et al. 2013; Sun et al. 2019; Zhou et al. 2019) |
|  | Spray angle | (Li et al. 2020; Zhou et al. 2018) |
|  | Spray distance (distance of spray nozzle to dust source) | (Ren et al. 2013; Zhou, G et al. 2017; Zhou et al. 2019) |
|  | Nozzle diameter | (Gao et al. 2018; Xu et al. 2019; Xu et al. 2020) |
|  | Atomisation quality of the nozzle | (Zhou, G et al. 2017) |
| Chute with wet extraction system | Air Velocity in the chutes  Loading angle of the belt  The flow ability of coal | (Xia et al. 2016) |
| Dry extraction system in a plant | Environmental factors outside the workplace (wind speed)  Position of the installation of the ventilation system Weight of the pollutant  Distance of the hood from the dispersed source  Speed of the dispersed particles | (Zarei et al. 2018) |
| Dry dust extraction intervention used in mine roadways or tunnels | The position of the inlet of the exhaust dust from the dust source | (Li, S et al. 2019; Li et al. 2017) |
|  | The air-draft volume of dedusting fan | (Zhou et al. 2013) |
|  | The position of the outlet of the forced air | (Li, S et al. 2019) |
| Dry dust extraction in an enclosed space | Well-designed dust extraction system | (Zhang et al. 2014) |
|  | The ventilation rate in the enclosure | (Kokkonen et al. 2019; Kokkonen et al. 2017; Zhang et al. 2014) |
|  | Airtightness of the enclosure and the continuous maintenance of negative pressure | (Firdaussyah & Suryo 2018; Kokkonen et al. 2019; Kokkonen et al. 2017) |
|  | Wind direction | (Potts & Reed 2011) |
| Dust Collector | The type of material used to design the mini bag house  Filter material of the mini bag house  Clamping mechanism of the mini bag house | (Alexander et al. 2016) |
| Dust purify fan for a shearer | Extraction rate of the fan  Position of the fans on the rocker arm of the shearer | (Cai et al. 2020) |
| Roof bolter canopy air curtain | Relative position of the bolter operators | (Reed et al. 2020) |
| Foam technology | Type or formula of the foaming agent or its wetting performance | (Chen et al. 2015; Ren et al. 2012; Wang et al. 2015) |
|  | Adhesion energy of the foam | (Guo et al. 2019) |
|  | Dust particle size | (Zhu et al. 2020) |
|  | Performance of the foaming agent adding device | (Lu, X-x et al. 2015; Lu et al. 2019; Wang et al. 2012) |
|  | The level of cavitation generated | (Lu et al. 2019; Wang et al. 2012) |
|  | Bubble diameter of the foam | (Lu et al. 2017) |
|  | The critical outlet pressure of the foam mixing device (generator) | (Wang, D et al. 2016) |
|  | Foam expansion ratio | (Lu, X et al. 2015; Wang et al. 2013) |
|  | Speed of ventilating air | (Wang et al. 2014) |
|  | Stable water and air flow to draw in foaming agent | (Wang et al. 2011) |
| Water infusion | Distribution of the abutment pressure | (Hu et al. 2016) |
|  | Water injection pressure, pore rate, coal permeability coefficient | (Cheng, Nie, Zhou, Yu, et al. 2012) |
|  | Distance for water injection ahead of the working face | (Hu et al. 2016) |
